# Changes in life expectancy and disease burden in Norway, 1990–2019: an analysis of the Global Burden of Disease Study 2019

**DOI:** 10.1016/S2468-2667(22)00092-5

**Published:** 2022-06-29

**Authors:** Benjamin Clarsen, Magne Nylenna, Søren Toksvig Klitkou, Stein Emil Vollset, Carl Michael Baravelli, Anette Kocbach Bølling, Gunn Marit Aasvang, Gerhard Sulo, Mohsen Naghavi, Maja Pasovic, Muhammad Asaduzzaman, Tone Bjørge, Anne Elise Eggen, Terje Andreas Eikemo, Christian Lycke Ellingsen, Øystein Ariansen Haaland, Alemayehu Hailu, Shoaib Hassan, Simon I Hay, Petur B Juliusson, Adnan Kisa, Sezer Kisa, Johan Månsson, Teferi Mekonnen, Christopher J L Murray, Ole F Norheim, Trygve Ottersen, Dominic Sagoe, Kam Sripada, Andrea Sylvia Winkler, Ann Kristin Skrindo Knudsen

**Affiliations:** a Department of Disease Burden, Norwegian Institute of Public Health, Bergen, Norway; b Department of Health Register Research and Development, Norwegian Institute of Public Health, Bergen, Norway; c Oslo Sports Trauma Research Center, Department of Sports Medicine, Norwegian School of Sport Sciences, Oslo, Norway; d Department of Health and Functioning, Faculty of Health and Social Sciences, Western Norway University of Applied Sciences, Bergen, Norway; e Department of Health and Society, University of Oslo, Oslo, Norway; f Department of Community Medicine and Global Health, University of Oslo, Oslo, Norway; g Department of Nutrition, University of Oslo, Oslo, Norway; h Institute of Health and Society, University of Oslo, Oslo, Norway; i Centre for Disease Burden, Department of Air Quality and Noise, Oslo, Norway; j Division for Infection Control, Oslo, Norway; k Norwegian Institute of Public Health, Oslo, Norway; l Institute for Health Metrics and Evaluation, University of Washington, Seattle, WA, USA; m Department of Health Metrics Sciences, School of Medicine, University of Washington, Seattle, WA, USA; n Department of Global Public Health and Primary Care, University of Bergen, Bergen, Norway; o Center for International Health, University of Bergen, Bergen, Norway; p Bergen Center for Ethics and Priority Setting, University of Bergen, Bergen, Norway; q Department of Clinical Science, University of Bergen, Bergen, Norway; r Department of Psychosocial Science, University of Bergen, Bergen, Norway; s Cancer Registry of Norway, Oslo, Norway; t Department of Community Medicine, University of Tromsø, Tromsø, Norway; u Centre for Global Health Inequalities Research (CHAIN), Norwegian University of Science and Technology, Trondheim, Norway; v Department of Pathology, Stavanger University Hospital, Stavanger, Norway; w School of Health Sciences, Kristiania University College, Oslo, Norway; x Department of Global Community Health and Behavioral Sciences, Tulane University, New Orleans, LA, USA; y Department of Nursing and Health Promotion, Oslo Metropolitan University, Oslo, Norway; z Department of Global Health and Population, Harvard University, Boston, MA, USA; aa Department of Neurology, Technical University of Munich, Munich, Germany

## Abstract

**Background:**

Geographical differences in health outcomes are reported in many countries. Norway has led an active policy aiming for regional balance since the 1970s. Using data from the Global Burden of Disease Study (GBD) 2019, we examined regional differences in development and current state of health across Norwegian counties.

**Methods:**

Data for life expectancy, healthy life expectancy (HALE), years of life lost (YLLs), years lived with disability (YLDs), and disability-adjusted life-years (DALYs) in Norway and its 11 counties from 1990 to 2019 were extracted from GBD 2019. County-specific contributors to changes in life expectancy were compared. Inequality in disease burden was examined by use of the Gini coefficient.

**Findings:**

Life expectancy and HALE improved in all Norwegian counties from 1990 to 2019. Improvements in life expectancy and HALE were greatest in the two counties with the lowest values in 1990: Oslo, in which life expectancy and HALE increased from 71·9 years (95% uncertainty interval 71·4–72·4) and 63·0 years (60·5–65·4) in 1990 to 81·3 years (80·0–82·7) and 70·6 years (67·4–73·6) in 2019, respectively; and Troms og Finnmark, in which life expectancy and HALE increased from 71·9 years (71·5–72·4) and 63·5 years (60·9–65·6) in 1990 to 80·3 years (79·4–81·2) and 70·0 years (66·8–72·2) in 2019, respectively. Increased life expectancy was mainly due to reductions in cardiovascular disease, neoplasms, and respiratory infections. No significant differences between the national YLD or DALY rates and the corresponding age-standardised rates were reported in any of the counties in 2019; however, Troms og Finnmark had a higher age-standardised YLL rate than the national rate (8394 per 100 000 [95% UI 7801–8944] *vs* 7536 per 100 000 [7391–7691]). Low inequality between counties was shown for life expectancy, HALE, all level-1 causes of DALYs, and exposure to level-1 risk factors.

**Interpretation:**

Over the past 30 years, Norway has reduced inequality in disease burden between counties. However, inequalities still exist at a within-county level and along other sociodemographic gradients. Because of insufficient Norwegian primary data, there remains substantial uncertainty associated with regional estimates for non-fatal disease burden and exposure to risk factors.

**Funding:**

Bill & Melinda Gates Foundation, Research Council of Norway, and Norwegian Institute of Public Health.

## Introduction

Situated in northwestern Europe, Norway is an elongated, mountainous country, with a long coastline and over 230 000 islands. It is one of the most sparsely populated countries in Europe, with half of its 5·4 million citizens living in the southeast, including 1·5 million in the greater Oslo area.

According to the Global Burden of Disease Study (GBD) 2019, Norway is ranked among the top ten countries globally in terms of life expectancy at birth, healthy life expectancy (HALE), and age-standardised rate of disability-adjusted life-years (DALYs).^1^, ^2^ A previous study focusing on the disease burden in Nordic countries concluded that Norway has the same main causes of disease burden as the other Nordic countries—namely, neoplasms, cardiovascular diseases, and mental and musculoskeletal disorders, with smoking, alcohol use, and metabolic factors being important risk factors for disease burden.^3^

Norway is a stable social democracy with an open market economy, powerful labour unions, and high taxes. The country is rich in natural resources, particularly oil and gas, and has a gross domestic product of approximately US$71 000 per capita, and general government spending of 51·3%.^4^ Norway is consistently ranked among the wealthiest countries in the world and, with a Gini coefficient of 0·27, it is among the countries with the lowest income inequalities.^5^ The UN Development Programme puts Norway on the top of the Human Development Index on the basis of health, education, and income status in 189 countries.^6^

**Research in context**

**Evidence before this study**

We searched the databases Embase, Web of Science, SveMed+, Ovid MEDLINE, Epub Ahead of Print, In-Process & Other Non-Indexed Citations, and Daily and Versions, with the search terms “disease burden”, “mortality”, “morbidity”, “life expectancy”, “health”, “changes”, “development”, “Norway”, and “Norwegian counties” for articles published in English or any Scandinavian language from database inception to April 20, 2021. The search returned 4406 hits. The identified scientific studies compared differences in life expectancies between the Norwegian counties, and geographical differences in incidence and prevalence of specific diseases or categories and in exposure to various risk factors. None of the studies gave an overall comparison of disease burden between the Norwegian counties. The Norwegian Institute of Public Health (NIPH) publish statistics on main health indicators in the different counties. The statistics database contains statistics about health, illness, risk factors, and population in the Norwegian counties. NIPH also publishes annual public health profiles for each county, comparing important public health indicators, living conditions, and symptoms. The Global Burden of Disease Study (GBD) has published national estimates over disease burden in Norway since GBD 2013. To our knowledge, there has been no systematic overviews of gradients of disease burden and risk factors across the Norwegian counties.

**Added value of this study**

In this Article, which is based on data from GBD 2019, we provide a comprehensive overview of the changes in life expectancy and disease burden in Norwegian counties. Our results show little variation in life expectancy, healthy life expectancy, and age-standardised all-cause years of life lost, years lived with disability, and disability-adjusted life-years (DALYs) across Norwegian counties. Further, the regional variation in life expectancy in Norway has decreased over the past 30 years. Our study shows that the leading causes and risk factors for DALYs were similar across counties, and that the relative contribution of non-fatal causes of disease burden increased between 1990 and 2019 in all counties.

**Implications of all the available evidence**

The similarities in health challenges across the Norwegian counties implies that common policies and strategies should be used on both national and regional levels to improve mortality and morbidity in the Norwegian population. Norwegian policies should aim to maintain equal access to health services regardless of geographical location. Public health work is a key responsibility of the Norwegian municipalities. Although some important causes and risk factors for fatal disease burden have been well known for decades, the results from the present study show that the prevention of non-fatal causes of disease burden, as well as reduction of risk factors, should be key concerns in the public health work. This study does not look at within-county or socioeconomic inequalities in health, but other studies indicate that much work remains in reducing such differences in Norway.

Social security for the population is a key feature of the Norwegian welfare system and includes free access to higher education, a universal and predominantly publicly financed health-care system, and a social safety net for people with reduced health and income.^7^

**Figure 1:**
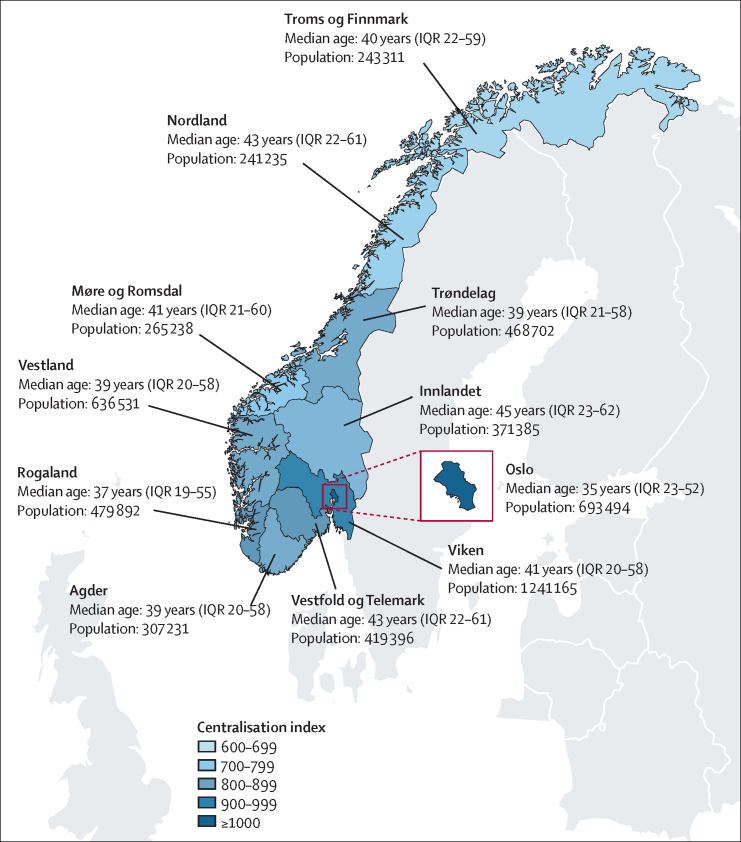
Map of Norway and the 11 counties, including the median ages and population numbers in 2019 The centralisation index shows each county's degree of population centralisation, based on an index of all 356 municipalities from the least centralised (Utsira, 295) to the most centralised in Norway (Oslo, 1000).^8^

Norway has an administrative system with 11 counties, known as fylker (figure 1), and 356 municipalities, known as kommuner. Each municipality is responsible for primary health care, with freedom in organising local services. The municipalities range in population, from 192 (Utsira) to around 700 000 (Oslo). The counties are larger entities, and differ greatly in terms of climate, settlement patterns, and main sources for economic activity. Therefore, the counties are interesting units for examining geographical differences in health.

Like other high-income countries, Norway is challenged by falling fertility rates, population ageing, rural-to-urban centralisation, and an increasing proportion of the population living with chronic diseases.^9^ Nevertheless, the population has grown in recent decades due to prolonged life expectancy and immigration. Since the 1970s, Norwegian governments have consistently pursued active policies to promote regional balance in economic growth, sustainability, commuting, and access to education and health services. Everyone has the right to the same level of health services regardless of personal financial situation and place of residence, according to the governmental health services plan.^10^ The responsibility for public health lies on the municipalities and is, therefore, decentralised across the country.

The Norwegian health-care system provides universal access to a broad benefits package, and public spending represents 86% of health expenditure—the highest share in Europe.^11^ The country has succeeded in establishing local villages and urban areas with employment opportunities, schools, shopping malls, and medical centres even in scarcely populated parts of the country.

Centralisation of the Norwegian population has primarily occurred regionally, with people moving from rural to urban areas within each county, as well as to the capital city.^12^ Norway currently has an urban density (proportion of the population living in urban settlements with at least 200 persons) of 82%,^13^ but with substantial variation in population centralisation between counties (figure 1) and municipalities (appendix p 8). The demographic composition also differs between counties. For example, the county with the oldest population, Innlandet, has a median age that is 10 years older than the county with the youngest population, Oslo (figure 1).

Geographical differences in life expectancy and mortality have been found in several countries.^14^, ^15^, ^16^, ^17^, ^18^, ^19^ Norway is no exception, although analyses based on data from Statistics Norway have shown decreasing county-wise variation in life expectancy from 1980 to 2014.^20^ Due to demographic and epidemiological transitions, including prolonged life expectancy, mortality is having a decreasing impact on the overall Norwegian disease burden. According to GBD 2019,^1^, ^2^ non-fatal health loss constitutes 53% of the total disease burden in Norway. Causes of morbidity and their attributable risk factors are, therefore, main public health challenges, particularly in people younger than 70 years. To ensure that health systems and public health and social policies align with the health challenges in the populations they are to serve, a comprehensive overview of the causes and risk factors of both mortality and morbidity (the total disease burden of a population), and how these change over time, is essential. Despite great political interest in regional health inequality, no such comprehensive overview has been published for Norway. From an international perspective, it is of interest to explore regional health differences in a country that has pursued an active regional policy, such as Norway.

The possibility to do comparative analyses is a core feature of the Global Burden of Disease, Injuries and Risk Factor Study (GBD), and the data are therefore particularly well suited to examine geographical differences in disease burden. A number of countries with varying incomes and health-care challenges have done systematic analyses of GBD at a subnational level, such as Brazil,^18^ China,^14^ India,^21^ Japan,^17^ Kenya,^19^ Mexico,^15^ South Africa,^22^ and the UK.^16^, ^23^ With GBD 2019, subnational results showing disease burden in the 11 Norwegian counties are available for the first time.^24^ The aim of this study is to examine the development in life expectancy, HALE, and overall cause and risk-factor specific disease burden between the Norwegian counties from 1990 to 2019, with a particular focus on regional inequalities within Norway.

This manuscript was produced as part of the GBD Collaborator Network and in accordance with the GBD Protocol.^25^

## Methods

### Overview

GBD analyses adhere to the Guidelines for Accurate and Transparent Health Estimates Reporting (GATHER) standards.^26^ The methods used in GBD 2019 are described in detail in the capstone papers and their supplements.^1,2,27–29^ GBD uses several metrics to describe disease burden, including number of deaths, YLLs, YLDs, and DALYs. Life expectancy and HALE are also calculated. GBD aims to use all available evidence as the basis for these estimates. The estimates were calculated in a cascade model in the following order: global, super-region, region, country, and subnational. Super-region priors were generated at the global level with mixed-effects non-linear regression using all available data. The super-region fit then informed the region fit, and this pattern continued down the cascade.^1^ The subnational estimation was informed by the country fit and country covariates, in addition to adjustments based on the average of the residuals between the subnational unit's available data and priors.^1^

Each iteration of GBD includes new data sources and methodological advancements, and the entire time series of results is therefore reanalysed with each new iteration. In GBD 2019,^1^ disease burden was estimated for 286 causes of death, 369 diseases and injuries, and 87 risk factors for 990 geographical units by sex, age, and year. Geographical locations, causes, and risk factors were organised into increasingly detailed hierarchical groups.^1^, ^28^ In Norway, the subnational units were the 11 counties.^25^ Causes were organised into four levels: level one consisted of three categories (non-communicable diseases; injuries; and communicable, maternal, neonatal, and nutritional diseases), level 2 consisted of 22 subcategories of these, and levels 3 and 4 contained increasing levels of detail for specific disease and injury types. Risk factors were similarly organised; level 1 consisted of three broad categories (metabolic risks, behavioural risks, and environmental or occupational risks), level 2 consisted of 20 subcategories of these, and levels 3 and 4 contained increasing levels of detail for specific types of risks.^1^

### Mortality, causes of death, life expectancy, and YLLs

GBD obtains mortality data from the Norwegian Cause of Death Registry.^30^ These data are based on death certificates and provide information about the date and underlying cause of death, which is coded according to the International Classification of Diseases (version 9 for the period 1990–95, and version 10 from 1996 onwards). These data are generally considered to be of high quality;^30^ however, around 15% of the deaths are coded with unspecified diagnoses or codes that cannot be underlying causes of death (major and minor garbage codes, such as heart failure). Such codes were redistributed to valid death codes according to algorithms.^31^ GBD uses Cause of Death Ensemble modelling to estimate causes of death by age, sex, geographical location, and time.^32^ In GBD 2019, YLLs were estimated by multiplying each death with remaining years per age-specific reference life expectancies. The life expectancy cause-specific decomposition method, developed by Beltran-Sanchez, Preston, and Canudas-Romo, was used to examine national and county-wise changes in life expectancy at birth between 1990 and 2019.^33^

### Disease and injury incidence, prevalence, and YLDs

Norwegian data for prevalence and incidence of non-fatal causes of disease burden in GBD 2019 were obtained from systematic searches and reviews of published and unpublished data, including survey and inpatient data, and from input and transfer of data from Norwegian experts. The included data sources were catalogued in the Global Health Data Exchange platform.^24^ The Norwegian data sources used to quantify non-fatal outcomes are listed in the appendix (pp 27–57). The most important sources for Norway were administrative population registries, health registries (eg, the Norwegian Medical Birth Registry), the Norwegian Surveillance System for Communicable Diseases, the Norwegian Patient Registry, the Cancer Registry, large national and county-level health surveys (eg, the Cohort of Norway Survey, the Norwegian Health and Living Condition Survey, the Trøndelag Health Study, and the Tromsø Study), statistics on alcohol and tobacco sales, as well as statistics from international organisations (eg, UNICEF, UN, and the European Monitoring Centre for Drugs and Drug Addiction). Where Norwegian data are sparse, such as for mental and musculoskeletal disorders, estimates are modelled using available data from other countries in combination with known data for the Norwegian population. The Bayesian meta-regression tool DisMod-MR 2.1 was used to produce internally consistent estimates of incidence, prevalence, excess mortality, and remission.^34^ Assumed independent comorbidity was factored into the estimates. Disability weights were developed within GBD to quantify health loss associated with non-fatal causes.^35^ YLDs were calculated as the product of prevalence or incidence of each cause and the associated disability weight.

### DALYs, HALE, and attributable risks

In this analysis, National and county-wise DALYs were computed by summing YLLs and YLDs. HALE was calculated using multiple-decrement life tables and estimated YLDs per capita.^1^

The selection of risk–outcome pairs included in GBD was based on convincing or probable evidence of a causal relationship, and the relative risks in these pairs were estimated based on meta-regression from systematic reviews of the literature. Exposure levels by age, sex, location, and year were estimated on the basis of all available data sources but by using different methods dependent on the specific risk factors. The population attributable fraction was estimated by comparing the burden due to the current level of risk factor distribution with the hypothetical burden due to the theoretical minimum risk exposure level distribution, taking mediation between risk factors into account.^28^ The population attributable fraction represents the proportion of all-cause DALYs that could have been avoided had the exposure to the specific risk factor been equal to the theoretical minimum risk exposure level.^28^ The summary exposure values were calculated as the extent of exposure to each severity level of the risk factor in the population. The summary exposure values can range from 0% to 100%, in which 0% equals no excess risk in a population, and 100% means that the entire population is exposed to the highest risk level.^36^

### Inequality in health

Gini coefficients were calculated to assess the relative inequality between counties in life expectancy, HALE, level-2 causes of DALYs in 2019, and level-1 DALY causes and risk factors between 1990 and 2019.

In this context, the Gini coefficient can be defined as the divergence of a health variable between different counties from an equal distribution and is interpreted as half the relative difference between any two counties. The coefficient relates closely to a Lorenz curve, which depicts the cumulative percentage of the outcome against the cumulative ordering of counties, starting off with the county with the lowest rate. The Gini coefficient measures the area between this Lorenz curve and the line of complete equality (45° line). Therefore, a Gini of 0 represents a situation of complete equality (every county has the same DALY rate), whereas a Gini of 1 corresponds to a situation of complete inequality (all DALYs are confined to one county).

The Gini coefficient can be formulated as:

$$\frac{2}{\mu }\times \frac{\sum \left(\mu_{k}-\mu )\times (R_{k}-R\right)}{N}$$

where μ and μ_k_ is the grand and county-specific mean of the health variable, and *R*_k_ is the rank of a specific county relative to the average rank R̄, 0·5.

Gini coefficients were calculated using the Ineq package in R (version 3.6.1; R Core Team, 2013). CIs and estimates of Gini coefficients were calculated by assuming a normal distribution and resampling one thousand replicates from the mean and standard error (implied by the width of the 95% uncertainty interval [UI]) of the GBD data.

### Statistical analysis

Results were presented for all-causes combined, all level-2 causes, and the top ten level-3 causes and level-2 risk factors in each county. Unless otherwise stated, all results were presented as age-standardised rates to facilitate comparisons between counties with different age structures. Age standardisation was derived from world population standards developed for GBD 2019.

Uncertainty in data inputs, estimated model parameters, and bias-correction procedures were derived by generating 1000 draws at the level of age, sex, location, and year for each of the measures carried through the many GBD multistep estimation processes (population, mortality, migration, fertility,^2^, ^37^, ^38^ cause of death,^27^ non-fatal estimation,^27^ and comparative risk assessment). This approach captures uncertainty in each modelling stage and propagates it through the entire estimation process. Point estimates were computed as the mean of 1000 draws from the corresponding final (posterior) draw distribution and 95% UIs were computed with use of the 2·5 and 97·5 percentiles.

To compare differences in rates, rate ratios (RRs) and 95% CIs were calculated using the fmsb package in R. We considered rates to be significantly different if their CIs did not overlap.

### Role of the funding source

The funders of the study had no role in study design, data collection, data analysis, data interpretation, writing of the present report, or decision to publish.

## Results

**Figure 2:**
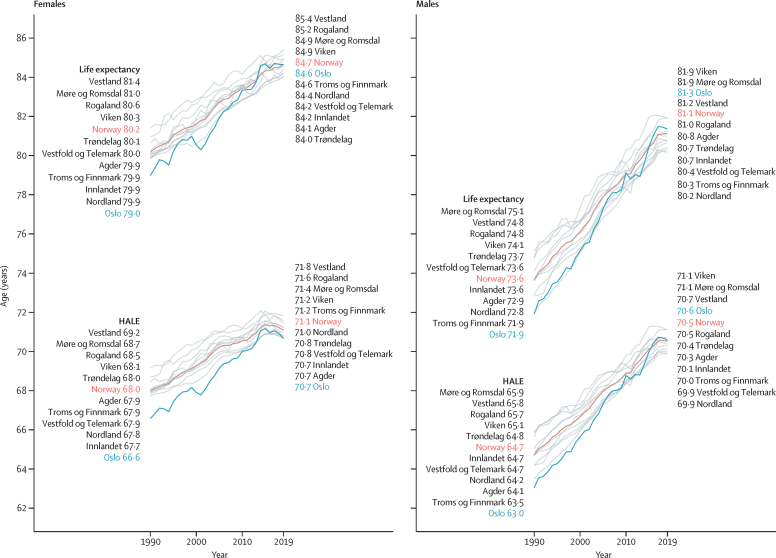
Change in life expectancy and HALE at birth by male and female sex in Norway and every Norwegian county, 1990–2019 Oslo is shown in blue to highlight its large change in rank during this period. 95% uncertainty intervals are shown in the appendix (p 3). HALE=healthy life expectancy.

**Figure 3:**
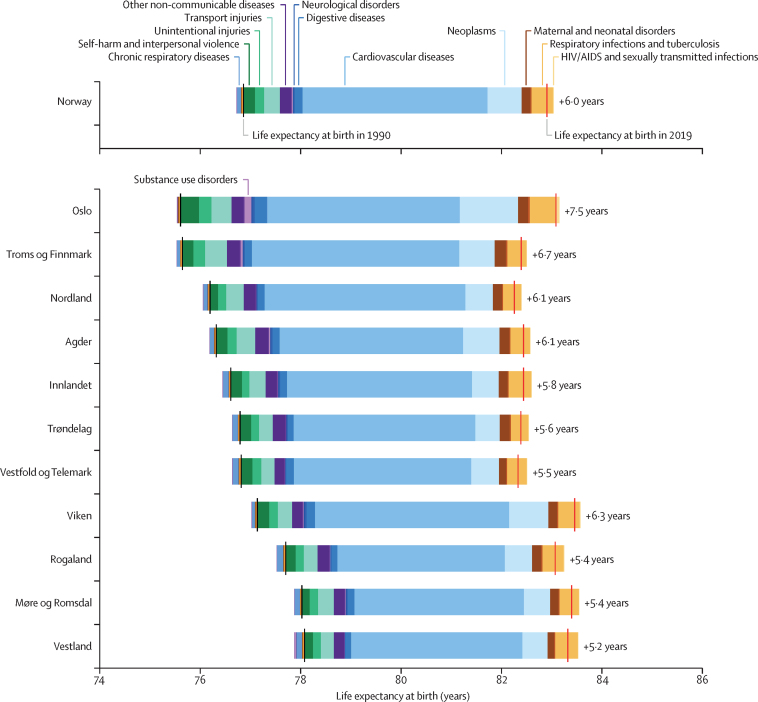
Change in life expectancy at birth in Norway and the 11 Norwegian counties between 1990 and 2019, decomposed into the contribution of GBD level-2 cause groups for male and female sexes combined Data for males and females separately are shown in the appendix (pp 9–10). Causes to the left of the 1990 life expectancy values reflect causes that contributed to reduced life expectancy between 1990 (black lines) and 2019 (red lines). Causes to the right of the 1990 life expectancy values reflect causes that contributed to increased life expectancy between 1990 and 2019. GBD=Global Burden of Diseases, Injuries, and Risk Factors Study.

Between 1990 and 2019, life expectancy at birth improved in all Norwegian counties for male and female sexes (figure 2). Improvements were greatest in Oslo, where life expectancy at birth increased from 79·0 years (95% UI 78·5–79·4) in 1990 to 84·6 years (83·7–85·6) in 2019 for females, and from 71·9 years (71·4–72·4) in 1990 to 81·3 years (80·0–82·7) in 2019 for males (figure 3, appendix p 3). Regional differences in life expectancy were smaller in 2019 than in 1990. The difference between the counties with the highest and lowest life expectancies in 1990 was 3·2 years for males and 2·4 years for females, and in 2019 the corresponding differences were 1·7 years and 1·4 years (figure 2). Although improvements in life expectancy were generally greater in males, their life expectancy remained 3·0–4·3 years lower than for females in 2019 across all counties.

There was an upward trend in HALE for males and females in every county between 1990 and 2019 (figure 2, appendix p 3). Improvements in HALE appear to have plateaued for females over the past decade, such that differences between male and female sexes in 2019 were 1·2 healthy years or fewer in all counties.

In all counties, improvements in life expectancy were mainly because of reductions in mortality from cardiovascular disease, neoplasms, and respiratory infections (figure 3).

All-cause YLL, YLD, and DALY rates for 2019 are presented for Norway and each county in the appendix (crude rates are shown on p 4 and age-standardised rates are shown on p 5). For YLDs and DALYs, crude and age-standardised rates were similar to the national rates for male and female sexes in all counties. Crude YLL rates were higher than the national rate (ie, UIs did not overlap) for males, females, and male and female sexes combined in Innlandet, Vestfold og Telemark, and Nordland, and for males in Troms og Finnmark. Crude YLL rates were lower than the national rate for males, females, and male and female sexes combined in Oslo, as well as for females and male and female sexes combined in Rogaland. After age-standardisation, the only county with YLL rates that differed from the national rate was Troms og Finnmark, which had a higher YLL rate for males and for male and female sexes combined.

The six leading causes of age-standardised YLLs were ischaemic heart disease, lung cancer, self-harm, stroke, colon and rectum cancer, and chronic obstructive pulmonary disease across all counties (appendix p 7). Only minor variation occurred in the ranking of these causes between counties, with YLL rates significantly higher than the national rate for lung cancer in Agder (RR 1·24 [95% CI 1·11–1·40]), self-harm in Innlandet (1·20 [1·05–1·36]) and Vestfold og Telemark (1·19 [1·05–1·36]), drug use disorders in Oslo (1·41 [1·18–1·70]), road injuries in Møre og Romsdal (1·25 [1·01–1·55]), pancreatic cancer in Troms og Finnmark (1·22 [1·00–1·49]), and neonatal disorders in Troms og Finnmark (1·91 [1·59–2·31]).

The top ten causes of age-standardised YLDs in every county were low back pain, headache disorders, anxiety disorders, depressive disorders, falls, gynaecological diseases, endocrine disorders, diabetes, oral disorders, and age-related hearing loss. There were minor differences in the ranking of these between counties, with no significant differences between county rates and national rates for any of these causes in any county for males, females, or male and female sexes combined (appendix pp 16–18).

**Figure 4:**
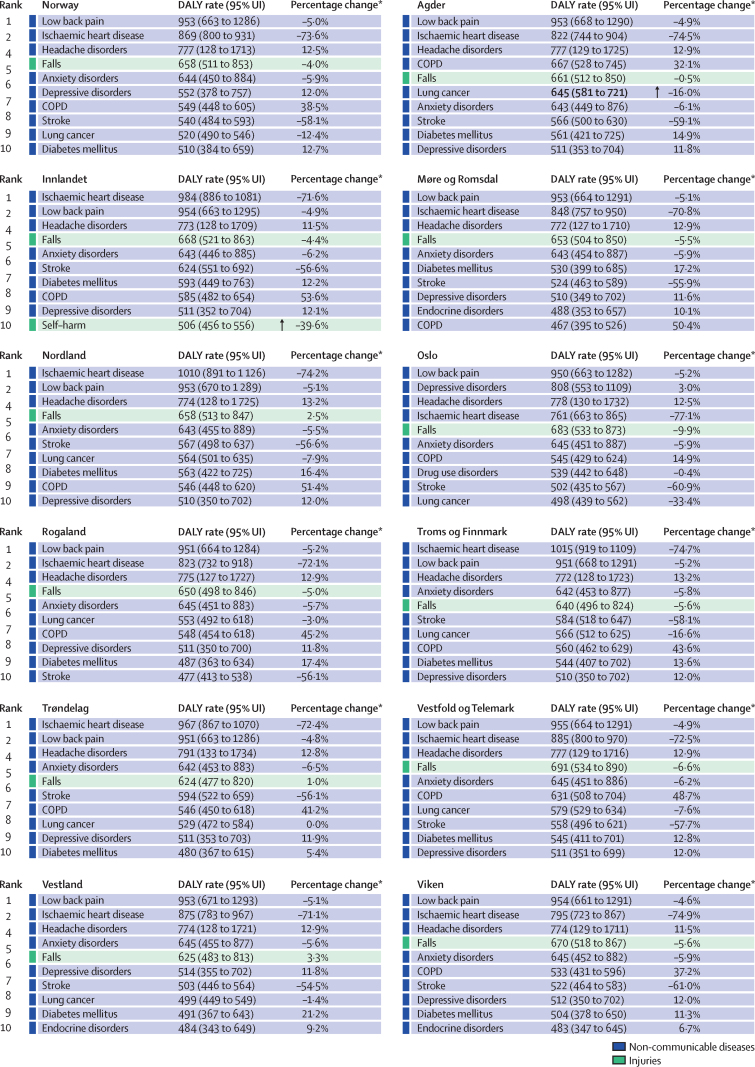
Age-standardised DALY rates per 100 000 inhabitants in each county for the leading ten level-3 causes in Norway and each Norwegian county, 2019 Data are the age-standardised DALY rates for male and female sexes combined. Data for causes of DALYs, years of life lost, and years lived with disability for males and females separately are shown in the appendix (pp 11–18). Bold rates indicate that the UI of the county estimate does not overlap the UI of the national estimate (upward arrow shows the county rate is higher than the national rate, downward arrow shows the county rate is less than the national rate). COPD=chronic obstructive pulmonary disease. DALY=disability-adjusted life-year. Dementia=Alzheimer's disease and other dementias. Endocrine disorders=endocrine, metabolic, blood, and immune disorders. Lung cancer=tracheal, bronchus, and lung cancer. MSK=musculoskeletal. UI=uncertainty interval. *Percentage change in age-standardised rate between 1990 and 2019.

The three main causes of age-standardised DALYs were low back pain, ischaemic heart disease, and headache disorders in every county, except for Oslo, where depressive disorders were ranked second (figure 4). Compared with the national rate, the rate of DALYs caused by lung cancer was higher in Agder (RR 1·24 [95% CI 1·11–1·39]), and the rate caused by self-harm was higher in Innlandet (1·19 [1·05–1·36]). Between 1990 and 2019, there was a substantial reduction in the rate of DALYs caused by ischaemic heart disease and stroke in all counties (figure 4). Over the same period, there was a substantial increase in the rate of DALYs caused by chronic obstructive pulmonary disease in most counties (figure 4), particularly among females (appendix p 12).

**Figure 5:**
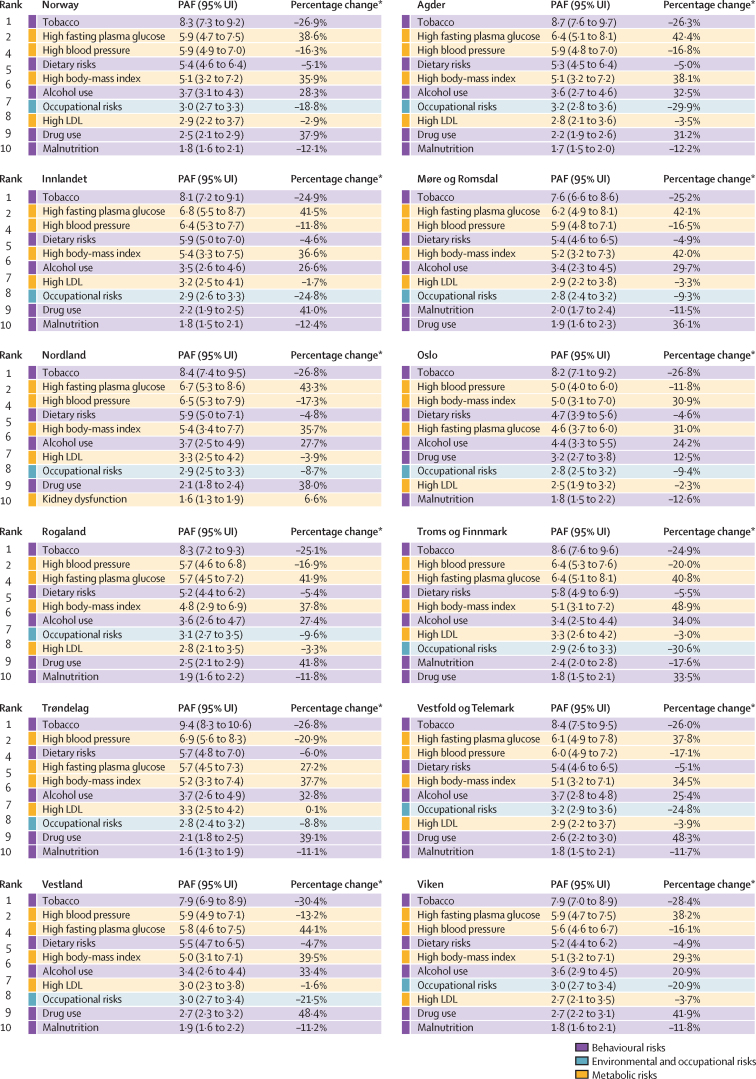
Leading ten level-2 risk factors for Norway and each Norwegian county by PAF for all-cause DALY rate per 100 000 inhabitants Data shown are age-standardised PAFs for males and females combined. Appendix p 19 shows 95% UIs for population attributable fractions. Appendix pp 19–20 show data for males and females separately. Appendix pp 21–22 show a heatmap and annualised rates of change for summary exposure value between 1990 and 2019. Colour code reflects level-1 risk categories. DALY=disability-adjusted life-year. PAF=population attributable fraction. UI=uncertainty interval. *Percentage change in summary exposure variable between 1990 and 2019.

The leading risk factors for DALYs were ranked similarly across all counties (figure 5). Tobacco and high blood pressure were among the top three risk factors in every county, despite exposure to these having fallen in all counties since 1990. By contrast, high fasting plasma glucose, high body-mass index, alcohol use, and drug use all increased substantially in the same period.

Exposure to the top ten level-2 risk factors for DALYS in Norway and in the 11 Norwegian counties between 1990 and 2019 are shown in the appendix (pp 21–22).

Between 1990 and 2019, there was a high degree of equality between counties for both life expectancy (Gini coefficient in 2019 was 0·004 for females [95% CI 0·003–0·006] and 0·006 for males [0·004–0·008]) and HALE (0·015 for females [0·009–0·023] and 0·012 for males [0·007–0·019]; appendix p 23). During the same period, there were also stable low levels of inequality between counties for all level-1 causes of DALYs. Inequality for communicable, maternal, neonatal, and nutritional diseases also decreased in the period (appendix p 23). Similarly, the inequality in exposure to level-1 risk factors was low.

In 2019, the level-2 causes of DALYs with noteworthy inequality between counties included HIV/AIDS and sexually transmitted infections (Gini coefficient 0·25 [95% CI 0·19–0·30]), maternal and neonatal disorders (0·11 [0·07–0·14]), and transport injuries (0·11 [0·08–0·13]; appendix pp 24–25).

## Discussion

Results from GBD 2019 showed only minor differences in life expectancy, HALE, and age-standardised all-cause YLLs, YLDs, and DALYs across the 11 Norwegian counties. Small regional differences in life expectancy in 1990 decreased even further over the past 30 years. Leading causes and risk factors for DALYs were also similar across counties in 2019. These findings contrast with subnational GBD results from the UK, in which YLL rates varied by up to 25% between UK countries and up to 30% between English regions.^16^, ^23^ The regional differences within Norway were also smaller than the differences found between the Nordic countries.^3^

The converging life expectancy between the Norwegian counties over the past 30 years follows a trend identified between the Nordic countries from 1990 to 2017,^3^ and in county-wise mortality in Norway since the 1980s.^20^ The convergence is most probably the consequence of differences in pace of change between the counties. Oslo, which had the lowest life expectancy among Norwegian counties in 1990, showed the greatest improvement, with an increase of 7·5 years over the 30-year period. By contrast, Møre og Romsdal and Vestland, which had the highest life expectancies in 1990, increased by only 5·4 years and 5·2 years, respectively, in the same period. The population of Oslo increased by almost 50% between 1990 and 2019 (from 460 000 to 680 000 inhabitants), mainly due to national and international migration. The proportion of international immigrants and their children increased from 12% to 33%. Thus, the gain in life expectancy in Oslo might partly be due to so-called healthy migrant and healthy worker effects.^39^, ^40^

The life expectancy decomposition model showed that decreased death rates for ischaemic heart disease and lung cancer were the primary reasons for the increasing life expectancy in all counties. Reduction in tobacco smoking is an important underlying explanation for the decreased death rates associated with cardiovascular diseases and lung cancer.^41^ Norwegian smoking habits have changed dramatically from 1990 to 2019, with a 53·5% reduction in the age-standardised prevalence rate of smoking.^41^ Smoking is also much less common in the younger generations.^42^ Among the 204 countries and territories included in GBD 2019, Norway had the largest decrease in smoking prevalence among young people between 1990 to 2019.^43^ The reduction in smoking prevalence is probably the result of a dedicated policy over almost 50 years.^44^ The first Tobacco Act came into force in 1975, and has been followed with increasingly strict regulations and taxation. However, sociocultural factors also have a role in smoking habits. Traditionally, there have been regional differences in smoking, with the highest rates in the north and the lowest rates in the west coast of mid-Norway, but this difference has also decreased over the past 30 years. Despite this change, tobacco is still a leading risk factor across all Norwegian counties, and the corresponding disease burden in the population is expected to remain high for several years, particularly in counties with an older population. Although smoking is much less common in younger generations, use of smoke-free tobacco (snus) has increased, particularly among younger women in Norway.^45^ Although GBD 2019 attributes a relatively small disease burden to snus, this trend is concerning.

High fasting plasma glucose, high body-mass index, alcohol use, and drug use increased substantially in all counties from 1990 to 2019, leading to health challenges associated with YLLs and YLDs. Further improvement in life expectancy might require reductions in these risk factors. The importance of reducing these risks, as well as dietary risks, are recognised in the Norwegian national NCD strategy, which is currently being updated and expanded.^46^ Illicit drug use continues to be a major challenge, particularly in counties in proximity to Oslo (Oslo, Viken, and Vestfold og Telemark), and in counties with larger cities, such as Bergen (Vestland) and Stavanger (Rogaland). Currently, there is a strong political debate on how to best handle the consistently high burden due to illicit drug use in Norway.

In the past 5 years, no notable changes in HALE have been found among females across all counties. This finding suggests that Norway, like many other countries, has been less successful in reducing non-fatal disease-associated health loss than fatal disease-associated health loss. There is no evidence of a reduction of health loss caused by musculoskeletal disorders, mental disorders, and headache disorders, all of which are more prevalent among women than men. Notably, there is a substantial gap between the size of the disease burden and evidence for effective prevention and treatment of musculoskeletal disorders, such as lower back and neck pain.^47^ Norway has several ongoing programmes directed towards mental health, including legal regulations to reduce bullying in kindergartens and schools, and low threshold access to mental health care; however, prevention of both mental and musculoskeletal disorders should remain a key priority for local public health initiatives.

Centralisation in Norway has primarily occurred within regions, and one potential explanation for the homogeneity in the burden of disease across counties might be the decentralised health-care system, with a strong emphasis on primary care. Access to health care is generally good throughout Norway, with a national estimated universal health care coverage index of 94 reported in GBD 2019.^29^ A regular general practitioner scheme was introduced in 2001, giving all citizens, permanent residents, refugees, and asylum seekers access to a designated general practitioner in their municipality. The density of doctors is among the highest in Europe, with 4·7 practising physicians per 1000 inhabitants.^48^ Despite the scattered population, Norway performs well in organising and delivering health care to its population, compared with other high-income countries. Compared with the USA, Nordic countries, including Norway, have lower health costs owing to their strong primary care systems, universal access to health care without financial barriers, and generally healthy lifestyle.^49^ Given that most of the follow-ups of patients with chronic diseases take place in the primary care system, the distribution of health workers across Norway might have been important in preventing geographical differences in health.^48^

Although the differences in disease burden between counties were minor, variation within counties might still be substantial. This subnational (ie, within-county and within-municipality) variation has been reported in England, where YLL rates vary by over 100% between local authority areas (analogous to Norwegian municipalities), compared with 30% between regions (analogous to Norwegian counties).^23^ Our Gini measure did not capture within-county inequalities, and rural–urban inequalities in morbidity and mortality seem to persist.^50^ Further, socioeconomic factors are important drivers of differences in health and mortality in Norway.^51^ For example, the richest 1% of men can expect to live 8·4 years longer than the poorest 1%, with a corresponding difference of 13·8 years among women.^52^ Lower occupational status is also associated with increased health problems, with a prevalence ratio of 2·0 for perceived poor health between men with the highest and lowest status.^53^ Large differences by educational attainment in Norway are found for health behaviours, particularly smoking, as well as for the prevalence and outcome of common causes of mortality, such as cardiovascular diseases, lung cancer, and chronic obstructive pulmonary disease.^54^ Additionally, large differences are evident in the socioeconomic profiles of certain municipalities within specific counties. The persistence of social inequalities in mortality in the Nordic welfare states has been characterised as a paradox,^55^ and this topic receives much political attention in Norway.

Availability of primary data is the most important limitation of the GBD project. For Norway, the availability of primary data is mainly a concern for non-fatal causes and risk factors, as GBD mortality estimates rely solely on high-quality data from the Norwegian Cause of Death Registry. Despite the redistribution of invalid death codes (garbage codes) done by GBD, GBD results on number of deaths by cause group is similar to the official Norwegian death statistics. Estimated life expectancy is also similar between GBD and national statistics.

The underlying Norwegian data sources for non-fatal causes of disease burden and risk factors are scarce and heterogeneous. Causes that appear in patient registries, such as cancers and cardiovascular diseases, are generally well covered across the counties. By contrast, data coverage is lower for causes such as mental and musculoskeletal disorders, for which help in the health services is not sought regularly. Although local health surveys are conducted regularly in Norway, there should be increased focus on collecting data for non-fatal causes, particularly for mental, neurological, and musculoskeletal disorders, as well as for important risk factors. Except for smoking, longitudinal data for health behaviours over the past 30 years are insufficient for most counties. GBD modelling ensures that estimates are produced for every cause and risk factor, even when local data are sparse, by borrowing data from similar regions, or global data. The validity of these results depends on the out-of-sample predictive validity of these models. This approach might conceal regional differences, which is probable given the county-wise similarities in HALE and non-fatal estimates. Thus, the minimal between-county differences in non-fatal estimates are more likely to be because of insufficient primary data rather than actual minimal differences in health, and this constitutes a major limitation of the present study.

The Norwegian regional policy seems to have been successful in making small, regional differences in health even smaller. However, much work remains in reducing socioeconomic inequalities in health. Policies that aim to reduce health differences in the population should focus not only on equal access to health services and healthy lifestyle options based on where people live in the country, but also on equal access regardless of neighbourhood, income, and education. A combination of universal and targeted prevention efforts (eg, towards immigrants) is recommended. Effective policies to meet such differences require a solid knowledge base. An expansion of GBD to include socioeconomic predictors of health would provide a more complete understanding of health and its determinants in the Norwegian population.

## Data sharing

Data are available at the Global Health Data Exchange GBD 2019 website.

## Declaration of interests

We declare no competing interests.
