# Supplementary material for: Changes in life expectancy and disease burden in Norway, 1990–2019: an analysis of the Global Burden of Disease Study 2019
Source: Lancet Public Health. 2022 Jun 29;7(7):e593–605. doi: 10.1016/S2468-2667(22)00092-5 (PMC9253891; doi:10.1016/S2468-2667(22)00092-5)
Supplement: Supplementary appendix [file mmc1.pdf]

THE LANCET
Public Health

Supplementary appendix

This appendix formed part of the original submission and has been peer reviewed. We post it as supplied by the authors.

Supplement to: Clarsen B, Nylenna M, Klitkou ST, et al. Changes in life expectancy and disease burden in Norway, 1990–2019: an analysis of the Global Burden of Disease Study 2019. *Lancet Public Health* 2022; **7:** e593–605.

Supplementary appendix

This appendix formed part of the original submission and has been peer reviewed.
We post it as supplied by the authors.

Supplement to: Changes in life expectancy and disease burden in Norway 1990-2019: an analysis of the Global Burden of Disease Study 2019

Table of content

Appendix tables 3

Table S1. Life expectancy at birth for males and females in Norway and the 11 Norwegian counties in 1990 and 2019. Values shown in parentheses indicate 95% uncertainty intervals. 3

Table S2. Healthy life expectancy (HALE) at birth for males and females in Norway and the 11 Norwegian counties in 1990 and 2019. Values shown in parentheses indicate 95% uncertainty intervals. 3

Table S3. All-cause YLL, YLD, and DALY rates per 100 000 inhabitants, 2019 [95% uncertainty interval]. Rates not standardised for age. Coloured cells indicate that the uncertainty interval of the county estimate does not overlap the uncertainty interval of the national estimate (red = county rate is above the national rate; blue = county rate is below the national rate). 4

Table S4. Age-standardised all-cause YLL, YLD, and DALY rates per 100 000 inhabitants, 2019 [95% uncertainty interval]. Coloured cells indicate that the uncertainty interval of the county estimate does not overlap the uncertainty interval of the national estimate (red = county rate is above the national rate; blue = county rate is below the national rate). 5

Table S5. Number of all-cause YLLs, YLDs and DALYs in Norway and each Norwegian county, 2019 [95% uncertainty interval] 6

Table S6. Percentage of DALYs attributed (PAF) to the top-10 level-2 risk factors in Norway and each Norwegian county in 2019. Age-standardised, both sexes combined. Values shown in parentheses indicate 95% uncertainty intervals. 7

Appendix figures 8

Figure S1. Map of Norway showing the 11 counties (black borders) and 356 municipalities (shaded areas). The background shade of each municipality reflects its degree of population centralisation, based on an index of all municipalities from the least-centralised (Utsira, 295) to the most centralised in Norway (Oslo, 1000).[1] 8

Figure S2. Change in life expectancy for males at birth in Norway and the 11 Norwegian counties between 1990 (black lines) and 2019 (red lines), decomposed into the contribution of GBD level 2 cause groups. Causes to the left of the 1990 life expectancy values reflect causes that contributed to reduced life expectancy between 1990 and 2019. Causes to the right of the 1990 life expectancy values reflect causes that contributed to increased life expectancy between 1990 and 2019. 9

Figure S3. Change in life expectancy for females at birth in Norway and the 11 Norwegian counties between 1990 (black lines) and 2019 (red lines), decomposed into the contribution of GBD level 2 cause groups. Causes to the left of the 1990 life expectancy values reflect causes that contributed to reduced life expectancy between 1990 and 2019. Causes to the right of the 1990 life expectancy values reflect causes that contributed to increased life expectancy between 1990 and 2019. 10

Figure S4. Age-standardised DALY rates per 100 000 male inhabitants in each county for the leading 10 level-3 causes in Norway and each Norwegian county, 2019. 11

Figure S5. Age-standardised DALY rates per 100 000 female inhabitants in each county for the leading 10 level-3 causes in Norway and each Norwegian county, 2019. 12

Figure S6. Age-standardised YLL rates per 100 000 female inhabitants in each county for the leading 10 level-3 causes in Norway and each Norwegian county, 2019. 13

Figure S7. Age-standardised YLL rates per 100 000 male inhabitants in each county for the leading 10 level-3 causes in Norway and each Norwegian county, 2019. 14

Figure S8. Age-standardised YLL rates per 100 000 female inhabitants in each county for the leading 10 level-3 causes in Norway and each Norwegian county, 2019. 15

Figure S9. Age-standardised YLD rates per 100 000 inhabitants in each county for the leading 10 level-3 causes in Norway and each Norwegian county, both sexes combined, 2019. 16

Figure S10. Age-standardised YLD rates per 100 000 male inhabitants in each county for the leading 10 level-3 causes in Norway and each Norwegian county, 2019. 17

Figure S11. Age-standardised YLD rates per 100 000 female inhabitants in each county for the leading 10 level-3 causes in Norway and each Norwegian county, 2019. 18

Figure S12. Leading 10 level-2 risk factors for Norway and each Norwegian county by population attributable fraction (PAF) for all-cause DALY rate per 100 000 male inhabitants. Age-standardised....... 19

Figure S13. Leading 10 level-2 risk factors for Norway and each Norwegian county by population attributable fraction (PAF) for all-cause DALY rate per 100 000 female inhabitants. Age-standardised. .. 20

Figure S14. Exposure to the top-10 level-2 risk factors for disability-adjusted life years in Norway and each Norwegian county. Colour scale reflects age-adjusted summary exposure variable (SEV) values and is anchored in the minimum SEV (black labels) and maximum SEV (white labels) in any county between 1990 and 2019. 21

Figure S14 (cont.). Exposure to the top-10 level-2 risk factors for disability-adjusted life years in Norway and each Norwegian county. Colour scale reflects age-adjusted summary exposure variable (SEV) values and is anchored in the minimum SEV (black labels) and maximum SEV (white labels) in any county between 1990 and 2019. 22

Figure S15a. Gini coefficient life expectancy and healthy life expectancy across all Norwegian counties, 1990-2019. Shaded areas represent 95% uncertainty bands. 23

Figure S15b. Gini coefficient for level-1 causes of DALYs and level-1 risk factors for DALYS across all Norwegian counties, 1990-2019. Shaded areas represent 95% uncertainty bands. 23

Figure S16. Gini coefficient and national and county-level estimates for level-2 cause group DALY rates in 2019. Shaded area represents the 95% uncertainty interval for the national YLL rate. GC = Gini coefficient and 95% uncertainty interval, DALY =Disability-Adjusted Life Year. Note: Gini coefficient estimates are higher when there is larger uncertainty in the underlying estimates.. 24

Figure S16 (cont.). Gini coefficient and national and county-level estimates for level-2 cause group DALY rates in 2019. Shaded area represents the 95% uncertainty interval for the national YLL rate. GC = Gini coefficient and 95% confidence interval, DALY =Disability-Adjusted Life Year. Note: Gini coefficient estimates are higher when there is larger uncertainty in the underlying estimates 25

Datasources non-fatal causes Norway GBD 2019 26

Appendix tables

Table S1. Life expectancy at birth for males and females in Norway and the 11 Norwegian counties in 1990 and 2019. Values shown in parentheses indicate 95% uncertainty intervals.

	Males		Females	
	1990	2019	1990	2019
Norway	73.6 [73.5, 73.8]	81.1 [80.9, 81.3]	80.2 [80.0, 80.3]	84.7 [84.4, 84.9]
Agder	72.9 [72.4, 73.3]	80.8 [79.9, 81.7]	79.9 [79.4, 80.4]	84.1 [83.0, 85.0]
Innlandet	73.6 [73.2, 74.1]	80.7 [79.8, 81.5]	79.9 [79.4, 80.3]	84.2 [83.3, 85.1]
Møre og Romsdal	75.1 [74.6, 75.6]	81.9 [80.7, 83.1]	81.0 [80.4, 81.5]	84.9 [83.9, 85.9]
Nordland	72.8 [72.3, 73.4]	80.2 [78.9, 81.4]	79.9 [79.3, 80.4]	84.4 [83.4, 85.5]
Oslo	71.9 [71.4, 72.4]	81.3 [80.0, 82.7]	79.0 [78.5, 79.4]	84.6 [83.7, 85.6]
Rogaland	74.8 [74.2, 75.3]	81.0 [79.9, 82.1]	80.6 [80.2, 81.1]	85.2 [84.3, 86.1]
Troms og Finnmark	71.9 [71.5, 72.4]	80.3 [79.4, 81.2]	79.9 [79.4, 80.5]	84.6 [83.6, 85.6]
Trøndelag	73.7 [73.2, 74.2]	80.7 [79.7, 81.8]	80.1 [79.6, 80.5]	84.0 [83.0, 85.1]
Vestfold og Telemark	73.6 [73.2, 74.1]	80.4 [79.6, 81.2]	80.0 [79.6, 80.5]	84.2 [83.4, 85.0]
Vestland	74.8 [74.4, 75.3]	81.2 [80.2, 82.2]	81.4 [81.0, 81.8]	85.4 [84.6, 86.2]
Viken	74.1 [73.8, 74.4]	81.9 [81.3, 82.6]	80.3 [79.9, 80.6]	84.9 [84.3, 85.5]

Table S2. Healthy life expectancy (HALE) at birth for males and females in Norway and the 11 Norwegian counties in 1990 and 2019. Values shown in parentheses indicate 95% uncertainty intervals.

	Males		Females	
	1990	2019	1990	2019
Norway	64.7 [62.1, 67.0]	70.5 [67.5, 73.2]	68.0 [64.4, 71.1]	71.1 [67.1, 74.6]
Agder	64.1 [61.7, 66.5]	70.3 [67.3, 73.1]	67.9 [64.4, 71.1]	70.7 [66.7, 74.3]
Innlandet	64.7 [62.1, 67.0]	70.1 [66.9, 72.9]	67.7 [64.1, 70.9]	70.7 [66.8, 74.4]
Møre og Romsdal	65.9 [63.1, 68.4]	71.1 [67.9, 74.0]	68.7 [65.1, 71.9]	71.4 [67.6, 75.0]
Nordland	64.2 [61.6, 66.5]	69.9 [66.9, 72.5]	67.8 [64.3, 70.9]	71.0 [67.0, 74.5]
Oslo	63.0 [60.5, 65.4]	70.6 [67.4, 73.6]	66.6 [62.9, 69.8]	70.7 [66.5, 74.3]
Rogaland	65.7 [62.9, 68.0]	70.5 [67.5, 73.3]	68.5 [64.8, 71.7]	71.6 [67.6, 75.2]
Troms og Finnmark	63.5 [60.9, 65.6]	70.0 [66.8, 72.7]	67.9 [64.3, 71.1]	71.2 [67.3, 74.7]
Trøndelag	64.8 [62.2, 67.2]	70.4 [67.3, 73.2]	68.0 [64.4, 71.2]	70.8 [66.9, 74.3]
Vestfold og Telemark	64.7 [62.1, 67.1]	69.9 [66.9, 72.7]	67.9 [64.2, 71.1]	70.8 [66.9, 74.3]
Vestland	65.8 [63.2, 68.2]	70.7 [67.7, 73.5]	69.2 [65.6, 72.4]	71.8 [67.9, 75.3]
Viken	65.1 [62.4, 67.4]	71.1 [67.9, 73.9]	68.1 [64.5, 71.2]	71.2 [67.2, 74.7]

Table S3. All-cause YLL, YLD, and DALY rates per 100 000 inhabitants, 2019 [95% uncertainty interval]. Rates not standardised for age. Coloured cells indicate that the uncertainty interval of the county estimate does not overlap the uncertainty interval of the national estimate (red = county rate is above the national rate; blue = county rate is below the national rate).

		Norway	Agder	Innlandet	Møre og Romsdal	Nordland	Oslo	Rogaland	Troms og Finnmark	Trøndelag	Vestfold og Telemark	Vestland	Viken
YLLs	Females	10 831<br>[10 586, 11 090]	11 508<br>[10 403, 12 855]	13 695<br>[12 417, 15 022]	11 478<br>[10 256, 12 840]	12 502<br>[11 099, 14 030]	8 873<br>[8 037, 9 793]	9 001<br>[8 146, 9 946]	11 220<br>[10 074, 12 494]	11 518<br>[10 264, 12 873]	12 569<br>[11 505, 13 803]	10 101<br>[9 230, 11 059]	10 534<br>[9 904, 11 284]
	Males	13 157<br>[12 842, 13 497]	13 613<br>[12 394, 14 970]	16 382<br>[14 924, 17 848]	13 318<br>[11 714, 15 053]	16 171<br>[14 277, 18 327]	10 405<br>[9 020, 11 920]	11 906<br>[10 628, 13 265]	14 872<br>[13 570, 16 274]	13 649<br>[12 243, 15 214]	15 564<br>[14 290, 16 948]	12 880<br>[11 651, 14 282]	12 175<br>[11 379, 13 026]
	Both sexes	12 004<br>[11 797, 12 228]	12 569<br>[11 777, 13 394]	15 041<br>[14 084, 15 970]	12 418<br>[11 427, 13 540]	14 366<br>[13 149, 15 705]	9 637<br>[8 809, 10 521]	10 477<br>[9 650, 11 362]	13 089<br>[12 196, 13 995]	12 601<br>[11 645, 13 568]	14 066<br>[13 236, 14 931]	11 512<br>[10 733, 12 362]	11 358<br>[10 854, 11 907]
YLDs	Females	15 117<br>[11 209, 19 640]	15 008<br>[11 111, 19 537]	16 271<br>[12 071, 21 015]	15 322<br>[11 335, 19 868]	15 721<br>[11 674, 20 263]	14 865<br>[11 011, 19 262]	14 250<br>[10 560, 18 518]	15 026<br>[11 079, 19 574]	14 963<br>[11 084, 19 417]	15 742<br>[11 677, 20 384]	14 770<br>[10 908, 19 259]	15 129<br>[11 234, 19 660]
	Males	12 002<br>[8 941, 15 500]	11 927<br>[8 891, 15 409]	13 024<br>[9 718, 16 768]	12 378<br>[9 197, 15 891]	12 597<br>[9 373, 16 225]	11 345<br>[8 436, 14 686]	11 362<br>[8 447, 14 630]	12 063<br>[8 967, 15 502]	11 905<br>[8 843, 15 326]	12 594<br>[9 366, 16 244]	11 801<br>[8 760, 15 220]	12 044<br>[8 987, 15 521]
	Both sexes	13 546<br>[10 025, 17 500]	13 455<br>[10 012, 17 408]	14 644<br>[10 848, 18 842]	13 818<br>[10 240, 17 895]	14 134<br>[10 492, 18 225]	13 108<br>[9 708, 16 951]	12 782<br>[9 500, 16 541]	13 510<br>[10 002, 17 454]	13 409<br>[9 930, 17 338]	14 168<br>[10 518, 18 298]	13 262<br>[9 787, 17 193]	13 581<br>[10 088, 17 516]
DALYs	Females	25 947<br>[21 982, 30 579]	26 516<br>[22 315, 31 182]	29 966<br>[25 278, 34 939]	26 800<br>[22 669, 31 501]	28 223<br>[24 017, 32 916]	23 738<br>[19 696, 28 453]	23 251<br>[19 330, 27 425]	26 247<br>[22 253, 30 764]	26 480<br>[22 417, 31 148]	28 312<br>[24 028, 33 148]	24 870<br>[20 956, 29 351]	25 663<br>[21 748, 30 384]
	Males	25 159<br>[22 074, 28 731]	25 540<br>[22 178, 29 163]	29 405<br>[25 848, 33 575]	25 696<br>[22 021, 29 880]	28 768<br>[25 015, 32 940]	21 749<br>[18 361, 25 302]	23 268<br>[20 019, 26 820]	26 935<br>[23 469, 30 884]	25 554<br>[22 055, 29 387]	28 158<br>[24 423, 32 077]	24 681<br>[21 307, 28 313]	24 220<br>[21 013, 27 806]
	Both sexes	25 550<br>[22 089, 29 492]	26 024<br>[22 320, 30 015]	29 685<br>[25 736, 34 261]	26 236<br>[22 447, 30 441]	28 500<br>[24 773, 32 809]	22 745<br>[19 117, 26 639]	23 260<br>[19 913, 27 115]	26 599<br>[23 058, 30 678]	26 010<br>[22 322, 29 957]	28 235<br>[24 460, 32 520]	24 774<br>[21 194, 28 639]	24 939<br>[21 502, 29 068]

Table S4. Age-standardised all-cause YLL, YLD, and DALY rates per 100 000 inhabitants, 2019 [95% uncertainty interval]. Coloured cells indicate that the uncertainty interval of the county estimate does not overlap the uncertainty interval of the national estimate (red = county rate is above the national rate; blue = county rate is below the national rate).

		Norway	Agder	Innlandet	Møre og Romsdal	Nordland	Oslo	Rogaland	Troms og Finnmark	Trøndelag	Vestfold og Telemark	Vestland	Viken
YLLs	Females	6 079<br>[5 923, 6 237]	6 525<br>[5 905, 7 288]	6 451<br>[5 874, 7 080]	5 813<br>[5 185, 6 508]	6 045<br>[5 348, 6 821]	6 132<br>[5 553, 6 771]	5 704<br>[5 162, 6 309]	6 521<br>[5 889, 7 228]	6 247<br>[5 565, 7 008]	6 433<br>[5 883, 7 045]	5 676<br>[5 176, 6 224]	5 935<br>[5 584, 6 357]
	Males	9 087<br>[8 865, 9 323]	9 413<br>[8 598, 10 330]	9 557<br>[8 723, 10 410]	8 554<br>[7 560, 9 644]	9 924<br>[8 770, 11 217]	8 861<br>[7 723, 10 126]	9 182<br>[8 241, 10 178]	10 313<br>[9 451, 11 260]	9 198<br>[8 261, 10 214]	9 807<br>[9 014, 10 654]	9 027<br>[8 169, 9 991]	8 353<br>[7 823, 8 905]
	Both sexes	7 536<br>[7 391, 7 691]	7 927<br>[7 421, 8 466]	7 956<br>[7 449, 8 469]	7 190<br>[6 621, 7 824]	7 961<br>[7 271, 8 733]	7 410<br>[6 776, 8 084]	7 393<br>[6 824, 8 027]	8 394<br>[7 801, 8 944]	7 687<br>[7 091, 8 289]	8 053<br>[7 568, 8 563]	7 307<br>[6 794, 7 860]	7 101<br>[6 781, 7 444]
YLDs	Females	12 086<br>[8 885, 15 765]	12 031<br>[8 852, 15 766]	12 089<br>[8 858, 15 696]	11 962<br>[8 790, 15 654]	12 017<br>[8 797, 15 660]	12 594<br>[9 290, 16 396]	11 965<br>[8 776, 15 620]	11 923<br>[8 707, 15 501]	11 983<br>[8 778, 15 628]	12 040<br>[8 829, 15 697]	11 920<br>[8 712, 15 646]	12 035<br>[8 822, 15 742]
	Males	9 814<br>[7 260, 12 677]	9 771<br>[7 248, 12 647]	9 837<br>[7 263, 12 693]	9 756<br>[7 216, 12 603]	9 751<br>[7 207, 12 640]	10 027<br>[7 433, 12 948]	9 752<br>[7 239, 12 573]	9 709<br>[7 172, 12 554]	9 740<br>[7 194, 12 533]	9 851<br>[7 280, 12 708]	9 754<br>[7 199, 12 628]	9 799<br>[7 253, 12 669]
	Both sexes	10 925<br>[8 055, 14 224]	10 879<br>[8 033, 14 080]	10 941<br>[8 062, 14 168]	10 814<br>[7 952, 14 032]	10 848<br>[7 989, 14 041]	11 303<br>[8 328, 14 645]	10 826<br>[7 984, 14 002]	10 771<br>[7 911, 13 996]	10 827<br>[7 947, 14 116]	10 930<br>[8 026, 14 182]	10 801<br>[7 940, 14 115]	10 900<br>[8 026, 14 160]
DALYs	Females	18 165<br>[14 875, 21 792]	18 556<br>[15 205, 22 491]	18 539<br>[15 109, 22 257]	17 775<br>[14 492, 21 490]	18 062<br>[14 793, 21 753]	18 726<br>[15 323, 22 753]	17 669<br>[14 359, 21 209]	18 445<br>[15 228, 22 170]	18 230<br>[15 009, 22 017]	18 473<br>[15 166, 22 273]	17 596<br>[14 405, 21 290]	17 969<br>[14 754, 21 672]
	Males	18 901<br>[16 338, 21 824]	19 185<br>[16 439, 22 138]	19 394<br>[16 742, 22 559]	18 310<br>[15 498, 21 585]	19 674<br>[16 885, 22 832]	18 888<br>[15 923, 22 000]	18 934<br>[16 245, 22 040]	20 022<br>[17 282, 23 174]	18 938<br>[16 188, 21 997]	19 658<br>[16 856, 22 708]	18 780<br>[16 059, 21 755]	18 152<br>[15 589, 21 136]
	Both sexes	18 461<br>[15 588, 21 713]	18 806<br>[15 771, 22 058]	18 897<br>[15 959, 22 206]	18 003<br>[15 016, 21 357]	18 808<br>[15 907, 22 121]	18 713<br>[15 591, 22 041]	18 219<br>[15 364, 21 498]	19 165<br>[16 268, 22 412]	18 514<br>[15 590, 21 799]	18 983<br>[16 002, 22 256]	18 108<br>[15 158, 21 360]	18 001<br>[15 155, 21 318]

Table S5. Number of all-cause YLLs, YLDs and DALYs in Norway and each Norwegian county, 2019 [95% uncertainty interval]

		Norway	Agder	Innlandet	Møre og Romsdal	Nordland	Oslo	Rogaland	Troms og Finnmark	Trøndelag	Vestfold og Telemark	Vestland	Viken
YLLs	Females	287 071 [280 586, 293 936]	17 398 [15 729, 19 435]	26 483 [24 011, 29 050]	15 116 [13 506, 16 909]	15 009 [13 325, 16 844]	30 543 [27 667, 33 711]	21 271 [19 250, 23 504]	13 418 [12 047, 14 941]	26 205 [23 351, 29 287]	26 655 [24 398, 29 271]	31 833 [29 089, 34 854]	63 139 [59 364, 67 633]
	Males	355 009 [346 510, 364 175]	20 918 [19 044, 23 003]	31 791 [28 963, 34 636]	18 307 [16 102, 20 691]	20 061 [17 711, 22 735]	35 690 [30 941, 40 887]	29 078 [25 958, 32 397]	18 641 [17 011, 20 399]	32 062 [28 760, 35 738]	32 990 [30 290, 35 924]	41 916 [37 918, 46 482]	73 555 [68 744, 78 696]
	Both sexes	642 081 [630 983, 654 069]	38 316 [35 901, 40 831]	58 274 [54 568, 61 874]	33 424 [30 757, 36 443]	35 070 [32 099, 38 338]	66 233 [60 541, 72 303]	50 349 [46 374, 54 601]	32 060 [29 873, 34 281]	58 267 [53 849, 62 742]	59 645 [56 121, 63 312]	73 750 [68 754, 79 192]	136 694 [130 625, 143 297]
YLDs	Females	400 675 [297 108, 520 564]	22 690 [16 798, 29 537]	31 464 [23 342, 40 638]	20 178 [14 927, 26 165]	18 875 [14 016, 24 327]	51 168 [37 903, 66 307]	33 676 [24 956, 43 761]	17 970 [13 249, 23 409]	34 042 [25 217, 44 177]	33 383 [24 761, 43 227]	46 548 [34 379, 60 698]	90 680 [67 334, 117 839]
	Males	323 858 [241 250, 418 230]	18 327 [13 662, 23 678]	25 274 [18 860, 32 542]	17 015 [12 643, 21 844]	15 627 [11 627, 20 128]	38 915 [28 936, 50 378]	27 749 [20 631, 35 731]	15 121 [11 240, 19 431]	27 965 [20 773, 36 002]	26 695 [19 853, 34 431]	38 407 [28 510, 49 533]	72 763 [54 295, 93 768]
	Both sexes	724 533 [536 216, 936 047]	41 018 [30 520, 53 067]	56 739 [42 030, 73 000]	37 193 [27 560, 48 165]	34 502 [25 612, 44 489]	90 084 [66 718, 116 494]	61 425 [45 652, 79 489]	33 091 [24 499, 42 753]	62 007 [45 918, 80 172]	60 078 [44 598, 77 587]	84 955 [62 695, 110 140]	163 443 [121 415, 210 801]
DALY	Females	687 747 [582 649, 810 526]	40 088 [33 738, 47 143]	57 948 [48 882, 67 563]	35 294 [29 854, 41 486]	33 884 [28 835, 39 518]	81 712 [67 800, 97 943]	54 947 [45 680, 64 811]	31 389 [26 612, 36 792]	60 247 [51 002, 70 866]	60 038 [50 953, 70 294]	78 381 [66 044, 92 502]	153 819 [130 355, 182 116]
	Males	678 867 [595 626, 775 234]	39 245 [34 079, 44 812]	57 065 [50 161, 65 158]	35 322 [30 271, 41 073]	35 688 [31 033, 40 863]	74 605 [62 983, 86 790]	56 827 [48 893, 65 503]	33 763 [29 418, 38 713]	60 028 [51 808, 69 031]	59 684 [51 768, 67 992]	80 323 [69 343, 92 144]	146 317 [126 944, 167 985]
	Both sexes	1 366 614 [1 181 515, 1 577 492]	79 334 [68 042, 91 499]	115 013 [99 713, 132 743]	70 616 [60 417, 81 933]	69 572 [60 473, 80 092]	156 317 [131 386, 183 076]	111 773 [95 691, 130 302]	65 151 [56 478, 75 143]	120 274 [103 219, 138 527]	119 722 [103 716, 137 893]	158 705 [135 773, 183 467]	300 137 [258 778, 349 831]

Table S6. Percentage of DALYs attributed (PAF) to the top-10 level-2 risk factors in Norway and each Norwegian county in 2019. Age-standardised, both sexes combined. Values shown in parentheses indicate 95% uncertainty intervals.

	Malnutrition	High LDL	Drug use	Occupational risks	Alcohol use	Dietary risks	High body-mass index	High blood pressure	High fasting plasma glucose	Tobacco
Norway	1.8 [1.6, 2.1]	2.9 [2.2, 3.7]	2.5 [2.1, 2.9]	3.0 [2.7, 3.3]	3.7 [3.1, 4.3]	5.4 [4.6, 6.4]	5.1 [3.2, 7.2]	5.9 [4.9, 7.0]	5.9 [4.7, 7.5]	8.3 [7.3, 9.2]
Agder	1.7 [1.5, 2.0]	2.8 [2.1, 3.6]	2.2 [1.9, 2.6]	3.2 [2.8, 3.6]	3.6 [2.7, 4.6]	5.3 [4.5, 6.4]	5.1 [3.2, 7.2]	5.9 [4.8, 7.0]	6.4 [5.1, 8.1]	8.7 [7.6, 9.7]
Innlandet	1.8 [1.5, 2.1]	3.2 [2.5, 4.1]	2.2 [1.9, 2.5]	2.9 [2.6, 3.3]	3.5 [2.6, 4.6]	5.9 [5.0, 7.0]	5.4 [3.3, 7.5]	6.4 [5.3, 7.7]	6.8 [5.5, 8.7]	8.1 [7.2, 9.1]
Møre og Romsdal	2.0 [1.7, 2.4]	2.9 [2.2, 3.8]	1.9 [1.6, 2.3]	2.8 [2.4, 3.2]	3.4 [2.3, 4.5]	5.4 [4.6, 6.5]	5.2 [3.2, 7.3]	5.9 [4.8, 7.1]	6.2 [4.9, 8.1]	7.6 [6.6, 8.6]
Nordland	1.6 [1.3, 1.9]	3.3 [2.5, 4.2]	2.1 [1.8, 2.4]	2.9 [2.5, 3.3]	3.7 [2.5, 4.9]	5.9 [5.0, 7.1]	5.4 [3.4, 7.7]	6.5 [5.3, 7.9]	6.7 [5.3, 8.6]	8.4 [7.4, 9.5]
Oslo	1.8 [1.5, 2.2]	2.5 [1.9, 3.2]	3.2 [2.7, 3.8]	2.8 [2.5, 3.2]	4.4 [3.3, 5.5]	4.7 [3.9, 5.6]	5.0 [3.1, 7.0]	5.0 [4.0, 6.0]	4.6 [3.7, 6.0]	8.2 [7.1, 9.2]
Rogaland	1.9 [1.6, 2.2]	2.8 [2.1, 3.5]	2.5 [2.1, 2.9]	3.1 [2.7, 3.5]	3.6 [2.6, 4.7]	5.2 [4.4, 6.2]	4.8 [2.9, 6.9]	5.7 [4.6, 6.8]	5.7 [4.5, 7.2]	8.3 [7.2, 9.3]
Troms og Finnmark	2.4 [2.0, 2.8]	3.3 [2.6, 4.2]	1.8 [1.5, 2.1]	2.9 [2.6, 3.3]	3.4 [2.5, 4.4]	5.8 [4.9, 6.9]	5.1 [3.1, 7.2]	6.4 [5.3, 7.6]	6.4 [5.1, 8.1]	8.6 [7.6, 9.6]
Trøndelag	1.6 [1.3, 1.9]	3.3 [2.5, 4.2]	2.1 [1.8, 2.5]	2.8 [2.4, 3.2]	3.7 [2.6, 4.9]	5.7 [4.8, 7.0]	5.2 [3.3, 7.4]	6.9 [5.6, 8.3]	5.7 [4.5, 7.3]	9.4 [8.3, 10.6]
Vestfold og Telemark	1.8 [1.5, 2.1]	2.9 [2.2, 3.7]	2.6 [2.2, 3.0]	3.2 [2.9, 3.6]	3.7 [2.8, 4.8]	5.4 [4.6, 6.5]	5.1 [3.2, 7.1]	6.0 [4.9, 7.2]	6.1 [4.9, 7.8]	8.4 [7.5, 9.5]
Vestland	1.9 [1.6, 2.2]	3.0 [2.3, 3.8]	2.7 [2.3, 3.2]	3.0 [2.7, 3.4]	3.4 [2.6, 4.4]	5.5 [4.7, 6.5]	5.0 [3.1, 7.1]	5.9 [4.9, 7.1]	5.8 [4.6, 7.5]	7.9 [6.9, 8.9]
Viken	1.8 [1.6, 2.1]	2.7 [2.1, 3.5]	2.7 [2.2, 3.1]	3.0 [2.7, 3.4]	3.6 [2.9, 4.5]	5.2 [4.4, 6.2]	5.1 [3.2, 7.1]	5.6 [4.6, 6.7]	5.9 [4.7, 7.5]	7.9 [7.0, 8.9]

Appendix figures

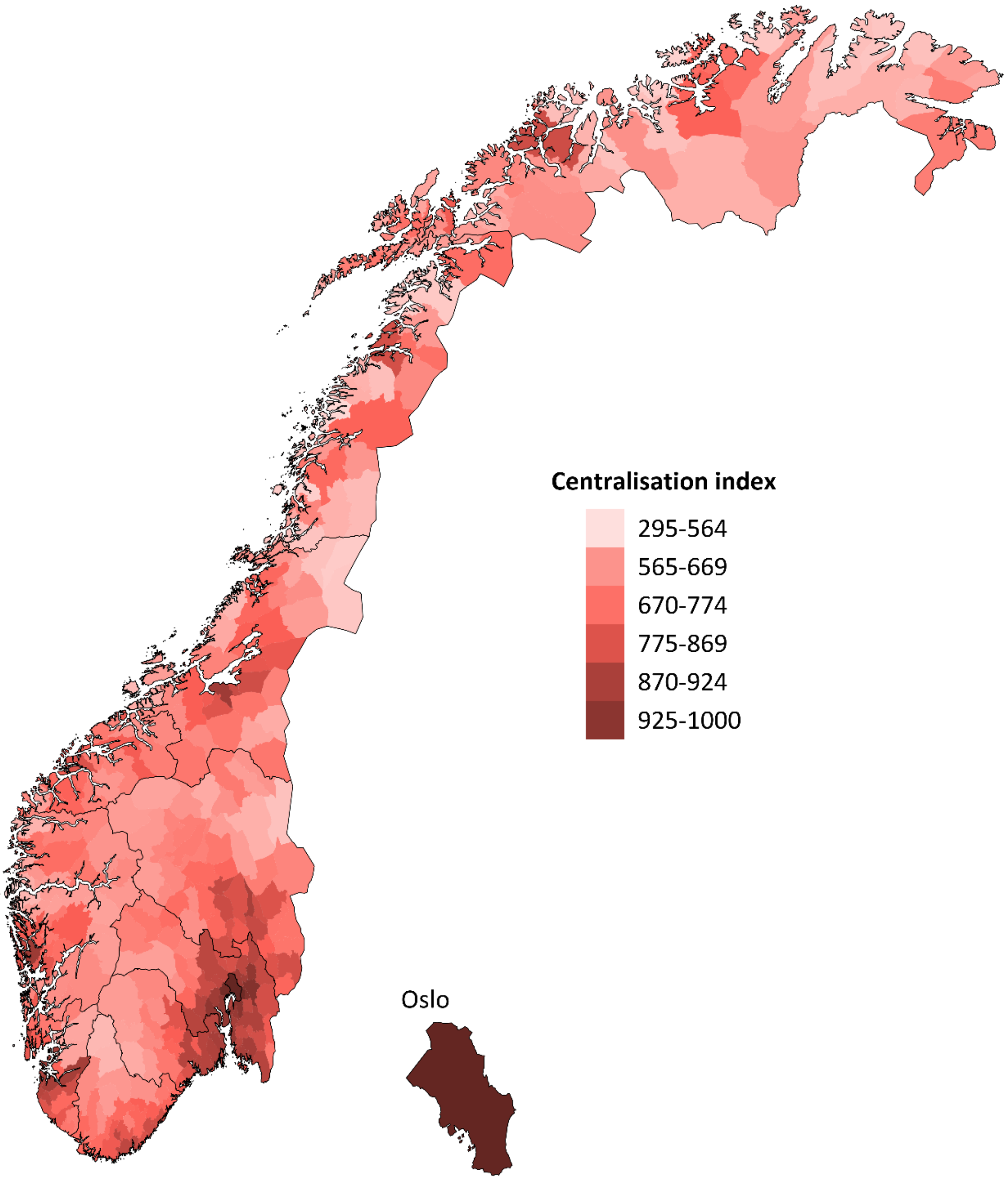

Figure S1. Map of Norway showing the 11 counties (black borders) and 356 municipalities (shaded areas). The background shade of each municipality reflects its degree of population centralisation, based on an index of all municipalities from the least-centralised (Utsira, 295) to the most centralised in Norway (Oslo, 1000).[1]

1. Høydal E. The centralization index. Update with 2020-municipalities [in Norwegian]. Oslo: Statistics Norway, 2020. https://www.ssb.no/befolkning/artikler-og-publikasjoner/_attachment/413602 (accessed June 6 2021).

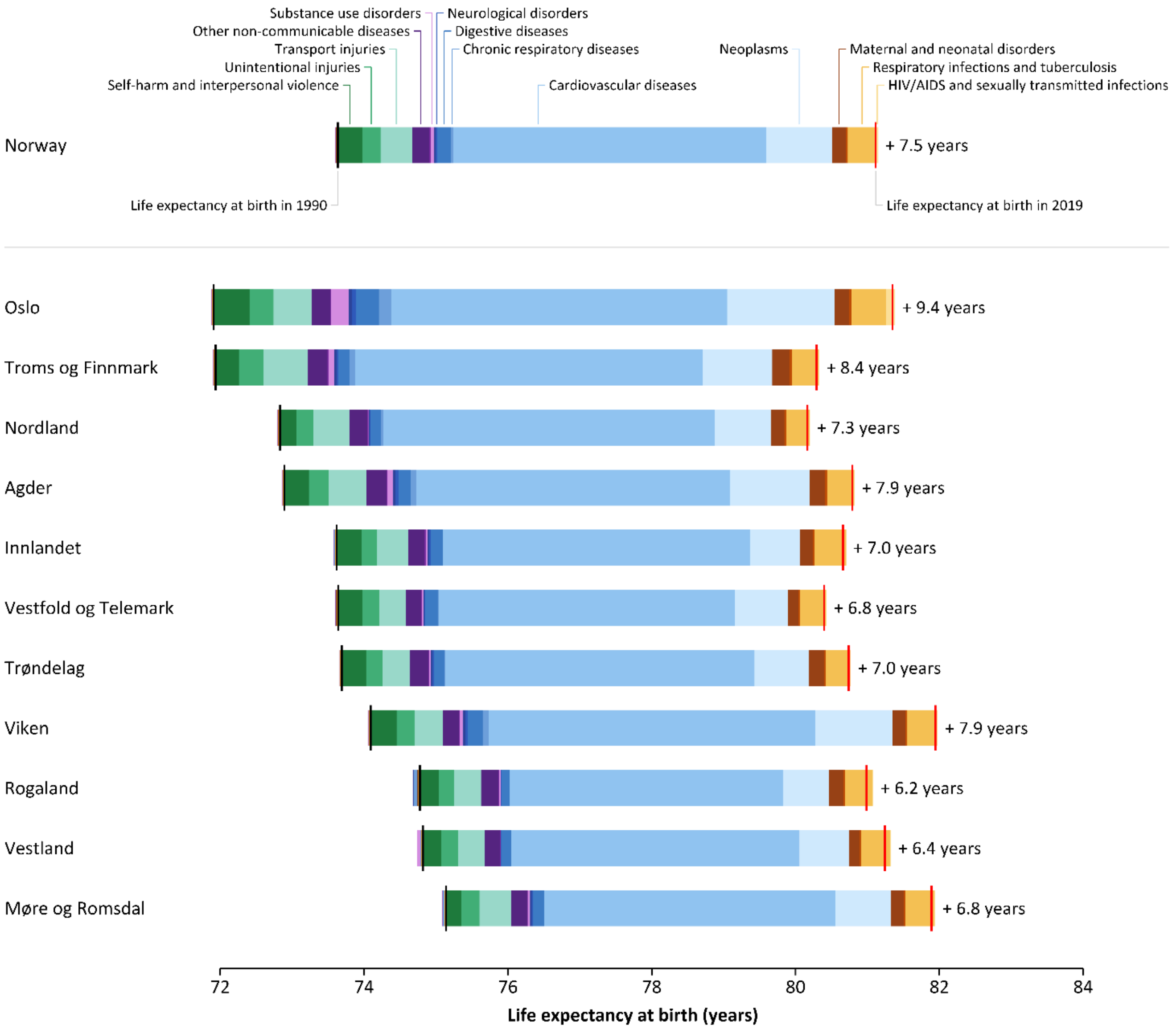

Figure S2. Change in life expectancy for males at birth in Norway and the 11 Norwegian counties between 1990 (black lines) and 2019 (red lines), decomposed into the contribution of GBD level 2 cause groups. Causes to the left of the 1990 life expectancy values reflect causes that contributed to reduced life expectancy between 1990 and 2019. Causes to the right of the 1990 life expectancy values reflect causes that contributed to increased life expectancy between 1990 and 2019.

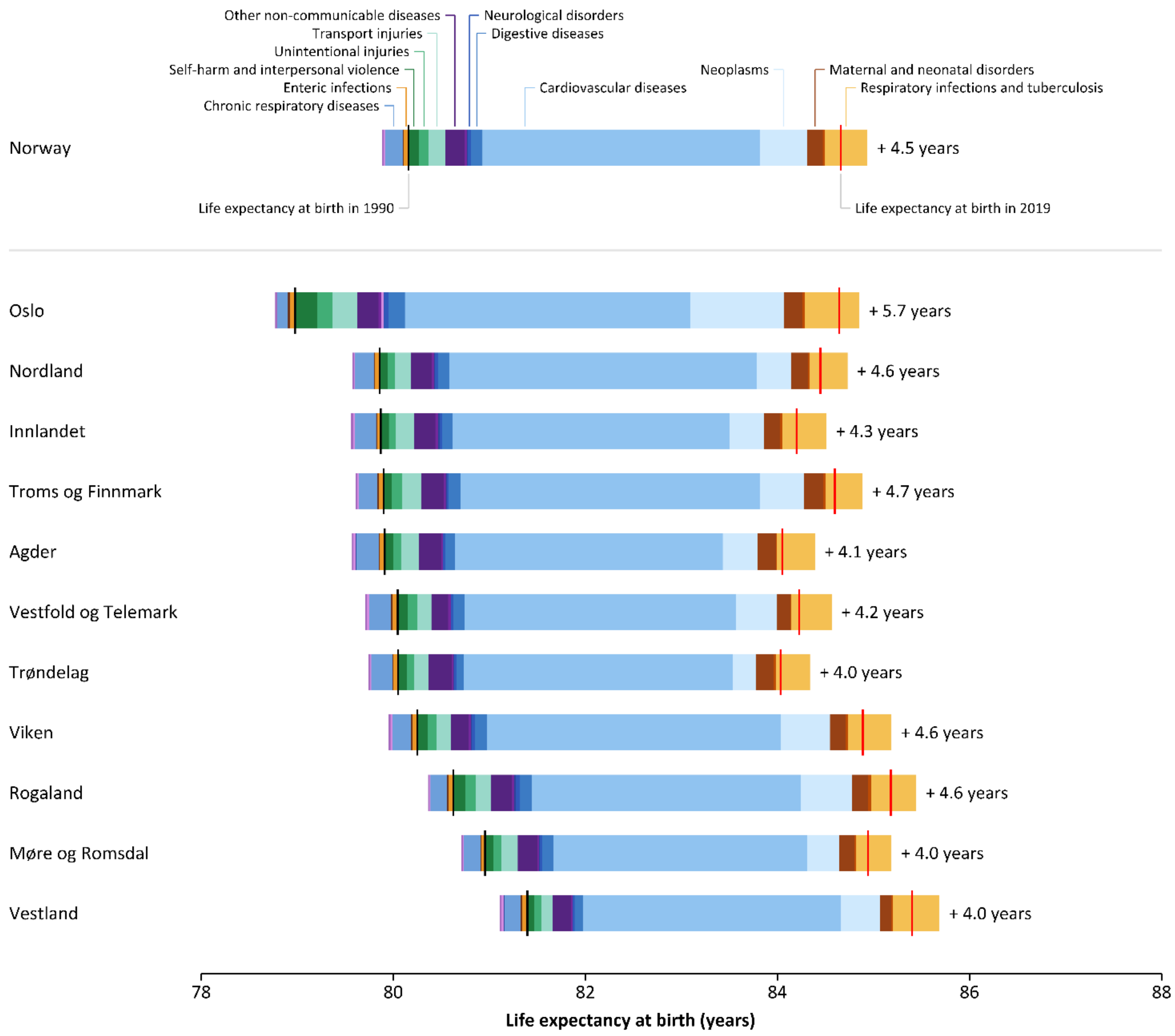

Figure S3. Change in life expectancy for females at birth in Norway and the 11 Norwegian counties between 1990 (black lines) and 2019 (red lines), decomposed into the contribution of GBD level 2 cause groups. Causes to the left of the 1990 life expectancy values reflect causes that contributed to reduced life expectancy between 1990 and 2019. Causes to the right of the 1990 life expectancy values reflect causes that contributed to increased life expectancy between 1990 and 2019.

Rank	Norway	DALY rate	[95% UI]		Change (%)
1	Ischemic heart disease	1 237	[1 159, 1 314]		-75.4
2	Low back pain	841	[583, 1 141]		-5.2
3	Falls	699	[552, 899]		-11.6
4	COPD	611	[551, 666]		11.4
5	Self-harm	595	[554, 636]		-46.2
6	Stroke	584	[528, 638]		-60.2
7	Lung cancer	580	[540, 616]		-36.4
8	Drug use disorders	577	[482, 686]		49.9
9	Diabetes mellitus	572	[434, 730]		9.0
10	Headache disorders	523	[88, 1 164]		12.9

Rank	Agder	DALY rate	[95% UI]		Change (%)
1	Ischemic heart disease	1 149	[1 027, 1 283]		-76.9
2	Low back pain	840	[587, 1 143]		-5.2
3	COPD	749	[654, 837]		4.8
4	Lung cancer	**705**	**[622, 798]**	↑	-42.2
5	Falls	700	[553, 891]		-10.6
6	Diabetes mellitus	636	[476, 808]		8.8
7	Self-harm	604	[527, 680]		-46.3
8	Stroke	600	[528, 673]		-62.9
9	Headache disorders	524	[86, 1 172]		13.1
10	Drug use disorders	519	[428, 626]		57.2

Rank	Innlandet	DALY rate	[95% UI]		Change (%)
1	Ischemic heart disease	1 391	[1 256, 1 540]		-73.1
2	Low back pain	842	[582, 1 141]		-5.3
3	Self-harm	**738**	**[656, 827]**	↑	-42.4
4	Falls	703	[558, 902]		-11.8
5	Stroke	675	[598, 755]		-58.4
6	Diabetes mellitus	660	[505, 836]		12.0
7	COPD	654	[578, 733]		26.7
8	Lung cancer	521	[460, 585]		-27.6
9	Headache disorders	508	[87, 1 127]		13.4
10	Prostate cancer	507	[382, 630]		-17.5

Rank	Møre og Romsdal	DALY rate	[95% UI]		Change (%)
1	Ischemic heart disease	1 162	[1 008, 1 327]		-73.4
2	Low back pain	843	[579, 1 146]		-5.2
3	Falls	708	[556, 916]		-12.7
4	Diabetes mellitus	594	[446, 762]		14.1
5	Self-harm	550	[475, 636]		-36.6
6	Stroke	549	[477, 628]		-57.8
7	COPD	529	[455, 611]		23.7
8	Headache disorders	524	[87, 1 167]		13.2
9	Lung cancer	523	[442, 619]		-28.3
10	Anxiety disorders	497	[347, 687]		-0.9

Rank	Nordland	DALY rate	[95% UI]		Change (%)
1	Ischemic heart disease	1 465	[1 279, 1 669]		-75.5
2	Low back pain	842	[587, 1 154]		-5.4
3	Self-harm	**756**	**[654, 871]**	↑	-31.5
4	Falls	714	[566, 904]		-2.7
5	Stroke	638	[557, 725]		-57.1
6	Diabetes mellitus	637	[483, 815]		14.9
7	Lung cancer	634	[541, 738]		-32.7
8	COPD	599	[512, 692]		22.9
9	Headache disorders	524	[85, 1 154]		13.0
10	Anxiety disorders	497	[352, 690]		-0.9

Rank	Oslo	DALY rate	[95% UI]		Change (%)
1	Ischemic heart disease	1 110	[950, 1 302]		-79.2
2	Low back pain	837	[587, 1 126]		-5.0
3	Drug use disorders	750	[604, 919]		-7.5
4	Falls	722	[565, 915]		-19.5
5	COPD	582	[496, 676]		-12.2
6	Depressive disorders	554	[382, 768]		1.6
7	Stroke	552	[472, 637]		-63.3
8	Self-harm	531	[446, 634]		-58.1
9	Headache disorders	522	[87, 1 164]		13.3
10	Lung cancer	507	[418, 604]		-55.2

Rank	Rogaland	DALY rate	[95% UI]		Change (%)
1	Ischemic heart disease	1 184	[1 038, 1 333]		-73.3
2	Low back pain	840	[584, 1 140]		-5.4
3	Falls	693	[539, 888]		-10.6
4	Lung cancer	675	[585, 779]		-23.7
5	COPD	639	[549, 729]		21.6
6	Drug use disorders	570	[466, 683]		62.9
7	Self-harm	568	[495, 645]		-41.4
8	Diabetes mellitus	549	[417, 708]		16.3
9	Headache disorders	524	[87, 1 156]		13.2
10	Stroke	522	[451, 595]		-56.4

Rank	Troms og Finnmark	DALY rate	[95% UI]		Change (%)
1	Ischemic heart disease	**1 479**	**[1 327, 1 638]**	↑	-76.4
2	Low back pain	841	[587, 1 138]		-5.6
3	Falls	688	[545, 882]		-13.7
4	Lung cancer	680	[602, 761]		-38.4
5	COPD	639	[557, 723]		13.1
6	Stroke	639	[570, 713]		-60.0
7	Self-harm	636	[561, 719]		-43.5
8	Diabetes mellitus	603	[453, 769]		11.4
9	Headache disorders	524	[87, 1 156]		13.2
10	Neonatal disorders	**500**	**[407, 603]**	↑	-65.6

Rank	Trøndelag	DALY rate	[95% UI]		Change (%)
1	Ischemic heart disease	1 374	[1 225, 1 548]		-74.2
2	Low back pain	840	[590, 1 137]		-5.1
3	Falls	664	[510, 864]		-7.0
4	Self-harm	632	[558, 720]		-46.3
5	Stroke	629	[550, 710]		-58.5
6	Lung cancer	593	[514, 679]		-27.3
7	COPD	578	[499, 661]		12.6
8	Diabetes mellitus	544	[415, 691]		4.1
9	Headache disorders	542	[94, 1 197]		13.4
10	Anxiety disorders	496	[349, 684]		-1.1

Rank	Vestfold og Telemark	DALY rate	[95% UI]		Change (%)
1	Ischemic heart disease	1 257	[1 135, 1 397]		-74.4
2	Low back pain	841	[583, 1 145]		-5.3
3	Falls	746	[587, 950]		-12.1
4	COPD	717	[630, 803]		22.2
5	Self-harm	708	[629, 793]		-42.0
6	Lung cancer	658	[581, 739]		-31.9
7	Diabetes mellitus	617	[468, 781]		10.7
8	Stroke	603	[537, 671]		-59.9
9	Drug use disorders	603	[500, 727]		73.3
10	Headache disorders	523	[88, 1 157]		13.1

Rank	Vestland	DALY rate	[95% UI]		Change (%)
1	Ischemic heart disease	1 265	[1 121, 1 423]		-73.0
2	Low back pain	841	[588, 1 148]		-5.3
3	Falls	663	[518, 860]		-4.6
4	Drug use disorders	644	[536, 778]		80.3
5	Lung cancer	603	[526, 689]		-26.3
6	COPD	578	[500, 656]		12.1
7	Stroke	556	[489, 626]		-56.7
8	Self-harm	550	[481, 623]		-41.2
9	Diabetes mellitus	546	[413, 705]		17.5
10	Headache disorders	524	[88, 1 153]		13.0

Rank	Viken	DALY rate	[95% UI]		Change (%)
1	Ischemic heart disease	1 118	[1 022, 1 219]		-76.6
2	Low back pain	841	[584, 1 141]		-4.8
3	Falls	702	[548, 901]		-13.9
4	Drug use disorders	597	[492, 719]		49.8
5	COPD	567	[499, 630]		6.5
6	Diabetes mellitus	561	[424, 717]		6.8
7	Stroke	555	[495, 616]		-63.3
8	Self-harm	545	[488, 604]		-49.8
9	Headache disorders	520	[88, 1 135]		11.8
10	Lung cancer	505	[454, 560]		-43.6

Communicable, maternal, neonatal, and nutritional diseases
Non-communicable diseases
Injuries

Figure S4. Age-standardised DALY rates per 100 000 male inhabitants in each county for the leading 10 level-3 causes in Norway and each Norwegian county, 2019. Bold rates indicate that the uncertainty interval of the county estimate does not overlap the uncertainty interval of the national estimate (upward arrow = county rate is above the national rate, downward arrow = county rate is below the national rate). Lung cancer = Tracheal, bronchus, and lung cancer, Dementia = Alzheimer's disease and other dementias, COPD = Chronic obstructive pulmonary disease, MSK = Musculoskeletal, Endocrine disorders = Endocrine, metabolic, blood, and immune disorders, DALY = Disability-Adjusted Life Years.

Rank	**Norway**	DALY rate	[95% UI]	Change (%)
1	Low back pain	1 065	[744, 1 434]	-4.0
2	Headache disorders	1 043	[168, 2 343]	12.9
3	Gynecological diseases	965	[654, 1 352]	-1.3
4	Anxiety disorders	798	[559, 1 094]	-8.6
5	Depressive disorders	671	[459, 918]	14.3
6	Falls	610	[462, 793]	6.7
7	Endocrine disorders	550	[387, 745]	5.0
8	Ischemic heart disease	524	[460, 578]	-70.9
9	COPD	502	[331, 576]	72.7
10	Stroke	499	[439, 555]	-56.5

Rank	**Agder**	DALY rate	[95% UI]	Change (%)
1	Low back pain	1 067	[742, 1 440]	-3.9
2	Headache disorders	1 043	[172, 2 344]	13.4
3	Gynecological diseases	970	[653, 1 351]	-1.4
4	Anxiety disorders	796	[554, 1 090]	-9.0
5	Falls	612	[464, 798]	13.1
6	Depressive disorders	606	[418, 838]	13.7
7	COPD	602	[365, 716]	69.6
8	Lung cancer	**593**	**[512, 683]** ↑	53.6
9	Endocrine disorders	549	[383, 750]	5.5
10	Stroke	532	[456, 605]	-55.9

Rank	**Innlandet**	DALY rate	[95% UI]	Change (%)
1	Low back pain	1 066	[744, 1 432]	-4.1
2	Headache disorders	1 051	[171, 2 380]	11.1
3	Gynecological diseases	967	[654, 1 348]	-1.4
4	Anxiety disorders	797	[552, 1 091]	-8.9
5	Falls	623	[474, 814]	5.4
6	Depressive disorders	606	[416, 840]	13.9
7	Ischemic heart disease	606	[513, 692]	-68.6
8	Stroke	577	[499, 651]	-54.9
9	Endocrine disorders	551	[387, 743]	5.6
10	COPD	533	[347, 631]	93.7

Rank	**Møre og Romsdal**	DALY rate	[95% UI]	Change (%)
1	Low back pain	1 067	[753, 1 445]	-4.2
2	Headache disorders	1 043	[168, 2 345]	13.4
3	Gynecological diseases	967	[658, 1 343]	-1.3
4	Anxiety disorders	800	[558, 1 097]	-8.5
5	Depressive disorders	606	[417, 831]	13.4
6	Falls	590	[445, 773]	4.8
7	Endocrine disorders	553	[387, 763]	6.5
8	Ischemic heart disease	535	[459, 619]	-67.4
9	Stroke	495	[426, 568]	-54.8
10	Diabetes mellitus	464	[342, 613]	19.6

Rank	**Nordland**	DALY rate	[95% UI]	Change (%)
1	Low back pain	1 067	[747, 1 440]	-4.2
2	Headache disorders	1 043	[169, 2 363]	13.5
3	Gynecological diseases	972	[661, 1 357]	-1.1
4	Anxiety disorders	799	[559, 1 103]	-8.2
5	Depressive disorders	607	[417, 841]	13.9
6	Falls	595	[452, 777]	10.4
7	Ischemic heart disease	570	[483, 652]	-72.7
8	Endocrine disorders	553	[388, 749]	6.9
9	COPD	509	[335, 605]	87.9
10	Lung cancer	501	[425, 589]	53.5

Rank	**Oslo**	DALY rate	[95% UI]	Change (%)
1	Depressive disorders	1 062	[724, 1 462]	5.4
2	Low back pain	1 059	[741, 1 431]	-3.9
3	Headache disorders	1 037	[170, 2 344]	13.5
4	Gynecological diseases	952	[650, 1 345]	-1.3
5	Anxiety disorders	795	[554, 1 090]	-8.2
6	Falls	635	[483, 825]	3.2
7	Endocrine disorders	551	[390, 746]	2.5
8	COPD	521	[335, 621]	40.6
9	Lung cancer	496	[422, 579]	4.9
10	Asthma	466	[300, 700]	-38.2

Rank	**Rogaland**	DALY rate	[95% UI]	Change (%)
1	Low back pain	1 065	[744, 1 429]	-4.2
2	Headache disorders	1 043	[168, 2 345]	13.5
3	Gynecological diseases	960	[651, 1 342]	-1.3
4	Anxiety disorders	800	[557, 1 087]	-8.3
5	Depressive disorders	606	[413, 838]	13.7
6	Falls	599	[450, 782]	3.5
7	Endocrine disorders	538	[372, 742]	4.1
8	Ischemic heart disease	481	[401, 558]	-71.4
9	COPD	479	[321, 574]	74.9
10	Lung cancer	442	[377, 511]	46.5

Rank	**Troms og Finnmark**	DALY rate	[95% UI]	Change (%)
1	Low back pain	1 067	[752, 1 448]	-4.3
2	Headache disorders	1 043	[170, 2 332]	13.5
3	Gynecological diseases	949	[643, 1 325]	-2.0
4	Anxiety disorders	799	[559, 1 096]	-8.6
5	Depressive disorders	607	[414, 840]	13.9
6	Falls	582	[441, 759]	6.1
7	Ischemic heart disease	566	[485, 652]	-71.3
8	Endocrine disorders	546	[388, 740]	3.7
9	Stroke	530	[456, 606]	-56.8
10	COPD	497	[322, 589]	89.2

Rank	**Trøndelag**	DALY rate	[95% UI]	Change (%)
1	Low back pain	1 065	[749, 1 441]	-3.8
2	Headache disorders	1 057	[176, 2 353]	13.3
3	Gynecological diseases	973	[655, 1 361]	-0.8
4	Anxiety disorders	797	[556, 1 093]	-9.3
5	Depressive disorders	607	[416, 835]	14.0
6	Ischemic heart disease	586	[497, 673]	-69.6
7	Falls	577	[435, 765]	11.8
8	Stroke	559	[483, 632]	-54.3
9	Endocrine disorders	548	[380, 750]	6.0
10	COPD	528	[352, 634]	76.8

Rank	**Vestfold og Telemark**	DALY rate	[95% UI]	Change (%)
1	Low back pain	1 067	[743, 1 439]	-4.0
2	Headache disorders	1 042	[168, 2 341]	13.4
3	Gynecological diseases	967	[652, 1 354]	-1.3
4	Anxiety disorders	798	[555, 1 100]	-8.8
5	Falls	630	[481, 817]	1.7
6	Depressive disorders	606	[416, 833]	14.0
7	COPD	564	[352, 663]	81.3
8	Endocrine disorders	559	[395, 756]	6.3
9	Ischemic heart disease	545	[467, 622]	-69.6
10	Stroke	516	[448, 586]	-56.0

Rank	**Vestland**	DALY rate	[95% UI]	Change (%)
1	Low back pain	1 067	[751, 1 447]	-4.0
2	Headache disorders	1 042	[171, 2 337]	13.3
3	Gynecological diseases	971	[659, 1 369]	-0.9
4	Anxiety disorders	800	[561, 1 084]	-8.3
5	Depressive disorders	611	[418, 838]	13.9
6	Falls	578	[436, 757]	13.7
7	Endocrine disorders	552	[384, 759]	6.4
8	Ischemic heart disease	505	[426, 579]	-68.9
9	Stroke	454	[391, 516]	-53.1
10	Diabetes mellitus	435	[319, 579]	24.7

Rank	**Viken**	DALY rate	[95% UI]	Change (%)
1	Low back pain	1 066	[747, 1 437]	-3.8
2	Headache disorders	1 039	[168, 2 350]	12.0
3	Gynecological diseases	966	[655, 1 343]	-1.2
4	Anxiety disorders	798	[556, 1 092]	-8.4
5	Falls	630	[478, 819]	5.7
6	Depressive disorders	607	[417, 833]	13.9
7	Endocrine disorders	552	[389, 748]	4.3
8	COPD	509	[332, 596]	77.9
9	Ischemic heart disease	497	[433, 561]	-72.0
10	Stroke	490	[425, 554]	-59.1

Non-communicable diseases
Injuries

Figure S5. Age-standardised DALY rates per 100 000 female inhabitants in each county for the leading 10 level-3 causes in Norway and each Norwegian county, 2019. Bold rates indicate that the uncertainty interval of the county estimate does not overlap the uncertainty interval of the national estimate (upward arrow = county rate is above the national rate, downward arrow = county rate is below the national rate). Lung cancer = Tracheal, bronchus, and lung cancer, Dementia = Alzheimer's disease and other dementias, COPD = Chronic obstructive pulmonary disease, MSK = Musculoskeletal, Endocrine disorders = Endocrine, metabolic, blood, and immune disorders, DALY = Disability-Adjusted Life Years.

Rank	Norway	YLL rate	[95% UI]		Change (%)
1	Ischemic heart disease	811	[745, 871]		-74.8
2	Lung cancer	511	[482, 537]		-12.7
3	Self-harm	416	[389, 443]		-43.8
4	Stroke	378	[341, 411]		-66.2
5	Colon and rectum cancer	367	[341, 390]		-20.0
6	COPD	350	[268, 381]		48.6
7	Dementia	209	[51, 542]		-9.2
8	Drug use disorders	196	[179, 226]		70.0
9	Breast cancer	192	[178, 207]		-45.0
10	Congenital birth defects	185	[153, 277]		-64.3

Rank	Agder	YLL rate	[95% UI]		Change (%)
1	Ischemic heart disease	764	[690, 846]		-75.8
2	Lung cancer	**635**	**[571, 711]**	↑	-16.4
3	COPD	448	[326, 510]		38.4
4	Self-harm	430	[383, 474]		-42.4
5	Stroke	401	[355, 450]		-66.9
6	Colon and rectum cancer	366	[328, 406]		-17.6
7	Breast cancer	210	[181, 242]		-40.5
8	Dementia	207	[50, 556]		-8.9
9	Lower respiratory infections	202	[176, 225]		-60.6
10	Pancreatic cancer	185	[165, 206]		2.3

Rank	Innlandet	YLL rate	[95% UI]		Change (%)
1	Ischemic heart disease	926	[832, 1 021]		-72.7
2	Self-harm	**498**	**[449, 547]**	↑	-39.7
3	Lung cancer	472	[426, 521]		-0.9
4	Stroke	455	[404, 508]		-63.9
5	COPD	380	[288, 431]		70.3
6	Colon and rectum cancer	337	[302, 372]		-15.0
7	Congenital birth defects	207	[170, 291]		-63.9
8	Dementia	205	[50, 549]		-8.3
9	Prostate cancer	199	[150, 248]		-20.9
10	Lower respiratory infections	197	[172, 218]		-64.3

Rank	Møre og Romsdal	YLL rate	[95% UI]		Change (%)
1	Ischemic heart disease	789	[701, 891]		-72.2
2	Lung cancer	443	[392, 499]		-3.4
3	Self-harm	369	[328, 418]		-36.9
4	Colon and rectum cancer	365	[321, 411]		-15.5
5	Stroke	362	[313, 412]		-64.6
6	COPD	282	[223, 328]		68.4
7	Congenital birth defects	209	[170, 305]		-60.1
8	Dementia	206	[51, 569]		-9.8
9	Neonatal disorders	197	[144, 246]		-66.2
10	Road injuries	**190**	**[166, 216]**	↑	-66.6

Rank	Nordland	YLL rate	[95% UI]		Change (%)
1	Ischemic heart disease	949	[830, 1 065]		-75.2
2	Lung cancer	555	[492, 627]		-8.2
3	Self-harm	486	[429, 551]		-31.8
4	Stroke	402	[347, 458]		-64.7
5	Colon and rectum cancer	377	[333, 426]		-12.2
6	COPD	345	[257, 401]		66.4
7	Dementia	208	[51, 554]		-8.0
8	Pancreatic cancer	199	[172, 229]		0.6
9	Prostate cancer	191	[152, 267]		-15.0
10	Road injuries	185	[161, 214]		-65.1

Rank	Oslo	YLL rate	[95% UI]		Change (%)
1	Ischemic heart disease	707	[608, 809]		-78.4
2	Lung cancer	491	[433, 555]		-33.7
3	Self-harm	403	[351, 461]		-55.6
4	Stroke	348	[295, 398]		-68.9
5	Colon and rectum cancer	345	[299, 398]		-36.1
6	COPD	345	[239, 412]		15.4
7	Drug use disorders	**276**	**[230, 346]**	↑	-8.8
8	Dementia	240	[61, 624]		-7.5
9	Breast cancer	210	[179, 243]		-53.9
10	Lower respiratory infections	186	[157, 216]		-68.2

Rank	Rogaland	YLL rate	[95% UI]		Change (%)
1	Ischemic heart disease	765	[674, 857]		-73.5
2	Lung cancer	544	[485, 609]		-3.4
3	Colon and rectum cancer	401	[358, 446]		-17.1
4	Self-harm	387	[344, 432]		-42.1
5	COPD	348	[269, 407]		60.9
6	Stroke	322	[279, 366]		-65.2
7	Dementia	207	[52, 553]		-10.1
8	Breast cancer	193	[165, 225]		-46.9
9	Prostate cancer	193	[150, 262]		-13.9
10	Drug use disorders	190	[161, 229]		85.9

Rank	Troms og Finnmark	YLL rate	[95% UI]		Change (%)
1	Ischemic heart disease	958	[863, 1 056]		-75.7
2	Lung cancer	558	[504, 617]		-17.0
3	Self-harm	433	[389, 480]		-41.0
4	Stroke	421	[370, 471]		-65.5
5	COPD	361	[277, 413]		54.3
6	Colon and rectum cancer	343	[307, 376]		-15.0
7	Neonatal disorders	**322**	**[255, 399]**	↑	-72.8
8	Congenital birth defects	296	[238, 389]		-65.8
9	Pancreatic cancer	**219**	**[195, 244]**	↑	-0.4
10	Dementia	205	[50, 546]		-7.1

Rank	Trøndelag	YLL rate	[95% UI]		Change (%)
1	Ischemic heart disease	906	[812, 1 006]		-73.5
2	Lung cancer	521	[465, 576]		-0.4
3	Self-harm	428	[384, 478]		-43.5
4	Stroke	426	[371, 480]		-63.8
5	Colon and rectum cancer	389	[346, 433]		-16.0
6	COPD	347	[259, 407]		54.2
7	Dementia	204	[49, 543]		-8.6
8	Pancreatic cancer	201	[176, 227]		3.2
9	Breast cancer	198	[170, 230]		-42.3
10	Prostate cancer	195	[157, 270]		-14.5

Rank	Vestfold og Telemark	YLL rate	[95% UI]		Change (%)
1	Ischemic heart disease	828	[741, 912]		-73.7
2	Lung cancer	570	[520, 624]		-8.0
3	Self-harm	**496**	**[447, 550]**	↑	-39.2
4	COPD	416	[305, 471]		64.2
5	Stroke	394	[349, 442]		-65.6
6	Colon and rectum cancer	373	[336, 410]		-16.6
7	Drug use disorders	206	[180, 243]		109.5
8	Dementia	206	[51, 533]		-9.2
9	Breast cancer	199	[175, 228]		-41.9
10	Congenital birth defects	195	[159, 287]		-59.7

Rank	Vestland	YLL rate	[95% UI]		Change (%)
1	Ischemic heart disease	815	[728, 906]		-72.5
2	Lung cancer	492	[442, 541]		-1.8
3	Colon and rectum cancer	410	[366, 456]		-17.0
4	Self-harm	391	[350, 434]		-37.8
5	Stroke	343	[298, 385]		-63.7
6	COPD	308	[250, 351]		45.3
7	Drug use disorders	222	[189, 270]		118.9
8	Dementia	206	[51, 556]		-8.6
9	Congenital birth defects	199	[164, 291]		-60.7
10	Prostate cancer	188	[150, 261]		-10.5

Rank	Viken	YLL rate	[95% UI]		Change (%)
1	Ischemic heart disease	738	[674, 809]		-76.1
2	Lung cancer	475	[439, 512]		-18.6
3	Self-harm	387	[354, 424]		-46.4
4	Stroke	359	[324, 398]		-69.1
5	Colon and rectum cancer	350	[320, 381]		-22.1
6	COPD	330	[244, 368]		46.3
7	Dementia	204	[51, 525]		-8.2
8	Drug use disorders	198	[174, 237]		70.8
9	Breast cancer	190	[171, 213]		-45.5
10	Congenital birth defects	174	[140, 274]		-64.3

- Communicable, maternal, neonatal, and nutritional diseases
- Non-communicable diseases
- Injuries

Figure S6. Age-standardised YLL rates per 100 000 female inhabitants in each county for the leading 10 level-3 causes in Norway and each Norwegian county, 2019. Bold rates indicate that the uncertainty interval of the county estimate does not overlap the uncertainty interval of the national estimate (upward arrow = county rate is above the national rate, downward arrow = county rate is below the national rate). Lung cancer = Tracheal, bronchus, and lung cancer, Dementia = Alzheimer's disease and other dementias, COPD = Chronic obstructive pulmonary disease, MSK = Musculoskeletal, Endocrine disorders = Endocrine, metabolic, blood, and immune disorders, YLL = Years of Lost Life.

Rank	Norway	YLL rate	[95% UI]		Change (%)
1	Ischemic heart disease	1 154	[1 084, 1 231]		-76.6
2	Self-harm	588	[546, 629]		-46.3
3	Lung cancer	571	[532, 606]		-36.7
4	Stroke	421	[388, 452]		-67.7
5	Colon and rectum cancer	413	[381, 440]		-23.7
6	COPD	408	[375, 444]		13.1
7	Prostate cancer	408	[332, 539]		-23.5
8	Drug use disorders	299	[271, 359]		66.5
9	Road injuries	225	[213, 238]		-71.2
10	Lower respiratory infections	224	[202, 240]		-63.8

Rank	Agder	YLL rate	[95% UI]		Change (%)
1	Ischemic heart disease	1 067	[948, 1 200]		-78.1
2	Lung cancer	**693**	**[610, 786]**	↑	-42.5
3	Self-harm	597	[519, 674]		-46.4
4	COPD	**526**	**[454, 603]**	↑	4.3
5	Stroke	437	[387, 493]		-69.8
6	Prostate cancer	411	[328, 554]		-26.0
7	Colon and rectum cancer	394	[345, 447]		-25.3
8	Drug use disorders	270	[230, 337]		97.7
9	Road injuries	270	[234, 327]		-71.9
10	Lower respiratory infections	232	[204, 261]		-63.1

Rank	Innlandet	YLL rate	[95% UI]		Change (%)
1	Ischemic heart disease	1 307	[1 175, 1 450]		-74.2
2	Self-harm	**730**	**[648, 817]**	↑	-42.5
3	Lung cancer	512	[452, 575]		-27.9
4	Stroke	505	[448, 566]		-65.2
5	COPD	445	[382, 513]		32.7
6	Prostate cancer	440	[333, 544]		-22.9
7	Colon and rectum cancer	382	[335, 435]		-19.1
8	Road injuries	**270**	**[241, 305]**	↑	-70.1
9	Lower respiratory infections	247	[218, 276]		-64.5
10	Drug use disorders	241	[204, 300]		119.7

Rank	Møre og Romsdal	YLL rate	[95% UI]		Change (%)
1	Ischemic heart disease	1 079	[927, 1 242]		-74.7
2	Self-harm	543	[468, 628]		-36.7
3	Lung cancer	514	[433, 610]		-28.7
4	Colon and rectum cancer	392	[331, 460]		-18.9
5	Stroke	386	[329, 451]		-66.2
6	Prostate cancer	379	[299, 540]		-24.1
7	COPD	336	[280, 401]		30.7
8	Road injuries	**281**	**[240, 326]**	↑	-63.9
9	Neonatal disorders	220	[151, 280]		-66.2
10	Congenital birth defects	208	[162, 334]		-61.5

Rank	Nordland	YLL rate	[95% UI]		Change (%)
1	Ischemic heart disease	1 378	[1 192, 1 581]		-76.6
2	Self-harm	**748**	**[648, 863]**	↑	-31.6
3	Lung cancer	624	[532, 729]		-33.0
4	Stroke	470	[401, 545]		-64.4
5	Colon and rectum cancer	438	[372, 512]		-14.7
6	Prostate cancer	417	[330, 578]		-21.9
7	COPD	396	[324, 473]		26.6
8	Road injuries	**286**	**[241, 338]**	↑	-62.8
9	Drug use disorders	255	[208, 325]		178.2
10	Lower respiratory infections	234	[197, 271]		-59.4

Rank	Oslo	YLL rate	[95% UI]		Change (%)
1	Ischemic heart disease	1 031	[871, 1 220]		-80.4
2	Self-harm	525	[440, 628]		-58.2
3	Lung cancer	500	[411, 598]		-55.4
4	Drug use disorders	425	[341, 566]		-13.7
5	Prostate cancer	402	[315, 561]		-28.4
6	Stroke	395	[326, 471]		-70.5
7	Colon and rectum cancer	389	[320, 470]		-40.4
8	COPD	382	[306, 462]		-16.8
9	Lower respiratory infections	239	[199, 283]		-67.9
10	Dementia	218	[52, 579]		-6.3

Rank	Rogaland	YLL rate	[95% UI]		Change (%)
1	Ischemic heart disease	1 101	[962, 1 250]		-74.6
2	Lung cancer	664	[574, 767]		-24.0
3	Self-harm	562	[489, 638]		-41.4
4	Colon and rectum cancer	465	[404, 533]		-15.2
5	COPD	436	[362, 516]		30.1
6	Prostate cancer	428	[333, 577]		-21.7
7	Stroke	367	[316, 421]		-64.8
8	Drug use disorders	293	[243, 364]		91.1
9	Lower respiratory infections	231	[198, 264]		-61.0
10	Road injuries	206	[180, 237]		-68.2

Rank	Troms og Finnmark	YLL rate	[95% UI]		Change (%)
1	Ischemic heart disease	**1 398**	**[1 249, 1 558]**	↑	-77.4
2	Lung cancer	670	[593, 750]		-38.7
3	Self-harm	629	[555, 713]		-43.6
4	Stroke	475	[419, 537]		-66.7
5	COPD	433	[366, 500]		13.7
6	Colon and rectum cancer	384	[337, 433]		-19.9
7	Prostate cancer	369	[301, 530]		-19.4
8	Neonatal disorders	**353**	**[271, 449]**	↑	-73.3
9	Road injuries	**297**	**[264, 335]**	↑	-71.7
10	Congenital birth defects	284	[216, 407]		-67.3

Rank	Trøndelag	YLL rate	[95% UI]		Change (%)
1	Ischemic heart disease	1 288	[1 141, 1 468]		-75.3
2	Self-harm	618	[543, 703]		-46.6
3	Lung cancer	585	[507, 671]		-27.5
4	Stroke	462	[404, 534]		-65.7
5	Colon and rectum cancer	429	[368, 500]		-22.9
6	Prostate cancer	428	[346, 591]		-21.3
7	COPD	379	[321, 449]		15.3
8	Drug use disorders	248	[203, 310]		125.1
9	Road injuries	236	[201, 274]		-66.2
10	Lower respiratory infections	228	[194, 263]		-59.8

Rank	Vestfold og Telemark	YLL rate	[95% UI]		Change (%)
1	Ischemic heart disease	1 175	[1 052, 1 311]		-75.6
2	Self-harm	700	[621, 785]		-42.0
3	Lung cancer	647	[571, 727]		-32.1
4	COPD	498	[433, 571]		27.9
5	Stroke	439	[388, 493]		-67.2
6	Colon and rectum cancer	437	[384, 492]		-19.1
7	Prostate cancer	428	[337, 555]		-17.9
8	Drug use disorders	318	[271, 386]		106.4
9	Lower respiratory infections	232	[205, 259]		-61.9
10	Road injuries	220	[195, 245]		-68.9

Rank	Vestland	YLL rate	[95% UI]		Change (%)
1	Ischemic heart disease	1 179	[1 043, 1 334]		-74.3
2	Lung cancer	594	[518, 680]		-26.6
3	Self-harm	544	[474, 617]		-41.3
4	Colon and rectum cancer	469	[410, 537]		-18.3
5	Prostate cancer	418	[331, 574]		-18.7
6	Stroke	396	[345, 452]		-64.8
7	COPD	394	[332, 457]		15.9
8	Drug use disorders	347	[290, 436]		116.5
9	Lower respiratory infections	216	[187, 246]		-63.2
10	Road injuries	211	[185, 241]		-66.7

Rank	Viken	YLL rate	[95% UI]		Change (%)
1	Ischemic heart disease	1 036	[944, 1 138]		-77.9
2	Self-harm	539	[481, 598]		-49.9
3	Lung cancer	497	[447, 552]		-43.8
4	Stroke	392	[352, 432]		-70.8
5	Prostate cancer	383	[307, 509]		-27.1
6	Colon and rectum cancer	383	[341, 425]		-27.7
7	COPD	364	[319, 408]		5.9
8	Drug use disorders	297	[257, 370]		63.1
9	Road injuries	218	[195, 241]		-71.6
10	Lower respiratory infections	206	[181, 228]		-64.8

Communicable, maternal, neonatal, and nutritional diseases
Non-communicable diseases
Injuries

Figure S7. Age-standardised YLL rates per 100 000 male inhabitants in each county for the leading 10 level-3 causes in Norway and each Norwegian county, 2019. Bold rates indicate that the uncertainty interval of the county estimate does not overlap the uncertainty interval of the national estimate (upward arrow = county rate is above the national rate, downward arrow = county rate is below the national rate). Lung cancer = Tracheal, bronchus, and lung cancer, Dementia = Alzheimer's disease and other dementias, COPD = Chronic obstructive pulmonary disease, MSK = Musculoskeletal, Endocrine disorders = Endocrine, metabolic, blood, and immune disorders, YLL = Years of Lost Life.

Rank	Norway	YLL rate	[95% UI]	Change (%)
1	Ischemic heart disease	488	[423, 543]	-72.1
2	Lung cancer	460	[429, 489]	44.2
3	Breast cancer	371	[344, 400]	-43.1
4	Stroke	336	[296, 377]	-65.4
5	Colon and rectum cancer	326	[298, 354]	-17.4
6	COPD	306	[142, 349]	107.1
7	Self-harm	236	[221, 255]	-37.2
8	Dementia	224	[57, 570]	-8.5
9	Congenital birth defects	189	[158, 276]	-62.2
10	Ovarian cancer	187	[170, 208]	-25.4

Agder	YLL rate	[95% UI]		Change (%)
Lung cancer	**585**	**[503, 674]**	↑	53.0
Ischemic heart disease	481	[407, 564]		-71.6
Breast cancer	402	[345, 464]		-38.1
COPD	386	[161, 483]		99.9
Stroke	365	[309, 429]		-64.5
Colon and rectum cancer	341	[294, 398]		-9.6
Self-harm	253	[221, 293]		-30.5
Dementia	222	[55, 581]		-8.1
Ovarian cancer	205	[175, 241]		-19.1
Congenital birth defects	181	[148, 272]		-65.9

Rank	Innlandet	YLL rate	[95% UI]	Change (%)
1	Ischemic heart disease	571	[477, 657]	-69.7
2	Lung cancer	439	[380, 507]	64.4
3	Stroke	407	[344, 472]	-62.9
4	Breast cancer	355	[310, 406]	-38.1
5	COPD	331	[153, 407]	145.6
6	Colon and rectum cancer	296	[256, 336]	-10.6
7	Self-harm	253	[223, 284]	-30.8
8	Dementia	221	[56, 579]	-8.0
9	Ovarian cancer	217	[186, 251]	-23.2
10	Congenital birth defects	216	[176, 300]	-61.8

Møre og Romsdal	YLL rate	[95% UI]	Change (%)
Ischemic heart disease	499	[423, 585]	-68.6
Lung cancer	376	[318, 444]	62.2
Breast cancer	358	[302, 426]	-41.1
Colon and rectum cancer	338	[287, 395]	-13.7
Stroke	333	[280, 393]	-64.0
COPD	239	[125, 303]	130.9
Dementia	224	[55, 591]	-8.6
Congenital birth defects	211	[172, 310]	-58.6
Self-harm	**179**	**[149, 210]**	-39.0
Neonatal disorders	173	[123, 213]	-66.0

Rank	Nordland	YLL rate	[95% UI]	Change (%)
1	Ischemic heart disease	533	[449, 617]	-73.8
2	Lung cancer	493	[419, 581]	52.9
3	Breast cancer	339	[284, 399]	-40.4
4	Stroke	335	[281, 392]	-66.1
5	Colon and rectum cancer	319	[268, 376]	-11.5
6	COPD	308	[140, 391]	135.0
7	Dementia	223	[56, 597]	-7.9
8	Self-harm	201	[167, 236]	-33.8
9	Pancreatic cancer	177	[148, 210]	4.0
10	Ovarian cancer	175	[142, 225]	-25.6

Oslo	YLL rate	[95% UI]	Change (%)
Lung cancer	488	[415, 570]	4.5
Ischemic heart disease	417	[342, 487]	-76.5
Breast cancer	404	[344, 468]	-51.1
COPD	319	[142, 407]	52.9
Colon and rectum cancer	307	[262, 360]	-33.9
Stroke	304	[251, 356]	-68.7
Self-harm	283	[242, 331]	-51.1
Dementia	252	[63, 654]	-6.5
Congenital birth defects	183	[142, 285]	-66.6
Ovarian cancer	174	[143, 215]	-32.8

Rank	Rogaland	YLL rate	[95% UI]	Change (%)
1	Ischemic heart disease	445	[368, 519]	-72.7
2	Lung cancer	434	[370, 503]	45.8
3	Breast cancer	376	[320, 439]	-45.2
4	Colon and rectum cancer	342	[291, 399]	-21.1
5	COPD	280	[133, 355]	112.1
6	Stroke	278	[232, 326]	-66.2
7	Dementia	222	[56, 592]	-10.5
8	Ovarian cancer	208	[174, 246]	-26.1
9	Self-harm	201	[169, 235]	-45.3
10	Congenital birth defects	175	[139, 269]	-61.2

Troms og Finnmark	YLL rate	[95% UI]		Change (%)
Ischemic heart disease	531	[450, 616]		-72.4
Lung cancer	452	[393, 524]		53.4
Stroke	368	[311, 431]		-64.9
Breast cancer	336	[286, 389]		-40.2
Congenital birth defects	309	[254, 385]		-64.1
Colon and rectum cancer	304	[260, 352]		-10.6
COPD	304	[138, 376]		141.7
Neonatal disorders	**290**	**[233, 357]**	↑	-72.0
Dementia	220	[54, 578]		-6.3
Self-harm	217	[189, 245]		-32.2

Rank	Trøndelag	YLL rate	[95% UI]	Change (%)
1	Ischemic heart disease	548	[460, 636]	-70.7
2	Lung cancer	466	[398, 543]	66.7
3	Stroke	389	[326, 451]	-62.6
4	Breast cancer	386	[330, 450]	-40.6
5	Colon and rectum cancer	353	[303, 411]	-9.5
6	COPD	326	[156, 415]	116.2
7	Self-harm	223	[184, 262]	-34.4
8	Dementia	221	[53, 579]	-7.9
9	Pancreatic cancer	188	[158, 220]	14.8
10	Ovarian cancer	174	[145, 227]	-26.9

Vestfold og Telemark	YLL rate	[95% UI]	Change (%)
Ischemic heart disease	509	[435, 588]	-70.8
Lung cancer	504	[439, 575]	48.3
Breast cancer	378	[331, 435]	-40.4
COPD	353	[148, 438]	123.5
Stroke	351	[297, 409]	-64.8
Colon and rectum cancer	316	[273, 360]	-15.5
Self-harm	284	[250, 323]	-31.6
Dementia	221	[55, 569]	-8.8
Congenital birth defects	196	[159, 290]	-57.2
Ovarian cancer	192	[167, 224]	-22.7

Rank	Vestland	YLL rate	[95% UI]	Change (%)
1	Ischemic heart disease	468	[391, 543]	-70.3
2	Lung cancer	397	[344, 457]	68.3
3	Breast cancer	362	[317, 419]	-41.7
4	Colon and rectum cancer	355	[305, 409]	-17.5
5	Stroke	293	[243, 339]	-63.4
6	COPD	239	[130, 300]	99.0
7	Self-harm	227	[197, 261]	-27.5
8	Dementia	222	[56, 580]	-7.9
9	Congenital birth defects	203	[168, 288]	-58.1
10	Ovarian cancer	181	[153, 212]	-19.8

Viken	YLL rate	[95% UI]	Change (%)
Ischemic heart disease	462	[400, 525]	-73.2
Lung cancer	460	[415, 508]	41.2
Breast cancer	367	[330, 411]	-43.9
Stroke	328	[286, 373]	-67.8
Colon and rectum cancer	322	[289, 359]	-16.7
COPD	305	[138, 362]	117.6
Self-harm	229	[207, 255]	-36.8
Dementia	220	[56, 559]	-7.3
Ovarian cancer	187	[165, 215]	-27.6
Congenital birth defects	175	[142, 271]	-62.3

Communicable, maternal, neonatal, and nutritional diseases
Non-communicable diseases
Injuries

Figure S8. Age-standardised YLL rates per 100 000 female inhabitants in each county for the leading 10 level-3 causes in Norway and each Norwegian county, 2019. Bold rates indicate that the uncertainty interval of the county estimate does not overlap the uncertainty interval of the national estimate (upward arrow = county rate is above the national rate, downward arrow = county rate is below the national rate). Lung cancer = Tracheal, bronchus, and lung cancer, Dementia = Alzheimer's disease and other dementias, COPD = Chronic obstructive pulmonary disease, MSK = Musculoskeletal, Endocrine disorders = Endocrine, metabolic, blood, and immune disorders, YLL = Years of Lost Life.

Rank	Norway	YLD rate	[95% UI]	Change (%)
1	Low back pain	953	[663, 1 286]	-5.0
2	Headache disorders	777	[128, 1 713]	12.5
3	Anxiety disorders	644	[450, 884]	-5.9
4	Depressive disorders	552	[378, 757]	12.0
5	Falls	513	[365, 708]	8.9
6	Gynecological diseases	470	[319, 660]	-2.4
7	Endocrine disorders	411	[274, 572]	5.5
8	Diabetes mellitus	400	[275, 550]	36.0
9	Oral disorders	323	[188, 518]	-1.8
10	Age-related hearing loss	298	[198, 434]	-5.1

Rank	Agder	YLD rate	[95% UI]	Change (%)
1	Low back pain	953	[668, 1 290]	-4.9
2	Headache disorders	777	[129, 1 725]	12.9
3	Anxiety disorders	643	[449, 876]	-6.1
4	Depressive disorders	511	[353, 704]	11.8
5	Falls	509	[363, 699]	15.0
6	Gynecological diseases	475	[320, 660]	-2.3
7	Diabetes mellitus	442	[304, 608]	38.8
8	Endocrine disorders	410	[275, 572]	5.9
9	Oral disorders	323	[189, 518]	-1.7
10	Age-related hearing loss	298	[198, 436]	-5.4

Rank	Innlandet	YLD rate	[95% UI]	Change (%)
1	Low back pain	954	[663, 1 295]	-4.9
2	Headache disorders	773	[128, 1 709]	11.5
3	Anxiety disorders	643	[446, 885]	-6.2
4	Falls	519	[371, 715]	5.7
5	Depressive disorders	511	[352, 704]	12.1
6	Gynecological diseases	473	[320, 659]	-2.3
7	Diabetes mellitus	450	[307, 618]	37.7
8	Endocrine disorders	406	[271, 566]	5.7
9	Oral disorders	324	[189, 524]	-1.5
10	Age-related hearing loss	299	[200, 432]	-5.6

Rank	Møre og Romsdal	YLD rate	[95% UI]	Change (%)
1	Low back pain	953	[664, 1 291]	-5.1
2	Headache disorders	772	[127, 1 710]	12.9
3	Anxiety disorders	643	[454, 887]	-5.9
4	Falls	515	[370, 712]	3.6
5	Depressive disorders	510	[349, 702]	11.6
6	Gynecological diseases	462	[314, 642]	-2.9
7	Diabetes mellitus	421	[290, 576]	38.9
8	Endocrine disorders	413	[275, 582]	6.9
9	Oral disorders	324	[189, 518]	-2.2
10	Age-related hearing loss	300	[200, 438]	-5.5

Rank	Nordland	YLD rate	[95% UI]	Change (%)
1	Low back pain	953	[670, 1 289]	-5.1
2	Headache disorders	774	[128, 1 725]	13.2
3	Anxiety disorders	643	[455, 889]	-5.5
4	Depressive disorders	510	[350, 702]	12.0
5	Falls	501	[358, 691]	15.1
6	Gynecological diseases	469	[319, 655]	-1.4
7	Diabetes mellitus	442	[302, 606]	39.6
8	Endocrine disorders	411	[272, 570]	6.4
9	Oral disorders	323	[188, 519]	-2.1
10	Age-related hearing loss	299	[197, 436]	-4.8

Rank	Oslo	YLD rate	[95% UI]	Change (%)
1	Low back pain	950	[663, 1 282]	-5.2
2	Depressive disorders	808	[553, 1 109]	3.0
3	Headache disorders	778	[130, 1 732]	12.5
4	Anxiety disorders	645	[451, 887]	-5.9
5	Falls	523	[373, 718]	9.7
6	Gynecological diseases	470	[320, 663]	-3.7
7	Asthma	436	[273, 661]	-35.5
8	Endocrine disorders	410	[276, 574]	3.9
9	Diabetes mellitus	333	[228, 461]	31.9
10	Oral disorders	321	[186, 514]	-2.0

Rank	Rogaland	YLD rate	[95% UI]	Change (%)
1	Low back pain	951	[664, 1 284]	-5.2
2	Headache disorders	775	[127, 1 727]	12.9
3	Anxiety disorders	645	[451, 883]	-5.7
4	Falls	515	[366, 708]	4.8
5	Depressive disorders	511	[350, 700]	11.8
6	Gynecological diseases	465	[315, 649]	-2.6
7	Endocrine disorders	412	[275, 573]	5.2
8	Diabetes mellitus	389	[268, 536]	39.5
9	Oral disorders	322	[188, 515]	-1.9
10	Age-related hearing loss	298	[200, 434]	-5.5

Rank	Troms og Finnmark	YLD rate	[95% UI]	Change (%)
1	Low back pain	951	[668, 1 291]	-5.2
2	Headache disorders	772	[128, 1 723]	13.2
3	Anxiety disorders	642	[453, 877]	-5.8
4	Depressive disorders	510	[350, 702]	12.0
5	Falls	495	[353, 680]	7.2
6	Gynecological diseases	454	[307, 634]	-2.2
7	Diabetes mellitus	430	[293, 586]	36.9
8	Endocrine disorders	394	[263, 550]	4.8
9	Oral disorders	328	[190, 541]	-1.7
10	Age-related hearing loss	304	[204, 442]	-6.8

Rank	Trøndelag	YLD rate	[95% UI]	Change (%)
1	Low back pain	951	[663, 1 286]	-4.8
2	Headache disorders	791	[133, 1 734]	12.8
3	Anxiety disorders	642	[453, 883]	-6.5
4	Depressive disorders	511	[353, 703]	11.9
5	Falls	480	[332, 675]	15.2
6	Gynecological diseases	470	[316, 657]	-2.3
7	Endocrine disorders	416	[275, 579]	6.4
8	Diabetes mellitus	356	[244, 489]	25.5
9	Oral disorders	324	[190, 519]	-1.9
10	Age-related hearing loss	297	[198, 436]	-4.9

Rank	Vestfold og Telemark	YLD rate	[95% UI]	Change (%)
1	Low back pain	955	[664, 1 291]	-4.9
2	Headache disorders	777	[129, 1 716]	12.9
3	Anxiety disorders	645	[451, 886]	-6.2
4	Falls	533	[380, 734]	3.9
5	Depressive disorders	511	[351, 699]	12.0
6	Gynecological diseases	476	[321, 666]	-2.1
7	Diabetes mellitus	416	[284, 570]	34.7
8	Endocrine disorders	409	[274, 571]	5.6
9	Oral disorders	324	[189, 516]	-1.6
10	Age-related hearing loss	298	[199, 434]	-5.1

Rank	Vestland	YLD rate	[95% UI]	Change (%)
1	Low back pain	953	[671, 1 293]	-5.1
2	Headache disorders	774	[128, 1 721]	12.9
3	Anxiety disorders	645	[455, 877]	-5.6
4	Depressive disorders	514	[355, 702]	11.8
5	Falls	496	[353, 682]	15.9
6	Gynecological diseases	468	[317, 660]	-2.1
7	Endocrine disorders	418	[279, 584]	6.2
8	Diabetes mellitus	397	[272, 548]	41.4
9	Oral disorders	324	[189, 513]	-1.8
10	Age-related hearing loss	299	[199, 438]	-5.5

Rank	Viken	YLD rate	[95% UI]	Change (%)
1	Low back pain	954	[661, 1 291]	-4.6
2	Headache disorders	774	[129, 1 711]	11.5
3	Anxiety disorders	645	[452, 882]	-5.9
4	Falls	526	[376, 725]	6.5
5	Depressive disorders	512	[350, 702]	12.0
6	Gynecological diseases	475	[322, 660]	-2.3
7	Endocrine disorders	413	[277, 575]	4.8
8	Diabetes mellitus	399	[275, 552]	35.7
9	Oral disorders	323	[188, 517]	-1.4
10	Age-related hearing loss	297	[198, 433]	-4.8

Non-communicable diseases
Injuries

Figure S9. Age-standardised YLD rates per 100 000 inhabitants in each county for the leading 10 level-3 causes in Norway and each Norwegian county, both sexes combined, 2019. Bold rates indicate that the uncertainty interval of the county estimate does not overlap the uncertainty interval of the national estimate (upward arrow = county rate is above the national rate, downward arrow = county rate is below the national rate). Lung cancer = Tracheal, bronchus, and lung cancer, Dementia = Alzheimer's disease and other dementias, COPD = Chronic obstructive pulmonary disease, MSK = Musculoskeletal, Endocrine disorders = Endocrine, metabolic, blood, and immune disorders, YLD = Years Lived with Disease.

Rank	Norway	YLD rate	[95% UI]	Change (%)
1	Low back pain	841	[583, 1 141]	-5.2
2	Headache disorders	523	[88, 1 164]	12.9
3	Falls	507	[360, 703]	0.7
4	Anxiety disorders	496	[351, 688]	-0.9
5	Depressive disorders	439	[300, 599]	9.7
6	Diabetes mellitus	428	[292, 586]	32.0
7	Age-related hearing loss	337	[223, 498]	-5.3
8	Endocrine disorders	333	[222, 464]	9.0
9	Oral disorders	295	[167, 486]	-0.2
10	Drug use disorders	278	[193, 369]	35.3

Agder	YLD rate	[95% UI]	Change (%)
Low back pain	840	[587, 1 143]	-5.2
Headache disorders	524	[86, 1 172]	13.1
Falls	501	[354, 698]	5.5
Anxiety disorders	496	[346, 678]	-0.8
Diabetes mellitus	475	[323, 649]	33.5
Depressive disorders	421	[289, 576]	10.1
Age-related hearing loss	337	[222, 501]	-5.5
Endocrine disorders	331	[221, 461]	9.6
Oral disorders	294	[168, 485]	0.0
Drug use disorders	248	[172, 334]	28.5

Rank	Innlandet	YLD rate	[95% UI]	Change (%)
1	Low back pain	842	[582, 1 141]	-5.3
2	Falls	513	[363, 713]	-2.5
3	Headache disorders	508	[87, 1 127]	13.4
4	Anxiety disorders	495	[344, 690]	-1.3
5	Diabetes mellitus	479	[326, 650]	36.8
6	Depressive disorders	421	[287, 577]	10.1
7	Age-related hearing loss	338	[222, 496]	-5.5
8	Endocrine disorders	329	[219, 458]	9.1
9	Oral disorders	295	[169, 489]	-0.2
10	Drug use disorders	253	[174, 338]	41.2

Møre og Romsdal	YLD rate	[95% UI]	Change (%)
Low back pain	843	[579, 1 146]	-5.2
Headache disorders	524	[87, 1 167]	13.2
Falls	523	[372, 724]	-4.3
Anxiety disorders	497	[347, 687]	-0.9
Diabetes mellitus	453	[309, 618]	35.5
Depressive disorders	421	[288, 582]	10.2
Age-related hearing loss	338	[221, 494]	-5.9
Endocrine disorders	336	[225, 472]	10.4
Oral disorders	295	[168, 485]	-0.5
Alcohol use disorders	235	[157, 331]	-40.1

Rank	Nordland	YLD rate	[95% UI]	Change (%)
1	Low back pain	842	[587, 1 154]	-5.4
2	Headache disorders	524	[85, 1 154]	13.0
3	Falls	498	[356, 690]	9.2
4	Anxiety disorders	497	[352, 690]	-0.9
5	Diabetes mellitus	476	[322, 654]	37.6
6	Depressive disorders	421	[288, 579]	9.9
7	Age-related hearing loss	337	[220, 494]	-5.1
8	Endocrine disorders	334	[223, 465]	9.5
9	Oral disorders	294	[168, 488]	-0.4
10	Asthma	272	[172, 411]	-48.5

Oslo	YLD rate	[95% UI]	Change (%)
Low back pain	837	[587, 1 126]	-5.0
Depressive disorders	554	[382, 768]	1.6
Headache disorders	522	[87, 1 164]	13.3
Falls	512	[364, 706]	-0.3
Anxiety disorders	496	[342, 694]	-0.5
Asthma	423	[262, 637]	-40.0
Diabetes mellitus	362	[246, 502]	23.8
Endocrine disorders	332	[220, 468]	8.0
Age-related hearing loss	331	[217, 492]	-4.6
Drug use disorders	324	[224, 437]	2.3

Rank	Rogaland	YLD rate	[95% UI]	Change (%)
1	Low back pain	840	[584, 1 140]	-5.4
2	Headache disorders	524	[87, 1 156]	13.2
3	Falls	510	[362, 705]	-3.0
4	Anxiety disorders	497	[346, 687]	-0.8
5	Depressive disorders	421	[289, 580]	10.0
6	Diabetes mellitus	416	[287, 575]	36.6
7	Age-related hearing loss	337	[226, 494]	-5.9
8	Endocrine disorders	334	[222, 468]	9.0
9	Oral disorders	294	[167, 485]	-0.1
10	Drug use disorders	277	[189, 374]	40.9

Troms og Finnmark	YLD rate	[95% UI]	Change (%)
Low back pain	841	[587, 1 138]	-5.6
Headache disorders	524	[87, 1 156]	13.2
Anxiety disorders	497	[351, 680]	-1.0
Falls	493	[350, 685]	-0.4
Diabetes mellitus	457	[310, 624]	34.4
Depressive disorders	421	[288, 575]	10.1
Age-related hearing loss	342	[228, 501]	-6.8
Endocrine disorders	319	[212, 442]	8.2
Oral disorders	304	[170, 515]	-0.3
Alcohol use disorders	246	[166, 347]	-40.0

Rank	Trøndelag	YLD rate	[95% UI]	Change (%)
1	Low back pain	840	[590, 1 137]	-5.1
2	Headache disorders	542	[94, 1 197]	13.4
3	Anxiety disorders	496	[349, 684]	-1.1
4	Falls	475	[328, 675]	7.7
5	Depressive disorders	421	[290, 582]	10.1
6	Diabetes mellitus	386	[261, 537]	24.0
7	Age-related hearing loss	336	[221, 499]	-4.9
8	Endocrine disorders	335	[223, 468]	9.8
9	Oral disorders	295	[168, 487]	-0.2
10	Drug use disorders	243	[167, 324]	38.2

Vestfold og Telemark	YLD rate	[95% UI]	Change (%)
Low back pain	841	[583, 1 145]	-5.3
Falls	534	[381, 741]	-3.0
Headache disorders	523	[88, 1 157]	13.1
Anxiety disorders	497	[347, 693]	-1.1
Diabetes mellitus	449	[307, 611]	32.0
Depressive disorders	420	[288, 576]	9.9
Age-related hearing loss	337	[222, 498]	-5.1
Endocrine disorders	330	[222, 458]	8.7
Oral disorders	294	[167, 482]	-0.2
Drug use disorders	285	[196, 388]	47.0

Rank	Vestland	YLD rate	[95% UI]	Change (%)
1	Low back pain	841	[588, 1 148]	-5.3
2	Headache disorders	524	[88, 1 153]	13.0
3	Anxiety disorders	497	[349, 681]	-0.8
4	Falls	488	[347, 679]	8.4
5	Depressive disorders	423	[288, 584]	9.7
6	Diabetes mellitus	421	[284, 582]	37.2
7	Endocrine disorders	339	[225, 474]	9.5
8	Age-related hearing loss	337	[223, 500]	-5.7
9	Drug use disorders	297	[206, 401]	50.9
10	Oral disorders	295	[169, 485]	-0.2

Viken	YLD rate	[95% UI]	Change (%)
Low back pain	841	[584, 1 141]	-4.8
Headache disorders	520	[88, 1 135]	11.8
Falls	517	[366, 717]	-2.5
Anxiety disorders	496	[349, 685]	-0.9
Diabetes mellitus	426	[292, 579]	31.4
Depressive disorders	421	[287, 577]	10.2
Age-related hearing loss	336	[223, 492]	-4.7
Endocrine disorders	334	[222, 468]	8.2
Drug use disorders	299	[208, 405]	38.5
Oral disorders	294	[168, 483]	0.0

Non-communicable diseases
Injuries

Figure S10. Age-standardised YLD rates per 100 000 male inhabitants in each county for the leading 10 level-3 causes in Norway and each Norwegian county, 2019. Bold rates indicate that the uncertainty interval of the county estimate does not overlap the uncertainty interval of the national estimate (upward arrow = county rate is above the national rate, downward arrow = county rate is below the national rate). Lung cancer = Tracheal, bronchus, and lung cancer, Dementia = Alzheimer's disease and other dementias, COPD = Chronic obstructive pulmonary disease, MSK = Musculoskeletal, Endocrine disorders = Endocrine, metabolic, blood, and immune disorders, YLD = Years Lived with Disease.

Rank	Norway	YLD rate	[95% UI]	Change (%)
1	Low back pain	1 065	[744, 1 434]	-4.0
2	Headache disorders	1 043	[168, 2 343]	12.9
3	Gynecological diseases	964	[653, 1 351]	-1.2
4	Anxiety disorders	798	[559, 1 094]	-8.6
5	Depressive disorders	671	[459, 918]	14.3
6	Falls	507	[361, 694]	19.6
7	Endocrine disorders	493	[329, 688]	3.7
8	Other MSK disorders	391	[264, 548]	-9.7
9	Diabetes mellitus	371	[257, 511]	39.7
10	Osteoarthritis	364	[185, 734]	4.8

Rank	Agder	YLD rate	[95% UI]	Change (%)
1	Low back pain	1 067	[742, 1 440]	-3.9
2	Headache disorders	1 043	[172, 2 344]	13.4
3	Gynecological diseases	969	[652, 1 350]	-1.3
4	Anxiety disorders	796	[554, 1 090]	-9.0
5	Depressive disorders	606	[418, 838]	13.7
6	Falls	505	[360, 692]	26.9
7	Endocrine disorders	490	[327, 686]	4.0
8	Diabetes mellitus	408	[282, 570]	44.4
9	Other MSK disorders	388	[262, 550]	-9.5
10	Osteoarthritis	359	[182, 724]	4.7

Rank	Innlandet	YLD rate	[95% UI]	Change (%)
1	Low back pain	1 066	[744, 1 432]	-4.1
2	Headache disorders	1 051	[171, 2 380]	11.1
3	Gynecological diseases	966	[653, 1 347]	-1.3
4	Anxiety disorders	797	[552, 1 091]	-8.9
5	Depressive disorders	606	[416, 840]	13.9
6	Falls	513	[366, 701]	15.3
7	Endocrine disorders	484	[322, 674]	3.5
8	Diabetes mellitus	420	[293, 574]	38.6
9	Other MSK disorders	380	[258, 535]	-9.7
10	Osteoarthritis	359	[182, 725]	4.8

Rank	Møre og Romsdal	YLD rate	[95% UI]	Change (%)
1	Low back pain	1 067	[753, 1 445]	-4.2
2	Headache disorders	1 043	[168, 2 345]	13.4
3	Gynecological diseases	967	[657, 1 343]	-1.2
4	Anxiety disorders	800	[558, 1 097]	-8.5
5	Depressive disorders	606	[417, 831]	13.4
6	Falls	497	[358, 680]	14.6
7	Endocrine disorders	496	[327, 704]	5.1
8	Diabetes mellitus	389	[267, 540]	41.9
9	Other MSK disorders	389	[256, 547]	-9.6
10	Osteoarthritis	361	[183, 730]	5.1

Rank	Nordland	YLD rate	[95% UI]	Change (%)
1	Low back pain	1 067	[747, 1 440]	-4.2
2	Headache disorders	1 043	[169, 2 363]	13.5
3	Gynecological diseases	971	[661, 1 356]	-1.0
4	Anxiety disorders	799	[559, 1 103]	-8.2
5	Depressive disorders	607	[417, 841]	13.9
6	Falls	493	[352, 676]	23.1
7	Endocrine disorders	490	[325, 688]	4.3
8	Diabetes mellitus	407	[279, 560]	41.3
9	Other MSK disorders	390	[259, 558]	-10.1
10	Osteoarthritis	360	[183, 730]	4.8

Rank	Oslo	YLD rate	[95% UI]	Change (%)
1	Depressive disorders	1 062	[724, 1 462]	5.4
2	Low back pain	1 059	[741, 1 431]	-3.9
3	Headache disorders	1 037	[170, 2 344]	13.5
4	Gynecological diseases	951	[649, 1 344]	-1.2
5	Anxiety disorders	795	[554, 1 090]	-8.2
6	Falls	520	[372, 710]	23.0
7	Endocrine disorders	492	[330, 686]	3.0
8	Asthma	447	[278, 681]	-31.7
9	Other MSK disorders	399	[265, 565]	-8.7
10	Osteoarthritis	390	[197, 792]	5.0

Rank	Rogaland	YLD rate	[95% UI]	Change (%)
1	Low back pain	1 065	[744, 1 429]	-4.2
2	Headache disorders	1 043	[168, 2 345]	13.5
3	Gynecological diseases	959	[651, 1 341]	-1.3
4	Anxiety disorders	800	[557, 1 087]	-8.3
5	Depressive disorders	606	[413, 838]	13.7
6	Falls	508	[365, 696]	15.1
7	Endocrine disorders	494	[331, 698]	3.4
8	Other MSK disorders	391	[264, 558]	-9.7
9	Diabetes mellitus	362	[249, 502]	42.0
10	Osteoarthritis	361	[183, 728]	5.0

Rank	Troms og Finnmark	YLD rate	[95% UI]	Change (%)
1	Low back pain	1 067	[752, 1 448]	-4.3
2	Headache disorders	1 043	[170, 2 332]	13.5
3	Gynecological diseases	948	[642, 1 324]	-1.9
4	Anxiety disorders	799	[559, 1 096]	-8.6
5	Depressive disorders	607	[414, 840]	13.9
6	Falls	486	[346, 664]	17.6
7	Endocrine disorders	474	[317, 667]	2.4
8	Diabetes mellitus	402	[274, 554]	39.6
9	Other MSK disorders	381	[255, 527]	-6.8
10	Osteoarthritis	358	[182, 719]	5.6

Rank	Trøndelag	YLD rate	[95% UI]	Change (%)
1	Low back pain	1 065	[749, 1 441]	-3.8
2	Headache disorders	1 057	[176, 2 353]	13.3
3	Gynecological diseases	972	[654, 1 360]	-0.7
4	Anxiety disorders	797	[556, 1 093]	-9.3
5	Depressive disorders	607	[416, 835]	14.0
6	Endocrine disorders	499	[332, 699]	4.6
7	Falls	475	[327, 666]	24.8
8	Other MSK disorders	409	[277, 570]	-10.4
9	Osteoarthritis	362	[185, 735]	5.1
10	Oral disorders	352	[209, 550]	-2.3

Rank	Vestfold og Telemark	YLD rate	[95% UI]	Change (%)
1	Low back pain	1 067	[743, 1 439]	-4.0
2	Headache disorders	1 042	[168, 2 341]	13.4
3	Gynecological diseases	965	[651, 1 353]	-1.2
4	Anxiety disorders	798	[555, 1 100]	-8.8
5	Depressive disorders	606	[416, 833]	14.0
6	Falls	519	[373, 707]	13.3
7	Endocrine disorders	488	[327, 684]	3.7
8	Other MSK disorders	387	[257, 541]	-9.9
9	Diabetes mellitus	384	[262, 528]	37.3
10	Osteoarthritis	360	[183, 725]	4.9

Rank	Vestland	YLD rate	[95% UI]	Change (%)
1	Low back pain	1 067	[751, 1 447]	-4.0
2	Headache disorders	1 042	[171, 2 337]	13.3
3	Gynecological diseases	970	[659, 1 369]	-0.9
4	Anxiety disorders	800	[561, 1 084]	-8.3
5	Depressive disorders	611	[418, 838]	13.9
6	Endocrine disorders	502	[335, 705]	4.6
7	Falls	492	[349, 674]	25.1
8	Other MSK disorders	394	[266, 555]	-9.7
9	Diabetes mellitus	371	[253, 514]	45.2
10	Osteoarthritis	361	[183, 728]	4.9

Rank	Viken	YLD rate	[95% UI]	Change (%)
1	Low back pain	1 066	[747, 1 437]	-3.8
2	Headache disorders	1 039	[168, 2 350]	12.0
3	Gynecological diseases	965	[654, 1 343]	-1.1
4	Anxiety disorders	798	[556, 1 092]	-8.4
5	Depressive disorders	607	[417, 833]	13.9
6	Falls	522	[375, 715]	17.9
7	Endocrine disorders	494	[332, 691]	3.1
8	Other MSK disorders	388	[261, 543]	-10.8
9	Diabetes mellitus	373	[260, 516]	40.1
10	Osteoarthritis	361	[183, 726]	4.9

Non-communicable diseases
Injuries

Figure S11. Age-standardised YLD rates per 100 000 female inhabitants in each county for the leading 10 level-3 causes in Norway and each Norwegian county, 2019. Bold rates indicate that the uncertainty interval of the county estimate does not overlap the uncertainty interval of the national estimate (upward arrow = county rate is above the national rate, downward arrow = county rate is below the national rate). Lung cancer = Tracheal, bronchus, and lung cancer, Dementia = Alzheimer's disease and other dementias, COPD = Chronic obstructive pulmonary disease, MSK = Musculoskeletal, Endocrine disorders = Endocrine, metabolic, blood, and immune disorders, YLD = Years Lived with Disease.

Rank	Norway	PAF	[95% UI]	Change (%)
1	Tobacco	9.9	[8.9, 11.0]	-26.4
2	High blood pressure	7.5	[6.2, 8.7]	-15.8
3	Dietary risks	6.8	[5.8, 8.1]	-4.3
4	High fasting plasma glucose	6.8	[5.5, 8.7]	36.7
5	Alcohol use	5.5	[4.7, 6.5]	30.1
6	High body-mass index	5.3	[3.1, 7.8]	33.8
7	High LDL	3.9	[3.1, 4.9]	-2.9
8	Occupational risks	3.8	[3.4, 4.2]	-11.7
9	Drug use	3.4	[2.9, 3.9]	37.6
10	Malnutrition	1.9	[1.6, 2.2]	-4.3

Rank	Agder	PAF	[95% UI]	Change (%)
1	Tobacco	10.4	[9.2, 11.6]	-25.8
2	High blood pressure	7.3	[6.1, 8.6]	-15.2
3	High fasting plasma glucose	7.3	[5.9, 9.2]	38.1
4	Dietary risks	6.6	[5.7, 7.9]	-3.9
5	Alcohol use	5.6	[4.4, 6.8]	35.6
6	High body-mass index	5.3	[3.1, 7.7]	36.0
7	Occupational risks	4.1	[3.6, 4.7]	-10.9
8	High LDL	3.7	[2.9, 4.6]	-3.6
9	Drug use	3.0	[2.6, 3.5]	29.7
10	Malnutrition	1.8	[1.5, 2.2]	-4.0

Rank	Innlandet	PAF	[95% UI]	Change (%)
1	Tobacco	9.7	[8.6, 10.9]	-24.4
2	High blood pressure	8.0	[6.6, 9.5]	-10.6
3	High fasting plasma glucose	7.8	[6.3, 9.8]	42.3
4	Dietary risks	7.4	[6.3, 8.8]	-3.3
5	High body-mass index	5.6	[3.2, 8.2]	34.5
6	Alcohol use	5.4	[4.1, 6.9]	29.0
7	High LDL	4.3	[3.4, 5.3]	-0.8
8	Occupational risks	3.6	[3.2, 4.1]	-10.3
9	Drug use	2.9	[2.5, 3.3]	43.4
10	Malnutrition	1.8	[1.5, 2.2]	-4.6

Rank	Møre og Romsdal	PAF	[95% UI]	Change (%)
1	Tobacco	9.2	[8.1, 10.4]	-24.6
2	High blood pressure	7.2	[6.0, 8.6]	-14.7
3	High fasting plasma glucose	7.1	[5.6, 9.0]	40.7
4	Dietary risks	6.7	[5.7, 8.0]	-3.8
5	High body-mass index	5.4	[3.1, 7.9]	39.9
6	Alcohol use	5.2	[3.7, 6.7]	31.9
7	High LDL	3.8	[3.0, 4.8]	-3.2
8	Occupational risks	3.5	[3.0, 4.0]	-13.3
9	Drug use	2.6	[2.2, 3.0]	39.3
10	Malnutrition	2.1	[1.7, 2.6]	-4.6

Rank	Nordland	PAF	[95% UI]	Change (%)
1	Tobacco	10.0	[8.8, 11.2]	-26.2
2	High blood pressure	8.3	[6.8, 10.0]	-14.8
3	High fasting plasma glucose	7.7	[6.1, 9.8]	42.7
4	Dietary risks	7.6	[6.4, 9.0]	-3.7
5	High body-mass index	5.7	[3.3, 8.3]	33.8
6	Alcohol use	5.5	[3.9, 7.2]	31.1
7	High LDL	4.5	[3.5, 5.5]	-3.9
8	Occupational risks	3.5	[3.1, 4.0]	-12.2
9	Drug use	2.8	[2.4, 3.2]	40.7
10	Kidney dysfunction	2.0	[1.7, 2.4]	4.4

Rank	Oslo	PAF	[95% UI]	Change (%)
1	Tobacco	9.9	[8.7, 11.2]	-26.1
2	Alcohol use	6.5	[5.1, 7.9]	21.6
3	High blood pressure	6.2	[4.9, 7.5]	-13.3
4	Dietary risks	6.1	[5.0, 7.3]	-4.1
5	High fasting plasma glucose	5.5	[4.3, 7.0]	26.4
6	High body-mass index	5.2	[3.0, 7.6]	28.9
7	Drug use	4.4	[3.7, 5.2]	5.0
8	Occupational risks	3.7	[3.2, 4.2]	-13.6
9	High LDL	3.5	[2.7, 4.4]	-2.1
10	Malnutrition	1.9	[1.5, 2.3]	-4.2

Rank	Rogaland	PAF	[95% UI]	Change (%)
1	Tobacco	10.2	[9.0, 11.4]	-24.7
2	High blood pressure	7.2	[5.9, 8.6]	-14.6
3	Dietary risks	6.6	[5.5, 7.8]	-4.6
4	High fasting plasma glucose	6.5	[5.1, 8.5]	41.6
5	Alcohol use	5.4	[4.0, 6.8]	29.7
6	High body-mass index	5.0	[2.8, 7.3]	35.1
7	Occupational risks	4.0	[3.5, 4.5]	-13.2
8	High LDL	3.7	[2.9, 4.6]	-3.2
9	Drug use	3.3	[2.8, 3.9]	42.5
10	Malnutrition	2.0	[1.6, 2.4]	-3.9

Rank	Troms og Finnmark	PAF	[95% UI]	Change (%)
1	Tobacco	10.4	[9.3, 11.6]	-24.6
2	High blood pressure	8.1	[6.8, 9.6]	-18.5
3	Dietary risks	7.3	[6.2, 8.8]	-4.5
4	High fasting plasma glucose	7.3	[5.8, 9.2]	39.8
5	High body-mass index	5.3	[3.0, 7.8]	47.7
6	Alcohol use	5.1	[3.9, 6.4]	36.3
7	High LDL	4.5	[3.6, 5.5]	-3.0
8	Occupational risks	3.5	[3.1, 4.0]	-12.6
9	Malnutrition	2.5	[2.1, 3.0]	-5.6
10	Drug use	2.3	[2.0, 2.6]	34.5

Rank	Trøndelag	PAF	[95% UI]	Change (%)
1	Tobacco	10.6	[9.3, 11.9]	-26.2
2	High blood pressure	9.4	[7.9, 11.1]	-19.6
3	Dietary risks	7.3	[6.1, 8.9]	-5.8
4	High fasting plasma glucose	6.6	[5.2, 8.6]	27.5
5	Alcohol use	5.5	[4.0, 7.1]	36.5
6	High body-mass index	5.5	[3.2, 8.0]	35.5
7	High LDL	4.6	[3.6, 5.7]	-0.6
8	Occupational risks	3.5	[3.0, 4.0]	-12.8
9	Drug use	2.9	[2.5, 3.4]	41.3
10	Kidney dysfunction	1.9	[1.6, 2.2]	-2.0

Rank	Vestfold og Telemark	PAF	[95% UI]	Change (%)
1	Tobacco	10.2	[9.1, 11.4]	-25.4
2	High blood pressure	7.5	[6.3, 8.9]	-15.0
3	High fasting plasma glucose	7.0	[5.6, 8.8]	36.7
4	Dietary risks	6.8	[5.8, 8.2]	-4.0
5	Alcohol use	5.6	[4.3, 7.0]	27.9
6	High body-mass index	5.3	[3.1, 7.7]	32.5
7	Occupational risks	4.2	[3.7, 4.7]	-10.2
8	High LDL	3.9	[3.0, 4.8]	-4.0
9	Drug use	3.4	[3.0, 3.9]	49.8
10	Kidney dysfunction	1.8	[1.5, 2.1]	4.4

Rank	Vestland	PAF	[95% UI]	Change (%)
1	Tobacco	9.8	[8.7, 10.9]	-29.8
2	High blood pressure	7.4	[6.0, 8.8]	-12.2
3	Dietary risks	7.0	[5.9, 8.3]	-3.6
4	High fasting plasma glucose	6.7	[5.3, 8.6]	42.1
5	High body-mass index	5.2	[3.0, 7.6]	37.0
6	Alcohol use	5.1	[3.9, 6.4]	37.1
7	High LDL	4.1	[3.2, 5.1]	-0.9
8	Occupational risks	3.8	[3.4, 4.3]	-11.2
9	Drug use	3.8	[3.2, 4.4]	52.8
10	Malnutrition	1.9	[1.6, 2.3]	-5.0

Rank	Viken	PAF	[95% UI]	Change (%)
1	Tobacco	9.5	[8.4, 10.5]	-28.0
2	High blood pressure	6.9	[5.7, 8.2]	-16.4
3	High fasting plasma glucose	6.7	[5.4, 8.6]	36.1
4	Dietary risks	6.6	[5.5, 7.8]	-3.9
5	Alcohol use	5.5	[4.5, 6.6]	21.5
6	High body-mass index	5.3	[3.0, 7.7]	27.4
7	Occupational risks	3.9	[3.4, 4.3]	-11.0
8	High LDL	3.6	[2.8, 4.6]	-3.7
9	Drug use	3.6	[3.1, 4.2]	41.1
10	Malnutrition	1.9	[1.6, 2.3]	-4.1

Behavioural risks
Environmental & occupational risks
Metabolic risks

Figure S12. Leading 10 level-2 risk factors for Norway and each Norwegian county by population attributable fraction (PAF) for all-cause DALY rate per 100 000 male inhabitants. Age-standardised. Change = percentage change in summary exposure variable (SEV) between 1990 and 2019. DALY = Disability-Adjusted Life Year.

Rank	Norway	PAF	[95% UI]	Change (%)
1	Tobacco	6.6	[5.7, 7.6]	-28.0
2	High fasting plasma glucose	5.0	[3.9, 6.5]	39.6
3	High body-mass index	4.8	[3.2, 6.5]	38.4
4	High blood pressure	4.3	[3.4, 5.3]	-16.8
5	Dietary risks	4.0	[3.3, 4.7]	-7.1
6	Occupational risks	2.2	[1.9, 2.5]	-25.6
7	High LDL	1.9	[1.4, 2.5]	-2.8
8	Malnutrition	1.8	[1.5, 2.0]	-12.1
9	Alcohol use	1.7	[1.3, 2.2]	22.7
10	Drug use	1.5	[1.2, 1.8]	37.9

Rank	Agder	PAF	[95% UI]	Change (%)
1	Tobacco	7.0	[5.9, 8.2]	-27.4
2	High fasting plasma glucose	5.5	[4.2, 7.1]	45.7
3	High body-mass index	4.8	[3.2, 6.6]	40.6
4	High blood pressure	4.4	[3.3, 5.4]	-19.2
5	Dietary risks	4.1	[3.4, 4.8]	-7.3
6	Occupational risks	2.3	[1.9, 2.7]	-43.7
7	High LDL	1.9	[1.3, 2.5]	-3.2
8	Malnutrition	1.7	[1.4, 2.0]	-12.2
9	Alcohol use	1.6	[1.0, 2.4]	24.3
10	Drug use	1.4	[1.1, 1.6]	33.6

Rank	Innlandet	PAF	[95% UI]	Change (%)
1	Tobacco	6.5	[5.5, 7.6]	-25.7
2	High fasting plasma glucose	5.9	[4.6, 7.6]	40.2
3	High body-mass index	5.1	[3.3, 6.9]	39.0
4	High blood pressure	4.8	[3.7, 6.1]	-12.9
5	Dietary risks	4.3	[3.6, 5.2]	-7.0
6	Occupational risks	2.2	[1.9, 2.6]	-36.5
7	High LDL	2.1	[1.5, 2.9]	-2.4
8	Malnutrition	1.7	[1.4, 2.0]	-12.4
9	Alcohol use	1.5	[0.9, 2.2]	20.4
10	Drug use	1.4	[1.1, 1.6]	36.2

Rank	Møre og Romsdal	PAF	[95% UI]	Change (%)
1	Tobacco	6.0	[4.9, 7.1]	-26.4
2	High fasting plasma glucose	5.3	[4.1, 7.0]	42.1
3	High body-mass index	4.9	[3.2, 6.7]	44.8
4	High blood pressure	4.4	[3.3, 5.6]	-19.4
5	Dietary risks	4.1	[3.4, 5.0]	-7.5
6	Occupational risks	2.0	[1.7, 2.6]	-3.8
7	High LDL	2.0	[1.4, 2.7]	-3.2
8	Malnutrition	1.9	[1.5, 2.2]	-11.5
9	Alcohol use	1.4	[0.8, 2.3]	22.9
10	Low bone mineral density	1.3	[1.0, 1.5]	-14.0

Rank	Nordland	PAF	[95% UI]	Change (%)
1	Tobacco	6.7	[5.5, 8.0]	-27.7
2	High fasting plasma glucose	5.6	[4.3, 7.3]	42.6
3	High body-mass index	5.1	[3.3, 7.0]	38.0
4	High blood pressure	4.5	[3.5, 5.8]	-20.6
5	Dietary risks	4.2	[3.5, 5.0]	-7.0
6	Occupational risks	2.1	[1.7, 2.7]	-3.1
7	High LDL	2.0	[1.4, 2.7]	-3.8
8	Malnutrition	1.6	[1.3, 1.9]	-10.3
9	Alcohol use	1.5	[0.9, 2.4]	19.1
10	Low bone mineral density	1.3	[1.0, 1.5]	-13.9

Rank	Oslo	PAF	[95% UI]	Change (%)
1	Tobacco	6.6	[5.5, 7.7]	-28.4
2	High body-mass index	4.6	[3.0, 6.4]	33.4
3	High fasting plasma glucose	3.8	[2.9, 5.1]	34.0
4	High blood pressure	3.8	[2.9, 4.8]	-8.6
5	Dietary risks	3.3	[2.8, 4.1]	-6.5
6	Alcohol use	2.3	[1.5, 3.2]	26.8
7	Occupational risks	2.0	[1.7, 2.5]	-4.6
8	Drug use	2.0	[1.6, 2.3]	25.1
9	Malnutrition	1.7	[1.4, 2.1]	-12.6
10	High LDL	1.6	[1.1, 2.2]	-2.2

Rank	Rogaland	PAF	[95% UI]	Change (%)
1	Tobacco	6.4	[5.2, 7.6]	-26.0
2	High fasting plasma glucose	4.8	[3.7, 6.3]	41.3
3	High body-mass index	4.6	[2.9, 6.3]	41.0
4	High blood pressure	4.1	[3.1, 5.2]	-20.4
5	Dietary risks	3.8	[3.1, 4.6]	-7.3
6	Occupational risks	2.0	[1.7, 2.6]	-4.6
7	Malnutrition	1.8	[1.5, 2.2]	-11.8
8	High LDL	1.8	[1.3, 2.4]	-3.3
9	Alcohol use	1.6	[1.0, 2.4]	20.8
10	Drug use	1.5	[1.2, 1.8]	39.7

Rank	Troms og Finnmark	PAF	[95% UI]	Change (%)
1	Tobacco	6.6	[5.5, 7.6]	-25.8
2	High fasting plasma glucose	5.4	[4.2, 7.0]	41.3
3	High body-mass index	4.7	[3.1, 6.6]	50.6
4	High blood pressure	4.5	[3.5, 5.6]	-21.9
5	Dietary risks	4.1	[3.4, 4.9]	-7.3
6	Occupational risks	2.2	[1.9, 2.6]	-44.2
7	Malnutrition	2.2	[1.8, 2.6]	-17.6
8	High LDL	2.0	[1.5, 2.7]	-3.0
9	Alcohol use	1.4	[0.9, 2.0]	28.5
10	Drug use	1.3	[1.0, 1.5]	30.9

Rank	Trøndelag	PAF	[95% UI]	Change (%)
1	Tobacco	8.3	[7.0, 9.6]	-27.6
2	High body-mass index	4.9	[3.2, 6.7]	40.2
3	High fasting plasma glucose	4.7	[3.7, 6.2]	26.0
4	High blood pressure	4.5	[3.3, 5.8]	-22.9
5	Dietary risks	4.2	[3.5, 5.1]	-7.1
6	High LDL	2.1	[1.5, 2.8]	0.6
7	Occupational risks	2.0	[1.7, 2.6]	-3.1
8	Alcohol use	1.8	[1.1, 2.7]	22.8
9	Malnutrition	1.5	[1.3, 1.9]	-11.1
10	Kidney dysfunction	1.2	[1.0, 1.5]	2.7

Rank	Vestfold og Telemark	PAF	[95% UI]	Change (%)
1	Tobacco	6.7	[5.6, 7.8]	-26.9
2	High fasting plasma glucose	5.2	[4.1, 6.8]	38.0
3	High body-mass index	4.8	[3.1, 6.6]	36.7
4	High blood pressure	4.4	[3.4, 5.5]	-20.1
5	Dietary risks	4.0	[3.4, 4.8]	-7.2
6	Occupational risks	2.3	[1.9, 2.7]	-36.1
7	High LDL	1.9	[1.4, 2.6]	-3.6
8	Malnutrition	1.7	[1.4, 2.0]	-11.7
9	Alcohol use	1.7	[1.1, 2.4]	17.9
10	Drug use	1.5	[1.3, 1.9]	44.7

Rank	Vestland	PAF	[95% UI]	Change (%)
1	Tobacco	6.0	[5.0, 7.0]	-31.5
2	High fasting plasma glucose	5.0	[3.8, 6.5]	45.0
3	High body-mass index	4.7	[3.0, 6.5]	42.6
4	High blood pressure	4.3	[3.3, 5.4]	-14.1
5	Dietary risks	4.0	[3.3, 4.8]	-7.1
6	Occupational risks	2.1	[1.8, 2.5]	-30.6
7	High LDL	1.9	[1.3, 2.5]	-2.1
8	Malnutrition	1.9	[1.5, 2.2]	-11.2
9	Alcohol use	1.6	[1.0, 2.3]	24.0
10	Drug use	1.5	[1.2, 1.8]	39.8

Rank	Viken	PAF	[95% UI]	Change (%)
1	Tobacco	6.5	[5.5, 7.5]	-29.3
2	High fasting plasma glucose	5.0	[3.9, 6.5]	39.6
3	High body-mass index	4.8	[3.1, 6.6]	31.4
4	High blood pressure	4.3	[3.4, 5.3]	-15.6
5	Dietary risks	3.9	[3.3, 4.7]	-6.8
6	Occupational risks	2.2	[1.9, 2.6]	-29.4
7	High LDL	1.8	[1.3, 2.5]	-3.5
8	Alcohol use	1.8	[1.2, 2.4]	17.6
9	Malnutrition	1.8	[1.5, 2.1]	-11.8
10	Drug use	1.6	[1.3, 1.9]	42.5

Behavioural risks
Environmental & occupational risks
Metabolic risks

Figure S13. Leading 10 level-2 risk factors for Norway and each Norwegian county by population attributable fraction (PAF) for all-cause DALY rate per 100 000 female inhabitants. Age-standardised. Change = percentage change in summary exposure variable (SEV) between 1990 and 2019. DALY = Disability-Adjusted Life Year.

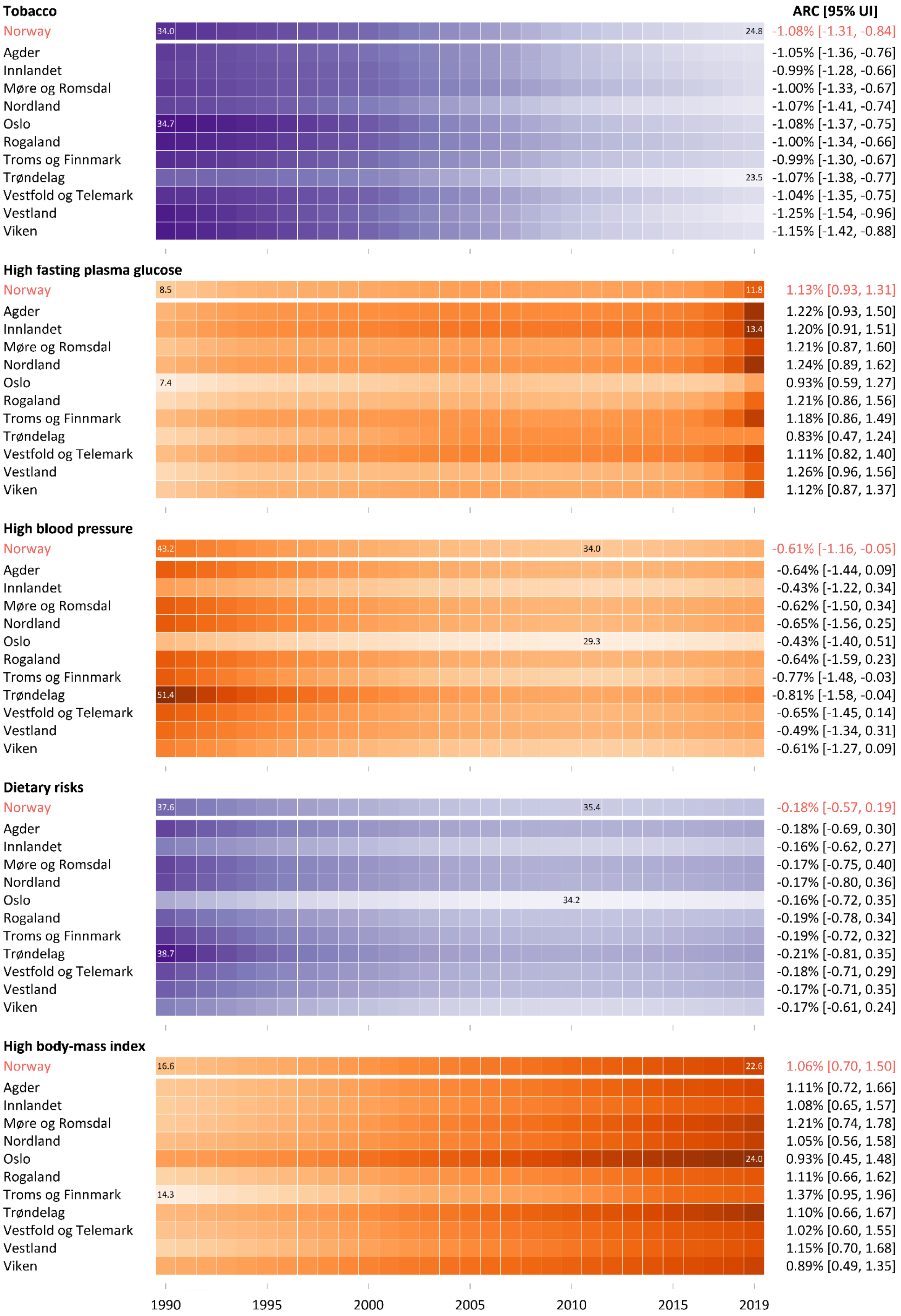

Figure S14. Exposure to the top-10 level-2 risk factors for disability-adjusted life years in Norway and each Norwegian county. Colour scale reflects age-adjusted summary exposure variable (SEV) values and is anchored in the minimum SEV (black labels) and maximum SEV (white labels) in any county between 1990 and 2019. ARC = Annualised rate of change. Purple panels = Behavioural risks, Green panels = Environmental and occupational risks, Orange panels = Metabolic risks

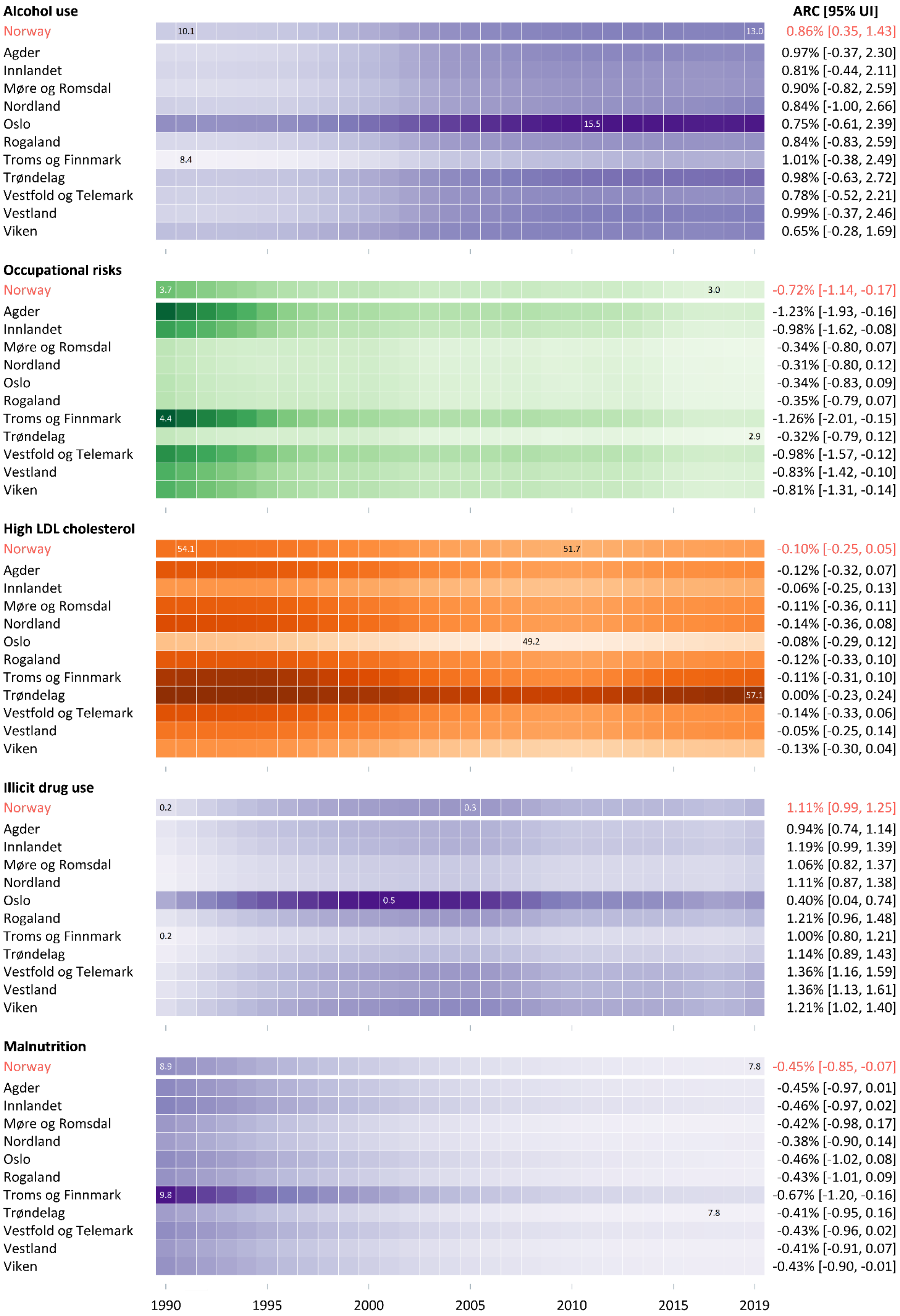

Figure S14 (cont.). Exposure to the top-10 level-2 risk factors for disability-adjusted life years in Norway and each Norwegian county. Colour scale reflects age-adjusted summary exposure variable (SEV) values and is anchored in the minimum SEV (black labels) and maximum SEV (white labels) in any county between 1990 and 2019. ARC = Annualised rate of change. Purple panels = Behavioural risks, Green panels = Environmental and occupational risks, Orange panels = Metabolic risks

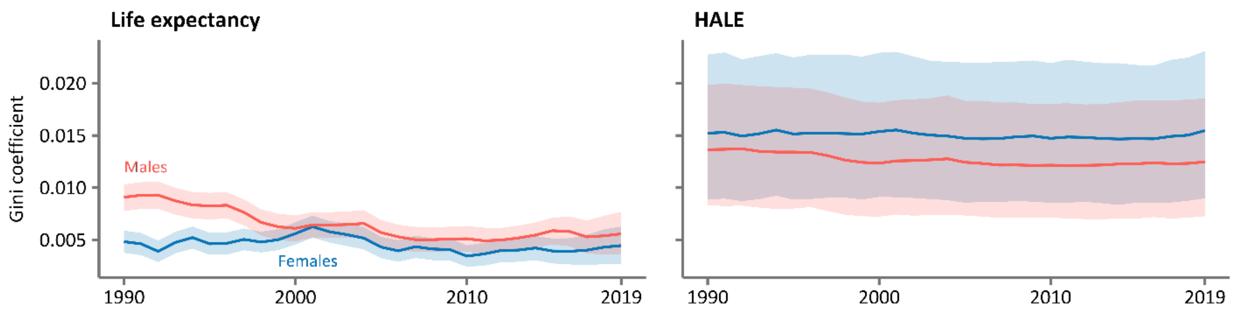

Figure S15a. Gini coefficient life expectancy and healthy life expectancy (HALE) across all Norwegian counties, 1990-2019. Shaded areas represent 95% uncertainty bands.

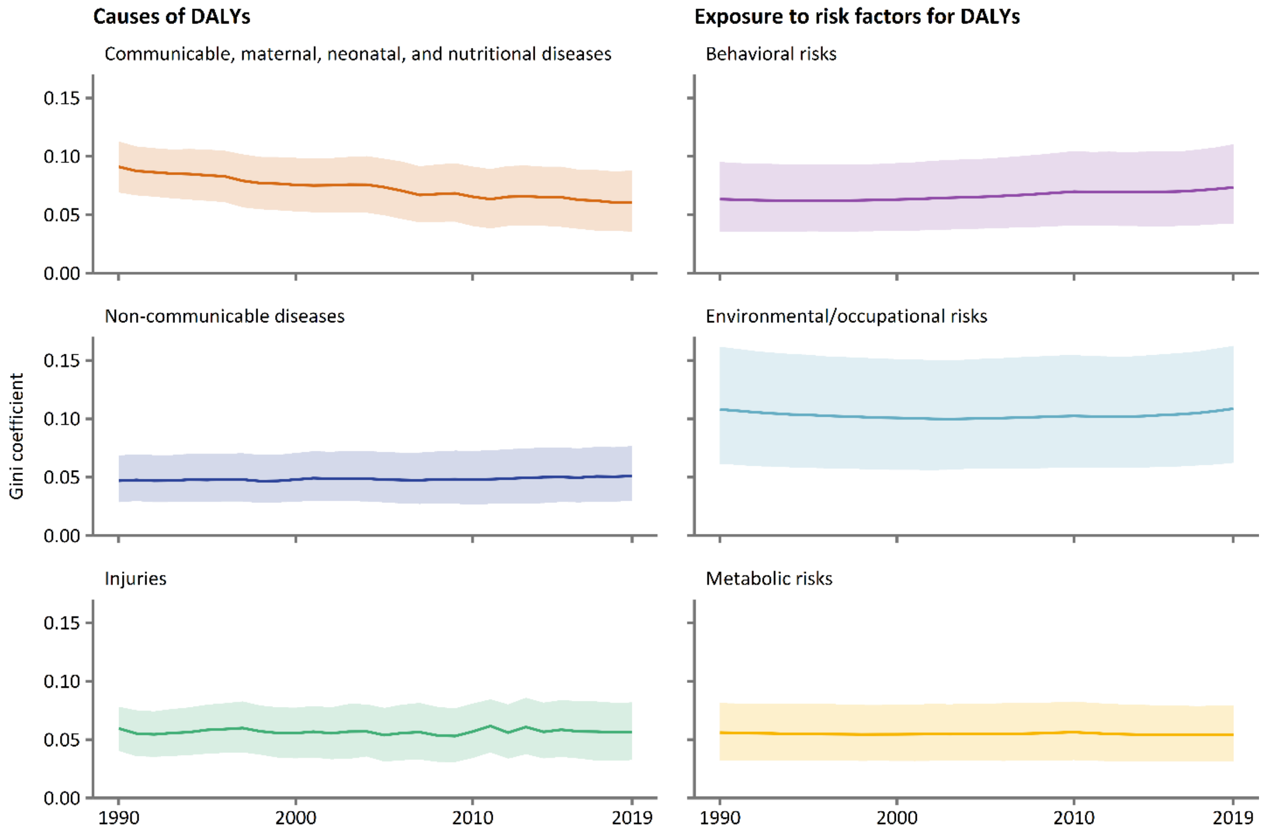

Figure S15b. Gini coefficient for level-1 causes of DALYs and level-1 risk factors for DALYS across all Norwegian counties, 1990-2019. Shaded areas represent 95% uncertainty bands.

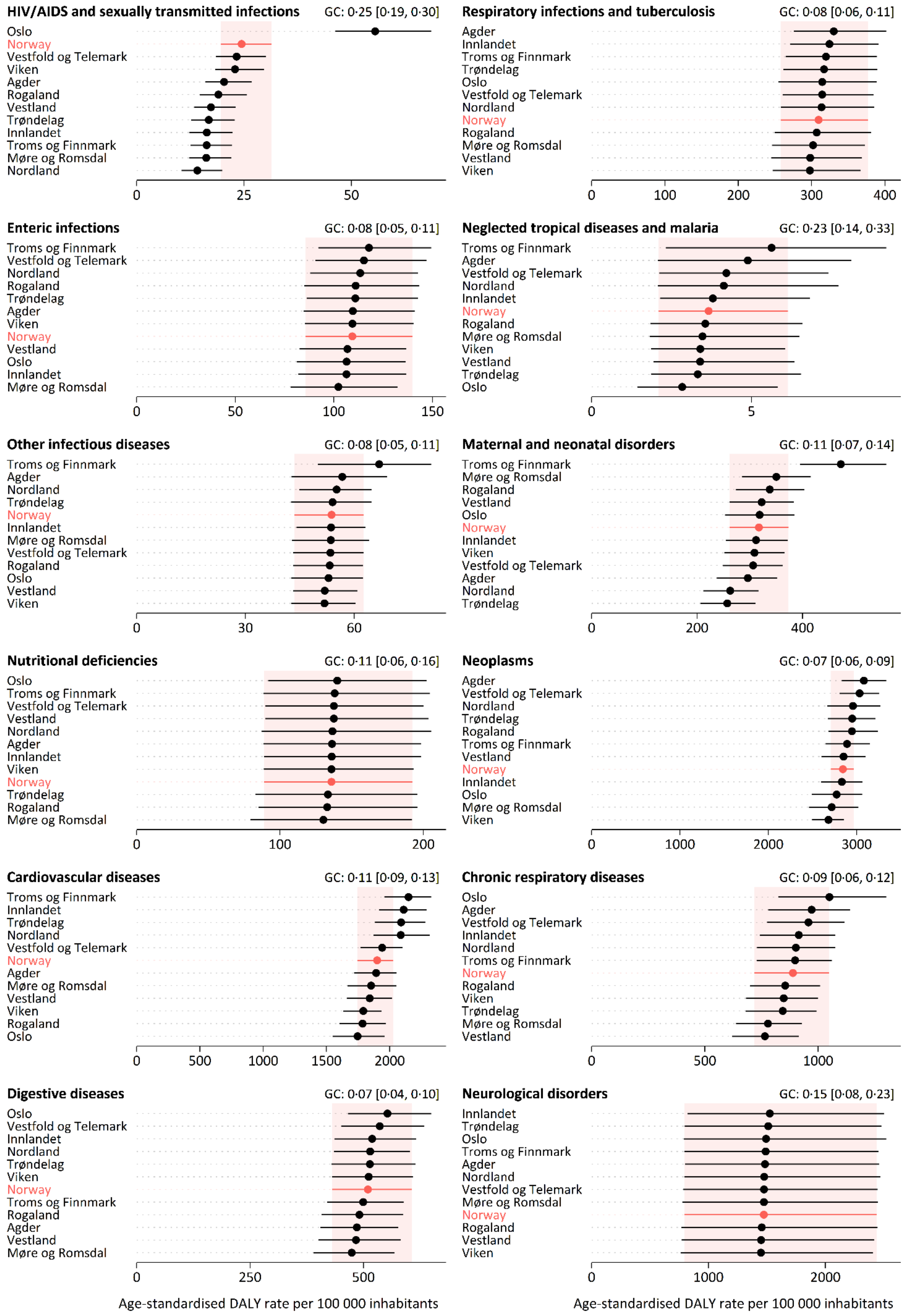

Figure S16. Gini coefficient and national and county-level estimates for level-2 cause group DALY rates in 2019. Shaded area represents the 95% uncertainty interval for the national YLL rate. GC = Gini coefficient and 95% uncertainty interval, DALY =Disability-Adjusted Life Year. Note: Gini coefficient estimates are higher when there is larger uncertainty in the underlying estimates

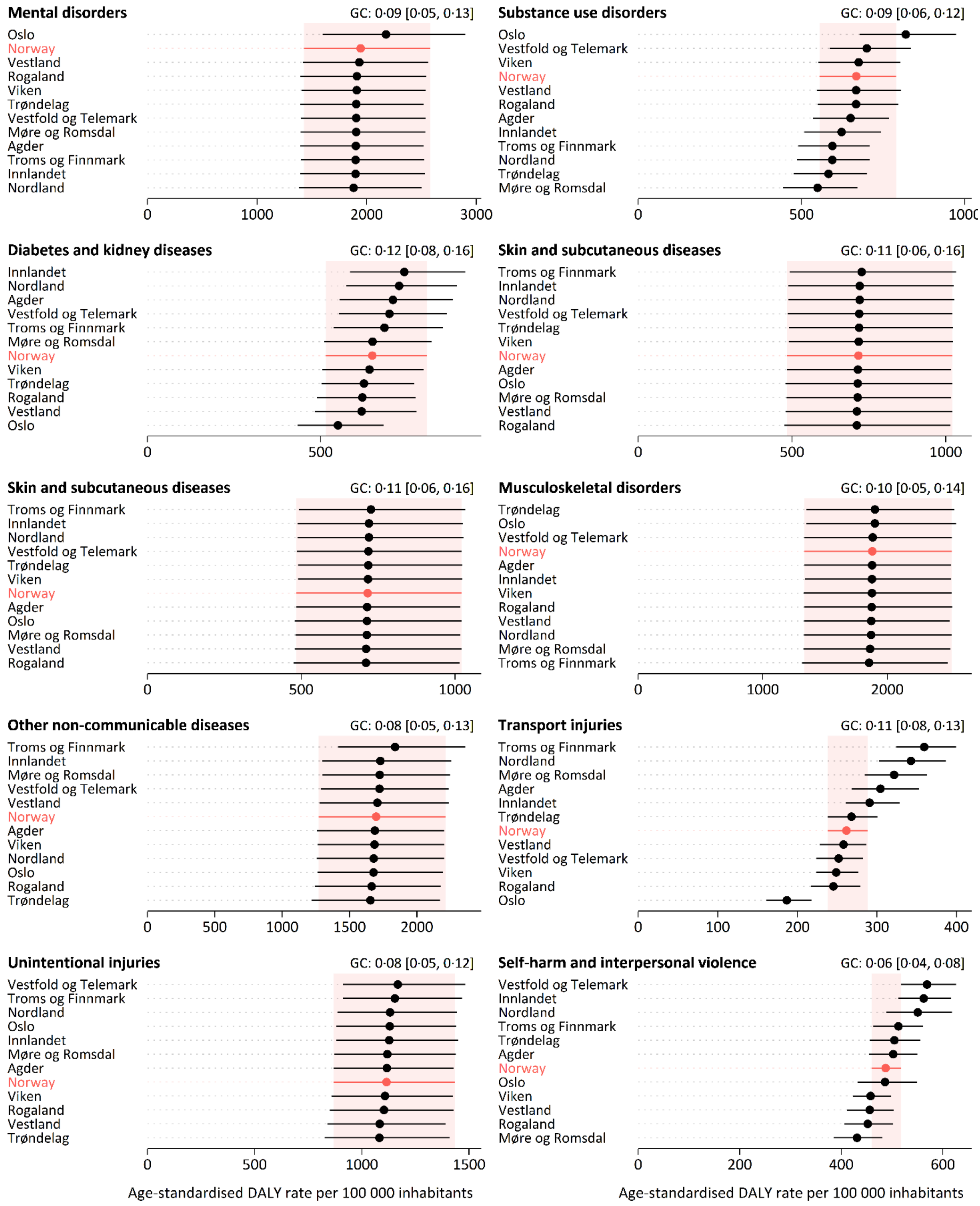

Figure S16 (cont.). Gini coefficient and national and county-level estimates for level-2 cause group DALY rates in 2019. Shaded area represents the 95% uncertainty interval for the national YLL rate. GC = Gini coefficient and 95% confidence interval, DALY =Disability-Adjusted Life Year. Note: Gini coefficient estimates are higher when there is larger uncertainty in the underlying estimates

Datasources non-fatal causes Norway GBD 2019

Aamodt G, Jahnsen J, Bengtson M-B, Moum B, Vatn MH, IBSEN Study Group. Geographic distribution and ecological studies of inflammatory bowel disease in southeastern Norway in 1990-1993. Inflamm Bowel Dis. 2008; 14(7): 984–91

Aamodt G, Stene LC, Njolstad PR, Sovik O, Joner G. Spatiotemporal trends and age-period-cohort modeling of the incidence of type 1 diabetes among children aged

Aarhus University, Addiction Switzerland Research Institute, Alcohol Research Group, Public Health Institute, Centre for Addiction and Mental Health (Canada), Centre for Alcohol Policy Research, Turning Point Alcohol and Drug Centre (Australia), Kettil Bruun Society for Social and Epidemiological Research on Alcohol, University of North Dakota. Norway Gender, Alcohol and Culture: An International Study (GENACIS) 1999

Ahmad AS, Samuelsen SO. Hypertensive disorders in pregnancy and fetal death at different gestational lengths: a population study of 2 121 371 pregnancies. BJOG. 2012; 119(12): 1521-8

Airbase European Air Quality Database Version 8, 2014 as it appears in Shaddick G, Thomas ML. Particulate Matter 2.5 and 10 Surface Monitor Station Expanded Database 2008-2017. [Unpublished]

Almquist M, Johansen D, Björge T, Ulmer H, Lindkvist B, Stocks T, Hallmans G, Engeland A, Rapp K, Jonsson H, Selmer R, Diem G, Häggström C, Tretli S, Stattin P, Manjer J. Metabolic factors and risk of thyroid cancer in the Metabolic syndrome and Cancer project (Me-Can). Cancer Causes Control. 2011; 22(5): 743–51

Amundsen E, Bretteville-Jensen A. Hard drug use in Norway. Nordisk Alkohol Nark. 2010; 27(1): 87–94

Andersgaard AB, Langhoff-Roos J, Øian P. Direct maternal deaths in Norway 1976-1995. Acta Obstet Gynecol Scand. 2008; 87(8): 856-61

Askim Å, Gustad LT, Paulsen J, Reitan SK, Mehl A, Mohus RM, Dewan A, Damås JK, Solligård E, Åsvold BO. Anxiety and Depression Symptoms in a General Population and Future Risk of Bloodstream Infection: The HUNT Study. Psychosom Med. 2018; 80(7): 673-679

Association of Nordic Cancer Registries (ANCR). Norway NORDCAN Cancer Incidence and Mortality Data Tables, Age-Specific by Countries. Copenhagen, Denmark: Association of Nordic Cancer Registries (ANCR)

Association of Nordic Cancer Registries (ANCR). Norway NORDCAN Cancer Incidence Data Tables, Age-Specific by Countries. Copenhagen, Denmark: Association of Nordic Cancer Registries (ANCR)

Association of Nordic Cancer Registries (ANCR). Norway NORDCAN Cancer Mortality Data Tables, Age-Specific by Countries. Copenhagen, Denmark: Association of Nordic Cancer Registries (ANCR)

Ayuso-Mateos JL, Vázquez-Barquero JL, Dowrick C, Lehtinen V, Dalgard OS, Casey P, Wilkinson C, Lasa L, Page H, Dunn G, Wilkinson G. Depressive disorders in Europe: prevalence figures from the ODIN study. Br J Psychiatry. 2001; 179(4): 308-16

Bakken KS, Skjeldal OH, Stray-Pedersen B. Higher risk for adverse obstetric outcomes among immigrants of African and Asian descent: a comparison study at a low-risk maternity hospital in Norway. Birth. 2015; 42(2): 132-40

Bakken T, Braaten T, Olsen A, Kyrø C, Lund E, Skeie G. Consumption of Whole-Grain Bread and Risk of Colorectal Cancer among Norwegian Women (the NOWAC Study). Nutrients. 2016; 8(1)

Bauman A, Bull F, Chey T, Craig CL, Ainsworth BE, Sallis JF, Bowles HR, Hagstromer M, Sjostrom M, Pratt M, IPS Group. The International Prevalence Study on Physical Activity: results from 20 countries. Int J Behav Nutr Phys Act. 2009; 21

Bengtson MB, Solberg C, Aamodt G, Sauar J, Jahnsen J, Moum B, Lygren I, Vatn MH, IBSEN study group. Familial aggregation in Crohn's disease and ulcerative colitis in a Norwegian population-based cohort followed for ten years. J Crohns Colitis. 2009; 3(2): 92-9

Bere E, Westersjo JH. Nature trips and traditional methods for food procurement in relation to weight status. Scand J Public Health. 2013; 41(2): 180-4

Berg AS, Inchley CS, Aase A, Fjaerli HO, Bull R, Aaberge I, Leegaard TM, Nakstad B. Etiology of Pneumonia in a Pediatric Population with High Pneumococcal Vaccine Coverage: A Prospective Study. Pediatr Infect Dis J. 2016; 35(3): e69-75

Bingham SA, Norat T, Moskal A, Ferrari P, Slimani N, Clavel-Chapelon F, Kesse E, Nieters A, Boeing H, Tjønneland A, Overvad K, Martinez C, Dorronsoro M, González CA, Ardanaz E, Navarro C, Quirós JR, Key TJ, Day NE, Trichopoulou A, Naska A, Krogh V, Tumino R, Palli D, Panico S, Vineis P, Bueno-de-Mesquita HB, Ocké MC, Peeters PHM, Berglund G, Hallmans G, Lund E, Skeie G, Kaaks R, Riboli E. Is the association with fiber from foods in colorectal cancer confounded by folate intake?. Cancer Epidemiol Biomark Prev. 2005; 14(6): 1552–6

Birkeland JM, Haugejorden O, von der Fehr FR. Analyses of the caries decline and incidence among Norwegian adolescents 1985-2000. Acta Odontol Scand. 2002; 60(5): 281-9

Bjartveit K, Tverdal A. Health consequences of smoking 1-4 cigarettes per day. Tob Control. 2005; 14(5): 315–20

Bjelland M, Bergh IH, Grydeland M, Klepp K-I, Andersen LF, Anderssen SA, Ommundsen Y, Lien N. Changes in adolescents' intake of sugar-sweetened beverages and sedentary behaviour: results at 8 month mid-way assessment of the HEIA study--a comprehensive, multi-component school-based randomized trial. Int J Behav Nutr Phys Act. 2011; 63

Bjørge T, Tretli S, Engeland A. Relation of height and body mass index to renal cell carcinoma in two million Norwegian men and women. Am J Epidemiol. 2004; 160(12): 1168–76

Bo K, Thoresen M, Dalgard F. Smokers report more psoriasis, but not atopic dermatitis or hand eczema: results from a Norwegian population survey among adults. Dermatology (Basel). 2008; 216(1): 40–5

Borch KB, Lund E, Braaten T, Weiderpass E. Physical activity and the risk of postmenopausal breast cancer - the Norwegian Women and Cancer Study. J Negat Results Biomed. 2014; 13: 3

Borch-Iohnsen B, Sandstad B, Asberg A. Iron status among 3005 women aged 20-55 years in Central Norway: the Nord-Trøndelag Health Study (the HUNT study). Scand J Clin Lab Invest. 2005; 65(1): 45-54

Bovim G, Schrader H, Sand T. Neck pain in the general population. Spine. 1994; 19(12): 1307-9

Braathen LR, Botten G, Bjerkedal T. Psoriatics in Norway. A questionnaire study on health status, contact with paramedical professions, and alcohol and tobacco consumption. Acta Derm Venereol Suppl (Stockh). 1989; 142: 9–12

Brage S, Laerum E. Spinal disorders in Norway – an epidemiological report. Tidsskr Nor Laegeforen. 1999; 119(11): 1619-23

Brannsether B, Eide GE, Roelants M, Bjerknes R, Júlíusson PB. Interrelationships between anthropometric variables and overweight in childhood and adolescence. Am J Hum Biol. 2014; 26(4): 502–10

Bray F, Colombet M, Mery L, Piñeros M, Znaor A, Zanetti R and Ferlay J, editors (2017). Cancer Incidence in Five Continents, Vol. XI (electronic version). Lyon: International Agency for Research on Cancer. Available from: http://ci5.iarc.fr

Brevik A, Vollset SE, Tell GS, Refsum H, Ueland PM, Loeken EB, Drevon CA, Andersen LF. Plasma concentration of folate as a biomarker for the intake of fruit and vegetables: the Hordaland Homocysteine Study. Am J Clin Nutr. 2005; 81(2): 434–9

Brunes A. The Association Between Physical Activity, Mental Health, and Personality: The HUNT Study [master's thesis]. [Trondheim, Norway]: Norwegian University of Science and Technology; 2011

Brustad M, Skeie G, Braaten T, Slimani N, Lund E. Comparison of telephone versus face-to-face interviews in the assessment of dietary intake by the 24-hour recall EPIC SOFT programme--the Norwegian calibration study. IARC Sci Publ. 2002; 156: 17-9

Brustad M, Skeie G, Braaten T, Slimani N, Lund E. Comparison of telephone vs face-to-face interviews in the assessment of dietary intake by the 24 h recall EPIC SOFT program--the Norwegian calibration study. Eur J Clin Nutr. 2003; 57(1): 107-13

Buist AS, McBurnie MA, Vollmer WM, Gillespie S, Burney P, Mannino DM, Menezes AM, Sullivan SD, Lee TA, Weiss KB, Jensen RL, Marks GB, Gulsvik A,Nizankowska-Mogilnicka E, BOLD Collaborative Research Group.

International variation in the prevalence of COPD (the BOLD Study): a population-based prevalence study. Lancet. 2007; 9589(9589): 741-50

Carlsson S, Midthjell K, Tesfamarian MY, Grill V. Age, overweight and physical inactivity increase the risk of latent autoimmune diabetes in adults: results from the Nord-Trøndelag health study. Diabetologia. 2007; 50(1): 55-8

Cattaneo A. Breastfeeding in Europe: a blueprint for action. J Public Health. 2005; 13(2): 89-96

Centre for Reading Research, Stavanger (Norway), Educational Testing Service, National Center for Education Statistics (USA), Organization for Economic Co-operation and Development (OECD), Statistics Canada, United Nations Educational, Scientific and Cultural Organization (UNESCO). Norway Adult Literacy and Life Skills Survey 2003. Ottawa, Canada: Statistics Canada

Chitsulo L, Engels D, Montresor A, Savioli L. The global status of schistosomiasis and its control. Acta Trop. 2000; 77(1): 41-51

Chronic Kidney Disease Prognosis Consortium (CKD-PC). Norway Tromso Study Chronic Kidney Disease Prevalence Data 1994-1995 [Unpublished]

Cibula D. Women's contraceptive practices and sexual behavior in Europe report. Eur J Contracept Reprod Health Care. 2008; 13(4): 362-375

Clausen T, Anchersen K, Waal H. Mortality prior to, during and after opioid maintenance treatment (OMT): a national prospective cross-registry study. Drug Alcohol Depend. 2008; 94(1-3): 151-7

Commission of the European Communities (2012): Eurobarometer 34.0 (Oct-Nov 1990). INRA, Brussels. GESIS Data Archive, Cologne. ZA1960 Data file Version 1.0.1, doi:10.4232/1.10892

Commission of the European Communities (2012): Eurobarometer 36 (Oct-Nov 1991). INRA, Brussels. GESIS Data Archive, Cologne. ZA2081 Data file Version 1.1.0, doi:10.4232/1.10848

Commission of the European Communities (2012): Eurobarometer 37.0+37.1 (Mar-May 1992). INRA, Brussels. GESIS Data Archive, Cologne. ZA2243 Data file Version 1.1.0, doi:10.4232/1.10900

Commission of the European Communities (2012): Eurobarometer 38.0 (Sep-Oct 1992). INRA, Brussels. GESIS Data Archive, Cologne. ZA2294 Data file Version 1.1.0, doi:10.4232/1.10903

Commission of the European Communities (2012): Eurobarometer 39.0 (Mar-Apr 1993). INRA, Brussels. GESIS Data Archive, Cologne. ZA2346 Data file Version 1.1.0, doi:10.4232/1.10905

Commission of the European Communities (2012): Eurobarometer 39.1 (May-Jun 1993). INRA, Brussels. GESIS Data Archive, Cologne. ZA2347 Data file Version 1.1.0, doi:10.4232/1.10906

Curado MP, Edwards B, Shin HR, Storm H, Ferlay J, Heanue M and Boyle P, eds (2007). Cancer Incidence in Five Continents, Vol. IX Periodic Data (electronic version). Lyon, IARC. http://ci5.iarc.fr

Cystic Echinococcosis Endemicity Estimates identified through systematic review and personal communication, as provided by GBD 2015 expert. [Unpublished]

de Marco R, Accordini S, Cerveri I, Corsico A, Sunyer J, Neukirch F, Künzli N, Leynaert B, Janson C, Gislason T, Vermeire P, Svanes C, Anto JM, Burney P, European Community Respiratory Health Survey Study Group. An international survey of chronic obstructive pulmonary disease in young adults according to GOLD stages. Thorax. 2004; 59(2): 120-125

Demography of dialysis and transplantation in Europe in 1985 and 1986: trends over the previous decade. Report from the European Dialysis and Transplant Association Registry

Diaz E, Kumar BN, Engedal K. Immigrant patients with dementia and memory impairment in primary health care in Norway: a national registry study. Dement Geriatr Cogn Disord. 2015; 39(5-6): 321–31

Directorate of Health (Iceland), National Board of Health and Welfare (Sweden), National Institute for Health and Welfare (THL) (Finland), Norwegian Institute of Public Health, Statens Serum Institute, Statistics Denmark. Induced Abortions in the Nordic Countries 2013. Helsinki, Finland: National Institute for Health and Welfare (THL) (Finland), 2015

Dirren H, Decarli B, Lesourd B, Schlienger JL, Deslypere JP, Kiepurski A. Nutritional status: haematology and albumin. Eur J Clin Nutr. 1991; 45 (Suppl 3): 43-52

Due P, Holstein BE, Lynch J, Diderichsen F, Gabhain SN, Scheidt P, Currie C. Bullying and symptoms among school-aged children: international comparative cross sectional study in 28 countries. Eur J Public Health. 2005; 15(2): 128–32

Dypvik J, Strom-Roum EM, Haavaldsen C, Vatten LJ, Eskild A. Preeclampsia in pregnancies with and without diabetes: the associations with placental weight. A population study of 655 842 pregnancies. Acta Obstet Gynecol Scand. 2016; 95(2): 217-24

EEA Air Quality e-Reporting Database 2017 as it appears in Shaddick G, Thomas ML. Particulate Matter 2.5 and 10 Surface Monitor Station Expanded Database 2008-2017. [Unpublished]

EEA Air Quality e-Reporting Database 2018 as it appears in Shaddick G, Thomas ML. Particulate Matter 2.5 and 10 Surface Monitor Station Expanded Database 2008-2017. [Unpublished]

Egan KB, Ettinger AS, DeWan AT, Holford TR, Holmen TL, Bracken MB. General, but not abdominal, overweight increases odds of asthma among Norwegian adolescents: the Young-HUNT study. Acta Paediatr. 2014; 103(12): 1270–6

Einvik G, Klemsdal TO, Sandvik L, Hjerkinn EM. A randomized clinical trial on n-3 polyunsaturated fatty acids supplementation and all-cause mortality in elderly men at high cardiovascular risk. Eur J Cardiovasc Prev Rehabil. 2010; 17(5): 588-92

Elgen SK, Leversen KT, Grundt JH, Hurum J, Sundby AB, Elgen IB, Markestad T. Mental health at 5 years among children born extremely preterm: a national population-based study. Eur Child Adolesc Psychiatry. 2012; 21(10): 583-9

Ellekjaer H, Holmen J, Ellekjaer E, Vatten L. Physical activity and stroke mortality in women. Ten-year follow-up of the Nord-Trondelag health survey, 1984-1986. Stroke. 2000; 31(1): 14-8

Engdahl B, Tambs K, Borchgrevink HM, Hoffman HJ. Screened and unscreened hearing threshold levels for the adult population: results from the Nord-Trøndelag Hearing Loss Study [Unpublished data]. Int J Audiol. 2005; 44(4): 213-30

Engeland A, Haldorsen T, Andersen A, Tretli S. The impact of smoking habits on lung cancer risk: 28 years' observation of 26,000 Norwegian men and women. Cancer Causes Control. 1996; 7(3): 366–76

Engeland A, Tretli S, Akslen LA, Bjørge T. Body size and thyroid cancer in two million Norwegian men and women. Br J Cancer. 2006; 95(3): 366–70

Engeland A, Tretli S, Bjørge T. Height and body mass index in relation to esophageal cancer; 23-year follow-up of two million Norwegian men and women. Cancer Causes Control. 2004; 15(8): 837–43

Engeland A, Tretli S, Hansen S, Bjørge T. Height and body mass index and risk of lymphohematopoietic malignancies in two million Norwegian men and women. Am J Epidemiol. 2007; 165(1): 44–52

Eritsland J, Arnesen H, Grønseth K, Fjeld NB, Abdelnoor M. Effect of dietary supplementation with n-3 fatty acids on coronary artery bypass graft patency. Am J Cardiol. 1996; 77(1): 31-6

Eskeland B, Hunskaar S. Anaemia and iron deficiency screening in adolescence: a pilot study of iron stores and haemoglobin response to iron treatment in a population of 14-15-year-olds in Norway. Acta Paediatr. 1999; 88(8): 815-21

Eskild A, Jeansson S, Jenum PA. [Antibodies against Herpes simplex virus type 2 among pregnant women in Norway]. Tidsskr Nor Laegeforen. 1999; 119(16): 2323-6

Eskild A, Magnus P, Samuelsen SO, Sohlberg C, Kittelsen P. Differences in mortality rates and causes of death between HIV positive and HIV negative intravenous drug users. Int J Epidemiol. 1993; 22(2): 315-20

Euromonitor International. Euromonitor Passport - Alcoholic Drinks Statistics . London, United Kingdom: Euromonitor International

Euromonitor International. Euromonitor Passport - Fresh Foods Market Statistics. London, United Kingdom: Euromonitor International

Euromonitor International. Euromonitor Passport - Smoking Tobacco Statistics . London, United Kingdom: Euromonitor International

European Centre for Disease Prevention and Control, World Health Organization Regional Office for Europe (WHO/Europe). HIV/AIDS Surveillance in Europe 2012. Stockholm, Sweden: European Centre for Disease Prevention and Control, 2013

European Centre for Disease Prevention and Control, World Health Organization Regional Office for Europe (WHO/Europe). HIV/AIDS Surveillance in Europe 2013. Stockholm, Sweden: European Centre for Disease Prevention and Control, 2014

European Centre for Disease Prevention and Control, World Health Organization Regional Office for Europe (WHO/Europe). HIV/AIDS Surveillance in Europe 2014. Stockholm, Sweden: European Centre for Disease Prevention and Control, 2015

European Centre for Disease Prevention and Control, World Health Organization Regional Office for Europe (WHO/Europe). HIV/AIDS Surveillance in Europe 2015. Stockholm, Sweden: European Centre for Disease Prevention and Control, 2016

European Centre for Disease Prevention and Control, World Health Organization Regional Office for Europe (WHO/Europe). HIV/AIDS Surveillance in Europe 2018 - 2017 Data. Stockholm, Sweden: European Centre for Disease Prevention and Control, 2018

European Centre for Disease Prevention and Control. ECDC Surveillance Atlas of Infectious Diseases - Zika Virus Infection. Stockholm, Sweden: European Centre for Disease Prevention and Control

European Centre for Disease Prevention and Control. Review of Chlamydia Control Activities in EU Countries. Stockholm, Sweden: European Centre for Disease Prevention and Control, 2008

European Commission (2012): Eurobarometer 40 (Oct-Nov 1993). INRA, Brussels. GESIS Data Archive, Cologne. ZA2459 Data file Version 1.1.0, doi:10.4232/1.10908

European Commission (2012): Eurobarometer 41.0 (Mar-May 1994). INRA, Brussels. GESIS Data Archive, Cologne. ZA2490 Data file Version 1.1.0, doi:10.4232/1.10909

European Commission (2012): Eurobarometer 42 (Nov-Dec 1994). INRA, Brussels. GESIS Data Archive, Cologne. ZA2563 Data file Version 1.0.1, doi:10.4232/1.10911

European Commission (2012): Eurobarometer 43.1 (Apr-May 1995). INRA, Brussels. GESIS Data Archive, Cologne. ZA2637 Data file Version 1.0.1, doi:10.4232/1.10913

European Commission (2012): Eurobarometer 44.1 (Nov-Dec 1995). INRA, Brussels. GESIS Data Archive, Cologne. ZA2690 Data file Version 1.0.1, doi:10.4232/1.10917

European Commission (2012): Eurobarometer 46.1 (Oct-Nov 1996). INRA, Brussels. GESIS Data Archive, Cologne. ZA2899 Data file Version 1.1.0, doi:10.4232/1.10924

European Commission (2012): Eurobarometer 52.1 (Nov-Dec 1999). INRA, Brussels; NSD, Bergen. GESIS Data Archive, Cologne. ZA3205 Data file Version 2.0.0, doi:10.4232/1.11377

European Commission (2012): Eurobarometer 54.1 (Oct-Nov 2000). European Opinion Research Group (EORG), Brussels. GESIS Data Archive, Cologne. ZA3387 Data file Version 1.1.0, doi:10.4232/1.10937

European Commission (2012): Eurobarometer 54.2 (Jan-Feb 2001). European Opinion Research Group (EORG), Brussels. GESIS Data Archive, Cologne. ZA3388 Data file Version 1.1.0, doi:10.4232/1.10938

European Commission (2012): Eurobarometer 56.1 (Sept-Oct 2001). European Opinion Research Group (EORG), Brussels; NSD, Bergen. GESIS Data Archive, Cologne. ZA3626 Data file Version 2.0.0, doi:10.4232/1.11378

European Commission (2012): Eurobarometer 57.2OVR (Apr-Jun 2002). European Opinion Research Group (EORG), Brussels. GESIS Data Archive, Cologne. ZA3641 Data file Version 1.0.1, doi:10.4232/1.10951

European Commission (2012): Eurobarometer 59.0 (Jan-Feb 2003). European Opinion Research Group (EORG), Brussels. GESIS Data Archive, Cologne. ZA3903 Data file Version 1.0.1, doi:10.4232/1.11352

European Commission (2012): Eurobarometer 63.1 (Jan-Feb 2005). TNS OPINION & SOCIAL, Brussels [Producer]. GESIS Data Archive, Cologne. ZA4233 Data file Version 1.1.0, doi:10.4232/1.10965

European Commission (2012): Eurobarometer 73.2+73.3 (2-3 2010). GESIS Data Archive, Cologne. ZA5236 Data file Version 2.0.1, doi:10.4232/1.11473

European Commission (2012): Eurobarometer 76.2 (2011). TNS OPINION & SOCIAL, Brussels [Producer]. GESIS Data Archive, Cologne. ZA5566 Data file Version 1.0.0, doi:10.4232/1.11388

European Commission and European Parliament, Brussels (2012): Eurobarometer 73.1 (Jan-Feb 2010). TNS OPINION & SOCIAL, Brussels [Producer]. GESIS Data Archive, Cologne. ZA5000 Data file Version 4.0.0, doi:10.4232/1.11428

European Community Respiratory Health Survey Study Group. European Community Respiratory Health Survey 1992-2003 as provided by the Global Burden of Disease 2010 Respiratory Diseases Expert Group. [Unpublished]

European Monitoring Centre for Drugs and Drug Addiction (EMCDDA), European School Survey Project on Alcohol and Other Drugs (ESPAD), Pompidou Group, Council of Europe, Swedish Council for Information on Alcohol and Other Drugs (CAN). ESPAD Report 2007: Substance Use Among Students in 35 European Countries. Stockholm, Sweden: Swedish Council for Information on Alcohol and Other Drugs (CAN), 2009

European Monitoring Centre for Drugs and Drug Addiction (EMCDDA), European School Survey Project on Alcohol and Other Drugs (ESPAD), Pompidou Group, Council of Europe, Swedish Council for Information on Alcohol and Other Drugs (CAN). ESPAD Report 2011: Substance Use Among Students in 36 European Countries. Stockholm, Sweden: Swedish Council for Information on Alcohol and Other Drugs (CAN), 2012

European Monitoring Centre for Drugs and Drug Addiction (EMCDDA). EMCDDA Annual Report 2007. Lisbon, Portugal: European Monitoring Centre for Drugs and Drug Addiction (EMCDDA), 2007

European Monitoring Centre for Drugs and Drug Addiction (EMCDDA). ESPAD Report 2015: Results from the European School Survey Project on Alcohol and Other Drugs. Lisbon, Portugal: European School Survey Project on Alcohol and Other Drugs (ESPAD), 2016

European Monitoring Centre for Drugs and Drug Addiction (EMCDDA). European Monitoring Centre for Drugs and Drug Addiction Statistical Bulletin 2008. Lisbon, Portugal: European Monitoring Centre for Drugs and Drug Addiction (EMCDDA)

European Monitoring Centre for Drugs and Drug Addiction (EMCDDA). European Monitoring Centre for Drugs and Drug Addiction Statistical Bulletin 2013. Lisbon, Portugal: European Monitoring Centre for Drugs and Drug Addiction (EMCDDA)

European Monitoring Centre for Drugs and Drug Addiction (EMCDDA). European Monitoring Centre for Drugs and Drug Addiction Statistical Bulletin 2015. Lisbon, Portugal: European Monitoring Centre for Drugs and Drug Addiction (EMCDDA)

European Monitoring Centre for Drugs and Drug Addiction (EMCDDA). European Monitoring Centre for Drugs and Drug Addiction Statistical Bulletin 2016. Lisbon, Portugal: European Monitoring Centre for Drugs and Drug Addiction (EMCDDA), 2016

European School Survey Project on Alcohol and Other Drugs (ESPAD), Pompidou Group, Council of Europe, Swedish Council for Information on Alcohol and Other Drugs (CAN). ESPAD Report 2003: Alcohol and Other Drug Use Among Students in 35 European Countries. Stockholm, Sweden: Swedish Council for Information on Alcohol and Other Drugs (CAN), 2004

EURO-PERISTAT, EuroNeoStat, European Surveillance of Congenital Anomalies (EUROCAT), Netherlands Organisation for Applied Scientific Research (TNO), Surveillance of Cerebral Palsy in Europe (SCPE). European Perinatal Health Report 2004. EURO-PERISTAT, 2008

Eurostat. Norway Labor Force Survey 1995 - Eurostat. Luxembourg, Luxembourg: Eurostat, 2017

Eurostat. Norway Labor Force Survey 1996 - Eurostat. Luxembourg, Luxembourg: Eurostat, 2017

Eurostat. Norway Labor Force Survey 1997 - Eurostat. Luxembourg, Luxembourg: Eurostat, 2017

Eurostat. Norway Labor Force Survey 1998 - Eurostat. Luxembourg, Luxembourg: Eurostat, 2017

Eurostat. Norway Labor Force Survey 1999 - Eurostat. Luxembourg, Luxembourg: Eurostat, 2017

Eurostat. Norway Labor Force Survey 2000 - Eurostat. Luxembourg, Luxembourg: Eurostat, 2017

Eurostat. Norway Labor Force Survey 2001 - Eurostat. Luxembourg, Luxembourg: Eurostat, 2017

Eurostat. Norway Labor Force Survey 2002 - Eurostat. Luxembourg, Luxembourg: Eurostat, 2017

Eurostat. Norway Labor Force Survey 2003 - Eurostat. Luxembourg, Luxembourg: Eurostat, 2017

Eurostat. Norway Labor Force Survey 2004 - Eurostat. Luxembourg, Luxembourg: Eurostat, 2017

Eurostat. Norway Labor Force Survey 2005 - Eurostat. Luxembourg, Luxembourg: Eurostat, 2017

Eurostat. Norway Labor Force Survey 2006 - Eurostat. Luxembourg, Luxembourg: Eurostat, 2017

Eurostat. Norway Labor Force Survey 2007 - Eurostat. Luxembourg, Luxembourg: Eurostat, 2017

Eurostat. Norway Labor Force Survey 2008 - Eurostat. Luxembourg, Luxembourg: Eurostat, 2017

Eurostat. Norway Labor Force Survey 2009 - Eurostat. Luxembourg, Luxembourg: Eurostat, 2017

Eurostat. Norway Labor Force Survey 2010 - Eurostat. Luxembourg, Luxembourg: Eurostat, 2017

Eurostat. Norway Labor Force Survey 2011 - Eurostat. Luxembourg, Luxembourg: Eurostat, 2017

Eurostat. Norway Labor Force Survey 2012 - Eurostat. Luxembourg, Luxembourg: Eurostat, 2017

Eurostat. Norway Labor Force Survey 2013 - Eurostat. Luxembourg, Luxembourg: Eurostat, 2017

Eurostat. Norway Labor Force Survey 2014 - Eurostat. Luxembourg, Luxembourg: Eurostat, 2017

Eurostat. Norway Labor Force Survey 2015 - Eurostat. Luxembourg, Luxembourg: Eurostat, 2017

Eurostat. Norway Labor Force Survey 2016 - Eurostat. Luxembourg, Luxembourg: Eurostat, 2017

EUVAC-NET. EUVAC-NET Measles Surveillance Report 2010. Stockholm, Sweden: European Centre for Disease Prevention and Control, 2011

EUVAC-NET. EUVAC-NET Pertussis Surveillance Report 1998-2002. Stockholm, Sweden: European Centre for Disease Prevention and Control

EUVAC-NET. EUVAC-NET Pertussis Surveillance Report 2003-2007. Stockholm, Sweden: European Centre for Disease Prevention and Control, 2009

EUVAC-NET. EUVAC-NET Pertussis Surveillance Report 2008. Stockholm, Sweden: European Centre for Disease Prevention and Control, 2009

EUVAC-NET. EUVAC-NET Pertussis Surveillance Report 2009. Stockholm, Sweden: European Centre for Disease Prevention and Control, 2010

Evensen S, Wisløff T, Lystad JU, Bull H, Ueland T, Falkum E. Prevalence, Employment Rate, and Cost of Schizophrenia in a High-Income Welfare Society: A Population-Based Study Using Comprehensive Health and Welfare Registers. Schizophr Bull. 2016; 42(2): 476–83

Faber MT, Nielsen A, Nygård M, Sparén P, Tryggvadottir L, Hansen BT, Liaw K-L, Kjaer SK. Genital chlamydia, genital herpes, Trichomonas vaginalis and gonorrhea prevalence, and risk factors among nearly 70,000 randomly selected women in 4 Nordic countries. Sex Transm Dis. 2011; 38(8): 727-34

FAO Supply Utilization Accounts 1961-2013. Personal Correspondence with Dr. Josef Schmidhuber, 2016. [Unpublished]

Farbu E, Rudolph T, Stefansdottir S. Guillain Barre syndrome--Incidence and clinical course in Southern Rogaland, Norway. Clin Neurol Neurosurg. 2016; 141: 33-7

Feenstra, Robert C., Robert Inklaar and Marcel P. Timmer (2015), "The Next Generation of the Penn World Table" American Economic Review, 105(10), 3150-3182, available for download at www.ggdc.net/pwt

Ferlay J, Parkin DM, Curado MP, Bray F, Edwards B, Shin HR and Forman D. Cancer Incidence in Five Continents Time Trends, Volumes I to X and Volume XI: IARC CancerBase No. 9 [Internet]. Lyon, France: International Agency for Research on Cancer; 2010. Available from: http://ci5.iarc.fr

Flohr C, Weiland SK, Weinmayr G, Björkstén B, Bråbäck L, Brunekreef B, Büchele G, Clausen M, Cookson WOC, von Mutius E, Strachan DP, Williams HC, ISAAC Phase Two Study Group. The role of atopic sensitization in flexural eczema: findings from the International Study of Asthma and Allergies in Childhood Phase Two. J Allergy Clin Immunol. 2008; 121(1): 141-147

Flohr C, Weinmayr G, Weiland SK, Addo-Yobo E, Annesi-Maesano I, Björkstén B, Bråbäck L, Büchele G, Chico M, Cooper P, Clausen M, El Sharif N, Martinez Gimeno A, Mathur RS, von Mutius E, Morales Suarez-Varela M, Pearce N, Svabe V, Wong GWK, Yu M, Zhong NS, Williams HC, ISAAC Phase Two Study Group. How well do questionnaires perform compared with physical examination in detecting flexural eczema? Findings from the International Study of Asthma and Allergies in Childhood (ISAAC) Phase Two. Br J Dermatol. 2009; 161(4): 846-53

Food and Agriculture Organization of the United Nations (FAO). FAOSTAT Commodity Balances - Crops Primary Equivalent. Rome, Italy: Food and Agriculture Organization of the United Nations (FAO)

Food and Agriculture Organization of the United Nations (FAO). FAOSTAT Food Balance Sheets, October 2014. Rome, Italy: Food and Agriculture Organization of the United Nations (FAO)

Food Fortification Initiative (United States), Global Alliance for Improved Nutrition (GAIN), Iodine Global Network (Canada), Micronutrient Forum (Canada). Global Fortification Data Exchange: Legislation and Standards, Year When Food Fortification Mandated, and Count of Nutrients In Fortification Standards

Forman D, Bray F, Brewster DH, Gombe Mbalawa C, Kohler B, Piñeros M, Steliarova-Foucher E, Swaminathan R and Ferlay J, eds (2013). Cancer Incidence in Five Continents, Vol. X Summary Database (electronic version). Lyon, IARC. http://ci5.iarc.fr

Forsaa EB, Larsen JP, Wentzel-Larsen T, Goetz CG, Stebbins GT, Aarsland D, Alves G. A 12-year population-based study of psychosis in Parkinson disease. Arch Neurol. 2010; 67(8): 9961001

Furberg AS, Thune I. Metabolic abnormalities (hypertension, hyperglycemia and overweight), lifestyle (high energy intake and physical inactivity) and endometrial cancer risk in a Norwegian cohort. Int J Cancer. 2003; 104(6): 669-76

Gaard M, Tretli S, Løken EB. Dietary factors and risk of colon cancer: a prospective study of 50,535 young Norwegian men and women. Eur J Cancer Prev. 1996; 5(6): 445-54

Gallup. Gallup Analytics - World Poll Negative Experience Index. Washington, D.C., United States: Gallup, 2019

Gallup. Norway World Poll 2005-2006

Getz L, Luise Kirkengen A, Hetlevik I, Romundstad S, Sigurdsson JA. Ethical dilemmas arising from implementation of the European guidelines on cardiovascular disease prevention in clinical practice. Scand J Prim Health Care. 2004; 22(4): 202-8

Gjesdal CG, Aanderud SJ, Haga H-J, Brun JG, Tell GS. Femoral and whole-body bone mineral density in middle-aged and older Norwegian men and women: suitability of the reference values. Osteoporos Int. 2004; 15(7): 525-34

Global Burden of Disease Collaborative Network. Global Burden of Disease Study 2016 (GBD 2016) Covariates 1980-2016. Seattle, United States: Institute for Health Metrics and Evaluation (IHME), 2017

Global Burden of Disease Collaborative Network. Global Health Spending 1995-2017. Seattle, United States of America: Institute for Health Metrics and Evaluation (IHME), 2020

Graff-Iversen S, Jenum AK, Grøtvedt L, Bakken B, Selmer RM, Søgaard AJ. Risikofaktorer for hjerteinfarkt, hjerneslag og diabetes i Norge. Tidsskr Nor Laegeforen. 2007; 127(19): 2537-41

Grøholt E-K, Stigum H, Nordhagen R. Overweight and obesity among adolescents in Norway: cultural and socio-economic differences. J Public Health (Oxf). 2008; 30(3): 258-65

Grønlie SA, Myrvoll E, Hansen G, Grønning M, Mellgren SI. Multiple sclerosis in North Norway, and first appearance in an indigenous population. J Neurol. 2000; 247(2): 129-33

Gulati AM, Semb AG, Rollefstad S, Romundstad PR, Kavanaugh A, Gulati S, Haugeberg G, Hoff M. On the HUNT for cardiovascular risk factors and disease in patients with psoriatic arthritis: population-based data from the Nord-Trondelag Health Study. Ann Rheum Dis. 2016; 75(5): 819-24

Gulsvik AK, Thelle DS, Samuelsen SO, Myrstad M, Mowé M, Wyller TB. Ageing, physical activity and mortality—a 42-year follow-up study. Int J Epidemiol. 2012; 41(2): 521-30

Gustavson K, Knudsen AK, Nesvag R, Knudsen GP, Vollset SE, Reichborn-Kjennerud T. Prevalence, co-occurrence, and stability in common mental disorders in early to middle adulthood

Götestam KG, Agras WS. General Population-Based Epidemiological Study of Eating Disorders in Norway. Int J Eat Disord. 1995; 18(2): 119-26

Haarr D, Nessa J. [Treatment of opiate-dependent patients in a general practice]. Tidsskr Nor Laegeforen. 2007; 127(13): 1770-2

Hallan SI, Ovrehus MA, Romundstad S, Rifkin D, Langhammer A, Stevens PE, Ix JH. Long-term trends in the prevalence of chronic kidney disease and the influence of cardiovascular risk factors in Norway. Kidney Int. 2016; 90(3): 665-73

Halvorsen JA, Braae Olesen A, Thoresen M, Holm JØ, Bjertness E, Dalgard F. Comparison of self-reported skin complaints with objective skin signs among adolescents. Acta Derm Venereol. 2008; 88(6): 573-7

Hansen TE, Evjenth B, Holt J. Increasing prevalence of asthma, allergic rhinoconjunctivitis and eczema among schoolchildren: three surveys during the period 1985-2008. Acta Paediatr. 2013; 102(1): 47-52

Harstad I, Winje BA, Heldal E, Oftung F, Jacobsen Gw. Predictive values of QuantiFERON®-TB Gold testing in screening for tuberculosis disease in asylum seekers. Int J Tuberc Lung Dis. 2010; 14(9): 1209–11

Haugejorden O, Klock KS, Astrøm AN, Skaret E, Trovik TA. Socio-economic inequality in the self-reported number of natural teeth among Norwegian adults--an analytical study. Community Dent Oral Epidemiol. 2008; 36(3): 269-78

HBSC International Coordinating Centre. Foreign Born Children in Europe: an Overview from the Health Behavior in School-Aged Children (HSBC) Study . Geneva, Switzerland: International Organization for Migration (IOM), 2006

Heart Failure Data and Analysis 2000-2014 from the Cardiovascular Disease in Norway (CVDNOR) project (www.cvdnor.no). Correspondence with Dr. Gerhard Sulo, 2017. [Unpublished]

Heiervang E, Stormark KM, Lundervold AJ, Heimann M, Goodman R, Posserud M-B, Ullebø AK, Plessen KJ, Bjelland I, Lie SA, Gillberg C. Psychiatric disorders in Norwegian 8- to 10-year-olds: an epidemiological survey of prevalence, risk factors, and service use. J Am Acad Child Adolesc Psychiatry. 2007; 46(4): 438-47

Helseth S, Haraldstad K, Christophersen K-A. A cross-sectional study of Health Related Quality of Life and body mass index in a Norwegian school sample (8-18 years): a comparison of child and parent perspectives. Health Qual Life Outcomes. 2015; 13: 47

Henriksen BM, Ambjørnsen E, Axéll T. Dental caries among the elderly in Norway. Acta Odontol Scand. 2004; 62(2): 75-81

Henriksen BM, Ambjørnsen E, Laake K, Axéll T. Prevalence of teeth and dentures among elderly in Norway receiving social care. Acta Odontol Scand. 2003; 61(3): 184-91

Henriksen BM, Axéll T, Laake K. Geographic differences in tooth loss and denture-wearing among the elderly in Norway. Community Dent Oral Epidemiol. 2003; 31(6): 403-11

Hestetun I, Svendsen MV, Oellingrath IM. Associations between overweight, peer problems, and mental health in 12-13-year-old Norwegian children. Eur Child Adolesc Psychiatry. 2015; 24(3): 319–26

Hildrum B, Mykletun A, Hole T, Midthjell K, Dahl AA. Age-specific prevalence of the metabolic syndrome defined by the International Diabetes Federation and the National Cholesterol Education Program: the Norwegian HUNT 2 study. BMC Public Health. 2007; 7: 220

Hjartåker A, Knudsen MD, Tretli S, Weiderpass E. Consumption of berries, fruits and vegetables and mortality among 10,000 Norwegian men followed for four decades. Eur J Nutr. 2015; 54(4): 599-608

Holm M, Omenaas E, Gíslason T, Svanes C, Jögi R, Norrman E, Janson C, Torén K. Remission of asthma: a prospective longitudinal study from northern Europe (RHINE study). Eur Respir J. 2007; 30(1): 62-65

Holme I, Tonstad S, Sogaard AJ, Larsen PG, Haheim LL. Leisure time physical activity in middle age predicts the metabolic syndrome in old age: results of a 28-year follow-up of men in the Oslo study. BMC Public Health. 2007; 7(154)

Holst D. Oral health equality during 30 years in Norway. Community Dent Oral Epidemiol. 2008; 36(4): 326-34

Holter JC, Muller F, Bjorang O, Samdal HH, Marthinsen JB, Jenum PA, Ueland T, Froland SS, Aukrust P, Husebye E, Heggelund L. Etiology of community-acquired pneumonia and diagnostic yields of microbiological methods: a 3-year prospective study in Norway. BMC Infect Dis. 2015; 15: 64

Häggström C, Rapp K, Stocks T, Manjer J, Bjørge T, Ulmer H, Engeland A, Almqvist M, Concin H, Selmer R, Ljungberg B, Tretli S, Nagel G, Hallmans G, Jonsson H, Stattin P. Metabolic Factors Associated with Risk of Renal Cell Carcinoma. PLoS One. 2013; 8(2): e57475

Håberg SE, Stigum H, Nystad W, Nafstad P. Effects of pre- and postnatal exposure to parental smoking on early childhood respiratory health. Am J Epidemiol. 2007; 166(6): 679-86

Håheim LL, Holme I, Hjermann I, Leren P. Risk factors of stroke incidence and mortality. A 12-year follow-up of the Oslo Study. Stroke. 1993; 24(10): 1484-9

Idsoe T, Dyregrov A, Idsoe EC. Bullying and PTSD symptoms. J Abnorm Child Psychol. 2012; 40(6): 901–11

Institute of Basic Medical Sciences, University of Oslo, Norwegian Directorate of Health, Norwegian Food Safety Authority. Norway NORKOST National Food Consumption Survey 2010-2011

International Fuel Quality Center (IFQC). Overview of Leaded Gasoline and Sulfur Levels in Gasoline and Diesel . 2002

International Labour Organization (ILO). International Labour Organization Database (ILOSTAT) - Employment by Sex and Economic Activity. International Labour Organization (ILO)

International Labour Organization (ILO). International Labour Organization Database (ILOSTAT) - Employment by Sex and Occupation. International Labour Organization (ILO)

International Labour Organization (ILO). International Labour Organization Database (ILOSTAT) - Employment to Population Ratio by Sex and Age. International Labour Organization (ILO)

International Labour Organization (ILO). International Labour Organization Database (ILOSTAT) - Fatal Injuries by Sex and Economic Activity. International Labour Organization (ILO)

International Monetary Fund (IMF). World Economic Outlook Database. Washington, D.C., United States of America: International Monetary Fund (IMF)

International Road Federation. World Road Statistics 1963-1999. Geneva, Switzerland: International Road Federation

International Road Federation. World Road Statistics 2009 Database and World Road Statistics 2007

International Road Federation. World Road Statistics 2015. Geneva, Switzerland: International Road Federation, 2015

International Road Federation. World Road Statistics 2018. Geneva, Switzerland: International Road Federation, 2018

International Road Federation. World Road Statistics 2019. Geneva, Switzerland: International Road Federation, 2019

International Social Survey Programme (ISSP). Norway International Social Survey Program: Environment I 1992-1994. Mannheim, Germany: GESIS - Leibniz Institute for the Social Sciences, 1995

International Social Survey Programme (ISSP). Norway International Social Survey Programme: Citizenship 2004. Mannheim, Germany: GESIS - Leibniz Institute for the Social Sciences, 2012

International Social Survey Programme (ISSP). Norway International Social Survey Programme: Environment II 2000-2002. Mannheim, Germany: GESIS - Leibniz Institute for the Social Sciences, 2003

International Social Survey Programme (ISSP). Norway International Social Survey Programme: National Identity I 1994-1996. Mannheim, Germany: GESIS - Leibniz Institute for the Social Sciences, 1998

International Social Survey Programme (ISSP). Norway International Social Survey Programme: Religion III 2008. Mannheim, Germany: GESIS - Leibniz Institute for the Social Sciences, 2012

International Social Survey Programme (ISSP). Norway International Social Survey Programme: Role of Government III 1996. Mannheim, Germany: GESIS - Leibniz Institute for the Social Sciences, 1999

International Social Survey Programme (ISSP). Norway International Social Survey Programme: Social Inequality II 1992. Mannheim, Germany: GESIS - Leibniz Institute for the Social Sciences, 1994

International Social Survey Programme (ISSP). Norway International Social Survey Programme: Social Inequality III 1999. Mannheim, Germany: GESIS - Leibniz Institute for the Social Sciences, 2002

International Social Survey Programme (ISSP). Norway International Social Survey Programme: Work Orientation III 2005. Mannheim, Germany: GESIS - Leibniz Institute for the Social Sciences, 2013

ISSP Research Group (1992): International Social Survey Programme: Role of Government II - ISSP 1990. GESIS Data Archive, Cologne. ZA1950 Data file Version 1.0.0, https://doi.org/10.4232/1.1950

ISSP Research Group (1997): International Social Survey Programme: Family and Changing Gender Roles II - ISSP 1994. GESIS Data Archive, Cologne. ZA2620 Data file Version 1.0.0, doi:10.4232/1.2620

ISSP Research Group (1999): International Social Survey Programme: Work Orientations II - ISSP 1997. GESIS Data Archive, Cologne. ZA3090 Data file Version 1.0.0, doi:10.4232/1.3090

ISSP Research Group (2000): International Social Survey Programme: Religion II - ISSP 1998. GESIS Data Archive, Cologne. ZA3190 Data file Version 1.0.0, https://doi.org/10.4232/1.3190

ISSP Research Group (2003): International Social Survey Programme: Social Relations and Support Systems / Social Networks II - ISSP 2001. GESIS Data Archive, Cologne. ZA3680 Data file Version 1.0.0, doi:10.4232/1.3680

ISSP Research Group (2008): International Social Survey Programme: Role of Government IV - ISSP 2006. GESIS Data Archive, Cologne. ZA4700 Data file Version 1.0.0, doi:10.4232/1.4700

ISSP Research Group (2009): International Social Survey Programme: Health and Health Care - ISSP 2011. GESIS Data Archive, Cologne. ZA5800 Data file version 3.0.0, doi:10.4232/1.12252

ISSP Research Group (2009): International Social Survey Programme: Leisure Time and Sports - ISSP 2007. GESIS Data Archive, Cologne. ZA4850 Data file version 2.0.0, doi:10.4231/1.10079

ISSP Research Group (2012): International Social Survey Programme: Environment III - ISSP 2010. GESIS Data Archive, Cologne. ZA5500 Data file Version 2.0.0, doi:10.4232/1.11418.

ISSP Research Group (2012): International Social Survey Programme: National Identity II - ISSP 2003. GESIS Data Archive, Cologne. ZA3910 Data file Version 2.1.0, doi:10.4232/1.11449

ISSP Research Group (2013): International Social Survey Programme: Family and Changing Gender Roles III - ISSP 2002. GESIS Data Archive, Cologne. ZA3880 Data file Version 1.1.0, doi:10.4232/1.11564

ISSP Research Group (2015): International Social Survey Programme: National Identity III - ISSP 2013. GESIS Data Archive, Cologne. ZA5950 Data file Version 2.0.0, doi:10.4232/1.12312

ISSP Research Group (2016): International Social Survey Programme: Citizenship II - ISSP 2014. GESIS Data Archive, Cologne. ZA6670 Data file Version 2.0.0, doi:10.4232/1.12590

ISSP Research Group (2016): International Social Survey Programme: Family and Changing Gender Roles IV - ISSP 2012. GESIS Data Archive, Cologne. ZA5900 Data file Version 4.0.0, doi:10.4232/1.12661

ISSP Research Group (2017): International Social Survey Programme: Social Inequality IV - ISSP 2009. GESIS Data Archive, Cologne. ZA5400 Data file Version 4.0.0, doi:10.4232/1.12777.

Iversen B, Jacobsen BK, Lochen M-L. Active and passive smoking and the risk of myocardial infarction in 24,968 men and women during 11 year of follow-up: the Tromso Study. Eur J Epidemiol. 2013; 28(8): 659–67

Iversen T, Tretli S. Trends for invasive squamous cell neoplasia of the skin in Norway. Br J Cancer. 1999; 81(3): 528-31

Jacobs DR Jr, Meyer HE, Solvoll K. Reduced mortality among whole grain bread eaters in men and women in the Norwegian County Study. Eur J Clin Nutr. 2001; 55(2): 137-43

Jensen AJ, Kleveland CR, Moghaddam A, Haaheim H, Hjelmevoll SO, Skogen V. Chlamydia trachomatis, Mycoplasma genitalium and Ureaplasma urealyticum among students in northern Norway. J Eur Acad Dermatol Venereol. 2013; 27(1): e91-96

Jensen CL, Strand T, Ramberg GB, Ruden L, Anestad K, Norwegian Radiation Protection Authorities. The Norwegian Radon Mapping and Remediation Program. In: Proceedings of the 11th Congress of the International Radiation Protection Association (IRPA-11); 2004 May 23-28; Madrid, Spain

Johannessen A, Omenaas E, Bakke P, Gulsvik A. Incidence of GOLD-defined chronic obstructive pulmonary disease in a general adult population. Int J Tuberc Lung Dis. 2005; 9(8): 926–32

Johannessen EL, Andersson HW, Bjørngaard JH, Pape K. Anxiety and depression symptoms and alcohol use among adolescents -a cross sectional study of Norwegian secondary school students. BMC Public Health. 2017; 17(1): 494

Johansen H, Dammann B, Oinæs Andersen L, Andresen I-L. Children with congenital limb deficiency in Norway: issues related to school life and health-related quality of life. A cross-sectional study. Disabil Rehabil. 2016; 38(18): 1803–10

Johansen H, Østlie K, Andersen LØ, Rand-Hendriksen S. Adults with congenital limb deficiency in Norway: demographic and clinical features, pain and the use of health care and welfare services. A cross-sectional study. Disabil Rehabil. 2015; 37(22): 2076–82

Joint United Nations Program on HIV/AIDS (UNAIDS), Norwegian Institute of Public Health. Norway National HIV Strategy Report 2012-2013

Joner G, Stene LC, S?vik O. Nationwide, prospective registration of type 1 diabetes in children aged

Joseph J, Svartberg J, Njolstad I, Schirmer H. Incidence of and risk factors for type-2 diabetes in a general population: the Tromso Study. Scand J Public Health. 2010; 38(7): 768-75

Júlíusson PB, Eide GE, Roelants M, Waaler PE, Hauspie R, Bjerknes R. Overweight and obesity in Norwegian children: prevalence and socio-demographic risk factors. Acta Paediatr. 2010; 99(6): 900-5

Kamycheva E, Joakimsen RM, Jorde R. Intakes of calcium and vitamin d predict body mass index in the population of Northern Norway. J Nutr. 2003; 133(1): 102-6

Karvonen M, Viik-Kajander M, Moltchanova E, Libman I, LaPorte R, Tuomilehto J. Incidence of childhood type 1 diabetes worldwide. Diabetes Mondiale (DiaMond) Project Group. Diabetes Care. 2000; 23(10): 1516–26

Kildebo S, Nordgaard K, Aronsen O, Breckan R, Burhol PG, Jorde R. The incidence of ulcerative colitis in Northern Norway from 1983 to 1986. The Northern Norwegian Gastroenterology Society. Scand J Gastroenterol. 1990; 25(9): 890-6

Kinge JM, Knudsen AK, Skirbekk V, Vollset SE. Musculoskeletal disorders in Norway: prevalence of chronicity and use of primary and specialist health care services. BMC Musculoskelet Disord. 2015; 16(1): 75

Kjaer SK, Tran TN, Sparen P, Tryggvadottir L, Munk C, Dasbach E, Liaw K-L, Nygård J, Nygård M. The burden of genital warts: a study of nearly 70,000 women from the general female population in the 4 Nordic countries. J Infect Dis. 2007; 196(10): 1447–54

Konstantinova SV, Vollset SE, Berstad P, Ueland PM, Drevon CA, Refsum H, Tell GS. Dietary predictors of plasma total homocysteine in the Hordaland Homocysteine Study. Br J Nutr. 2007; 98(1): 201–10

Koposova N, Eriksen HM, Widström E, Handegård BH, Pastbin M, Koposov R. Caries prevalence and determinants among 12-year-olds in North-West Russia and Northern Norway. Stomatologija. 2013; 15(1): 3-11

Kringlen E, Torgersen S, Cramer V. A Norwegian psychiatric epidemiological study. Am J Psychiatry. 2001; 158(7): 1091-8

Kringlen E, Torgersen S, Cramer V. Mental illness in a rural area: a Norwegian psychiatric epidemiological study. Soc Psychiatry Psychiatr Epidemiol. 2006; 41(9): 713-9

Kristiansen AL, Lillegaard ITL, Lande B, Andersen LF. Effect of changes in an FFQ: comparing data from two national dietary survey instruments among 2-year-olds. Br J Nutr. 2013; 109(2): 363–9

Kristiansen HA, Kværner KJ, Akre H, Overland B, Russell MB. Migraine and sleep apnea in the general population. J Headache Pain. 2011; 12(1): 55-61

Krokstad S, Langhammer A, Hveem K, Holmen TL, Midthjell K, Stene TR, Bratberg G, Heggland J, Holmen J. Cohort Profile: The HUNT Study, Norway. Int J Epidemiol. 2013; 42(4): 968–77

Kvaavik E, Tell GS, Klepp K-I. Predictors and tracking of body mass index from adolescence into adulthood: follow-up of 18 to 20 years in the Oslo Youth Study. Arch Pediatr Adolesc Med. 2003; 157(12): 1212-8

Kvaerner KJ, Nafstad P, Jaakkola JJ. Upper respiratory morbidity in preschool children: a cross-sectional study. Arch Otolaryngol Head Neck Surg. 2000; 126(10): 1201-6

Kyrø C, Skeie G, Dragsted LO, Christensen J, Overvad K, Hallmans G, Johansson I, Lund E, Slimani N, Johnsen NF, Halkjær J, Tjønneland A, Olsen A. Intake of whole grain in Scandinavia: intake, sources and compliance with new national recommendations. Scand J Public Health. 2012; 40(1): 76–84

Laake I, Pedersen JI, Selmer R, Kirkhus B, Lindman AS, Tverdal A, Veierød MB. A prospective study of intake of trans-fatty acids from ruminant fat, partially hydrogenated vegetable oils, and marine oils and mortality from CVD. Br J Nutr. 2012; 108(4): 743-54

Lande B, Andersen LF, Baerug A, Trygg KU, Lund-Larsen K, Veierød MB, Bjørneboe GEA. Infant feeding practices and associated factors in the first six months of life: the Norwegian infant nutrition survey. Acta Paediatr. 2003; 92(2): 152-61

Langørgen J, Igland J, Vollset SE, Averina M, Nordrehaug JE, Tell GS, Irgens LM, Nygård O. Short-term and long-term case fatality in 11 878 patients hospitalized with a first acute myocardial infarction, 1979-2001: the Western Norway cardiovascular registry. Eur J Cardiovasc Prev Rehabil. 2009; 16(5): 621-627

Larsen JP, Kvåle G, Nyland H. Epidemiology of the Guillain-Barré syndrome in the county of Hordaland, Western Norway. Acta Neurol Scand. 1985; 71(1): 43-7

Leren P. The Oslo diet-heart study. Eleven-year report. Circulation. 1970; 42(5): 935-42

Lien L. The association between mental health problems and inflammatory conditions across gender and immigrant status: a population-based cross-sectional study among 10th-grade students. Scand J Public Health. 2008; 36(4): 353-60

Lindemann K, Vatten LJ, Ellstrøm-Engh M, Eskild A. Body mass, diabetes and smoking, and endometrial cancer risk: a follow-up study. Br J Cancer. 2008; 98(9): 1582–5

Lonnee-Hoffmann RAM, Salvesen O, Morkved S, Schei B. Self-reported pelvic organ prolapse surgery, prevalence, and nonobstetric risk factors: findings from the Nord Trøndelag Health Study. Int Urogynecol J. 2015; 26(3): 407–14

Maddison Project Database, version 2018. Bolt, Jutta, Robert Inklaar, Herman de Jong and Jan Luiten van Zanden (2018), "Rebasing 'Maddison': new income comparisons and the shape of long-run economic development", Maddison Project Working paper 10

Malaria Atlas Project. Sickle Haemoglobin (Hbs) Allele Frequency Layer. Oxford, United Kingdom: Malaria Atlas Project, 2013

Manger MS, Strand E, Ebbing M, Seifert R, Refsum H, Nordrehaug JE, Nilsen DW, Drevon CA, Tell GS, Bleie O, Vollset SE, Pedersen ER, Nygård O. Dietary intake of n-3 long-chain polyunsaturated fatty acids and coronary events in Norwegian patients with coronary artery disease. Am J Clin Nutr. 2010; 92(1): 244–51

Mannsverk J, Wilsgaard T, Mathiesen EB, Løchen ML, Rasmussen K, Thelle DS, Njølstad I, Hopstock LA, Bønaa KH. Trends in Modifiable Risk Factors are Associated With Declining Incidence of Hospitalized and Non-Hospitalized Acute Coronary Heart Disease in a Population. Circulation. 2016; 133(1): 74-81

Max Planck Institute for Demographic Research, University of California, Berkeley. Norway Human Mortality Database Deaths Period Data

Max Planck Institute for Demographic Research, Vienna Institute of Demography. Human Fertility Collection - ASFR, original age scale. Rostock, Germany: Max Planck Institute for Demographic Research

Max Planck Institute for Demographic Research, Vienna Institute of Demography. Human Fertility Database - Age-specific fertility rate. Rostock, Germany: Max Planck Institute for Demographic Research

Medbø A, Melbye H. Lung function testing in the elderly – can we still use FEV1/FVC< 70% as a criterion of COPD. Respir Med. 2007; 101(6): 1097-1105

Meltzer HM, Brantsaeter AL, Borch-Iohnsen B, Ellingsen DG, Alexander J, Thomassen Y, Stigum H, Ydersbond TA. Low iron stores are related to higher blood concentrations of manganese, cobalt and cadmium in non-smoking, Norwegian women in the HUNT 2 study. Environ Res. 2010; 110(5): 497–504

Meltzer HM, Dahl H, Brantsaeter AL, Birgisdottir BE, Knutsen HK, Bernhoft A, Oftedal B, Lande US, Alexander J, Haugen M, Ydersbond TA. Consumption of lead-shot cervid meat and blood lead concentrations in a group of adult Norwegians. Environ Res. 2013; 127: 29-39

Ministry of Education and Research (Norway). Baltic Sea Regional Study on Adolescents' Sexuality 2003-2004. Oslo, Norway: Ministry of Education and Research (Norway), 2007

Moen MH, Schei B. Epidemiology of endometriosis in a Norwegian county. Acta Obstet Gynecol Scand. 1997; 76(6): 559-62

Molvaer OI, Vårdal L, Gundersen T, Halmrast T. Hearing acuity in a Norwegian standard population. Scand Audiol. 1983; 12(4): 229-36

Moster D, Lie RT, Markestad T. Joint association of Apgar scores and early neonatal symptoms with minor disabilities at school age. Arch Dis Child Fetal Neonatal Ed. 2002; 86(1): F16-21

Moum B, Vatn MH, Ekbom A, Aadland E, Fausa O, Lygren I, Sauar J, Schulz T, Stray N. Incidence of ulcerative colitis and indeterminate colitis in four counties of southeastern Norway, 1990-93. A prospective population-based study. The Inflammatory Bowel South-Eastern Norway (IBSEN) Study Group of Gastroenterologists. Scand J Gastroenterol. 1996; 31(4): 362-6

Moum B, Vatn MH, Ekbom A, Aadland E, Fausa O, Lygren I, Stray N, Sauar J, Schulz T. Incidence of Crohn's disease in four counties in southeastern Norway, 1990-93. A prospective population-based study. The Inflammatory Bowel South-Eastern Norway (IBSEN) Study Group of Gastroenterologists. Scand J Gastroenterol. 1996; 31(4): 355-61

Moum B, Vatn MH, Ekbom A, Fausa O, Aadland E, Lygren I, Sauar J, Schulz T. Incidence of inflammatory bowel disease in southeastern Norway: evaluation of methods after 1 year of registration. Southeastern Norway IBD Study Group of Gastroenterologists. Digestion. 1995; 56(5): 377-81

Munkhaugen J, Lydersen S, Wideroe T-E, Hallan S. Prehypertension, obesity, and risk of kidney disease: 20-year follow-up of the HUNT I study in Norway. Am J Kidney Dis. 2009; 54(4): 638–46

Myklestad K, Vatten LJ, Salvesen KÅ, Davey Smith G, Romundstad PR. Hypertensive disorders in pregnancy and paternal cardiovascular risk: a population-based study. Ann Epidemiol. 2011; 21(6): 407-12

Naess Ø, Nafstad P, Aamodt G, Claussen B, Rosland P. Relation between concentration of air pollution and cause-specific mortality: four-year exposures to nitrogen dioxide and particulate matter pollutants in 470 neighborhoods in Oslo, Norway. Am J Epidemiol. 2007; 165(4): 435-43

Nafstad P, Jaakkola JJ, Hagen JA, Botten G, Kongerud J. Breastfeeding, maternal smoking and lower respiratory tract infections. Eur Respir J. 1996; 9(12): 2623–9

Nagel G, Weinmayr G, Flohr C, Kleiner A, Strachan DP. Association of pertussis and measles infections and immunizations with asthma and allergic sensitization in ISAAC Phase Two. Pediatr Allergy Immunol. 2012; 23(8): 737-46

Natvig GK, Albrektsen G, Qvarnstrøm U. School-Related Stress Experience as a Risk Factor for Bullying Behavior. J Youth Adolescence. 2001; 30(5): 561–75

Neroien AI, Schei B. Partner violence and health: results from the first national study on violence against women in Norway. Scand J Public Health. 2008; 36(2): 161-8

Nesv\a ag R, Knudsen GP, Bakken IJ, Høye A, Ystrom E, Surén P al, Reneflot A, Stoltenberg C, Reichborn-Kjennerud T. Substance use disorders in schizophrenia, bipolar disorder, and depressive illness: a registry-based study. Soc Psychiatry Psychiatr Epidemiol. 2015; 50(8): 1267

Nilsen DW, Albrektsen G, Landmark K, Moen S, Aarsland T, Woie L. Effects of a high-dose concentrate of n-3 fatty acids or corn oil introduced early after an acute myocardial infarction on serum triacylglycerol and HDL cholesterol. Am J Clin Nutr. 2001; 74(1): 50-6

Nilsen RM, Surén P, Gunnes N, Alsaker ER, Bresnahan M, Hirtz D, Hornig M, Lie KK, Lipkin WI, Reichborn-Kjennerud T, Roth C, Schjølberg S, Smith GD, Susser E, Vollset SE, Øyen A-S, Magnus P, Stoltenberg C. Analysis of

self-selection bias in a population-based cohort study of autism spectrum disorders. Paediatr Perinat Epidemiol. 2013; 27(6): 553–63

Nilsen RM, Vik ES, Rasmussen SA, Small R, Moster D, Schytt E, Aasheim V. Preeclampsia by maternal reasons for immigration: a population-based study. BMC Pregnancy Childbirth. 2018; 18(1): 423

Nilsen TI, Vatten LJ. A prospective study of lifestyle factors and the risk of pancreatic cancer in Nord-Trondelag, Norway. Cancer Causes Control. 2000; 11(7): 645-52

Nilsen TIL, Romundstad PR, Petersen H, Gunnell D, Vatten LJ. Recreational physical activity and cancer risk in subsites of the colon (the Nord-Trøndelag Health Study). Cancer Epidemiol Biomark Prev. 2008; 17(1): 183-8

Norderyd O, Henriksen BM, Jansson H. Periodontal disease in Norwegian old-age pensioners. Gerodontology. 2012; 29(1): 4-8

Nordic Medico-Statistical Committee (NOMESKO) (Denmark). Health Statistics for the Nordic Countries 2016. Copenhagen, Denmark: Nordic Medico-Statistical Committee (NOMESKO) (Denmark), 2016

Nordic Medico-Statistical Committee (NOMESKO) (Denmark). Health Statistics for the Nordic Countries 2017. Copenhagen, Denmark: Nordic Medico-Statistical Committee (NOMESKO) (Denmark), 2017

Nordic Medico-Statistical Committee (NOMESKO) (Denmark). Health Statistics in the Nordic Countries 1995. Copenhagen, Denmark: Nordic Medico-Statistical Committee (NOMESKO) (Denmark), 1997

Nordic Medico-Statistical Committee (NOMESKO) (Denmark). Health Statistics in the Nordic Countries 1996. Copenhagen, Denmark: Nordic Medico-Statistical Committee (NOMESKO) (Denmark), 1998

Nordic Medico-Statistical Committee (NOMESKO) (Denmark). Health Statistics in the Nordic Countries 1997. Copenhagen, Denmark: Nordic Medico-Statistical Committee (NOMESKO) (Denmark), 1999

Nordic Medico-Statistical Committee (NOMESKO) (Denmark). Health Statistics in the Nordic Countries 1998. Copenhagen, Denmark: Nordic Medico-Statistical Committee (NOMESKO) (Denmark), 2000

Nordic Medico-Statistical Committee (NOMESKO) (Denmark). Health Statistics in the Nordic Countries 1999. Copenhagen, Denmark: Nordic Medico-Statistical Committee (NOMESKO) (Denmark), 2001

Nordic Medico-Statistical Committee (NOMESKO) (Denmark). Health Statistics in the Nordic Countries 2000. Copenhagen, Denmark: Nordic Medico-Statistical Committee (NOMESKO) (Denmark), 2002

Nordic Medico-Statistical Committee (NOMESKO) (Denmark). Health Statistics in the Nordic Countries 2001. Copenhagen, Denmark: Nordic Medico-Statistical Committee (NOMESKO) (Denmark), 2003

Nordic Medico-Statistical Committee (NOMESKO) (Denmark). Health Statistics in the Nordic Countries 2002. Copenhagen, Denmark: Nordic Medico-Statistical Committee (NOMESKO) (Denmark), 2004

Nordic Medico-Statistical Committee (NOMESKO) (Denmark). Health Statistics in the Nordic Countries 2003. Copenhagen, Denmark: Nordic Medico-Statistical Committee (NOMESKO) (Denmark), 2005

Nordic Medico-Statistical Committee (NOMESKO) (Denmark). Health Statistics in the Nordic Countries 2004. Copenhagen, Denmark: Nordic Medico-Statistical Committee (NOMESKO) (Denmark), 2006

Nordic Medico-Statistical Committee (NOMESKO) (Denmark). Health Statistics in the Nordic Countries 2005. Copenhagen, Denmark: Nordic Medico-Statistical Committee (NOMESKO) (Denmark), 2007

Nordic Medico-Statistical Committee (NOMESKO) (Denmark). Health Statistics in the Nordic Countries 2006. Copenhagen, Denmark: Nordic Medico-Statistical Committee (NOMESKO) (Denmark), 2008

Nordic Medico-Statistical Committee (NOMESKO) (Denmark). Health Statistics in the Nordic Countries 2007. Copenhagen, Denmark: Nordic Medico-Statistical Committee (NOMESKO) (Denmark), 2009

Nordic Medico-Statistical Committee (NOMESKO) (Denmark). Health Statistics in the Nordic Countries with Data from 2008. Copenhagen, Denmark: Nordic Medico-Statistical Committee (NOMESKO) (Denmark), 2010

Norway - Oslo Health Study as it appears in Beelen R, Stafoggia M, Raaschou-Nielsen O, Andersen ZJ, Xun WW, Katsouyanni K, Dimakopoulou K, Brunekreef B, Weinmayr G, Hoffmann B, Wolf K, Samoli E, Houthuijs D, Nieuwenhuijsen M, Oudin A, Forsberg B, Olsson D, Salomaa V, Lanki T, Yli-Tuomi T, Oftedal B, Aamodt G, Nafstad P, De Faire U, Pedersen NL, Östenson CG, Fratiglioni L, Penell J, Korek M, Pyko A, Eriksen KT, Tjønneland A, Becker T, Eeftens M, Bots M, Meliefste K, Wang M, Bueno-de-Mesquita B, Sugiri D, Krämer U, Heinrich J, de Hoogh K, Key

T, Peters A, Cyrys J, Concin H, Nagel G, Ineichen A, Schaffner E, Probst-Hensch N, Dratva J, Ducret-Stich R, Vilier A, Clavel-Chapelon F, Stempfelet M, Grioni S, Krogh V, Tsai MY, Marcon A, Ricceri F, Sacerdote C, Galassi C, Migliore E, Ranzi A, Cesaroni G, Badaloni C, Forastiere F, Tamayo I, Amiano P, Dorronsoro M, Katsoulis M, Trichopoulou A, Vineis P, Hoek G. Long-term exposure to air pollution and cardiovascular mortality: an analysis of 22 European cohorts. Epidemiology. 2014; 25(3): 368–378

Norway Cancer Registry 1970 - CI5 as it appears in Ferlay J, Parkin DM, Curado MP, Bray F, Edwards B, Shin HR and Forman D. Cancer Incidence in Five Continents Time Trends, Volumes I to X and Volume XI: IARC CancerBase No. 9 [Internet]. Lyon, France: International Agency for Research on Cancer; 2010. Available from: http://ci5.iarc.fr

Norway Cancer Registry 1971 - CI5 as it appears in Ferlay J, Parkin DM, Curado MP, Bray F, Edwards B, Shin HR and Forman D. Cancer Incidence in Five Continents Time Trends, Volumes I to X and Volume XI: IARC CancerBase No. 9 [Internet]. Lyon, France: International Agency for Research on Cancer; 2010. Available from: http://ci5.iarc.fr

Norway Cancer Registry 1972 - CI5 as it appears in Ferlay J, Parkin DM, Curado MP, Bray F, Edwards B, Shin HR and Forman D. Cancer Incidence in Five Continents Time Trends, Volumes I to X and Volume XI: IARC CancerBase No. 9 [Internet]. Lyon, France: International Agency for Research on Cancer; 2010. Available from: http://ci5.iarc.fr

Norway Cancer Registry 1973 - CI5 as it appears in Ferlay J, Parkin DM, Curado MP, Bray F, Edwards B, Shin HR and Forman D. Cancer Incidence in Five Continents Time Trends, Volumes I to X and Volume XI: IARC CancerBase No. 9 [Internet]. Lyon, France: International Agency for Research on Cancer; 2010. Available from: http://ci5.iarc.fr

Norway Cancer Registry 1974 - CI5 as it appears in Ferlay J, Parkin DM, Curado MP, Bray F, Edwards B, Shin HR and Forman D. Cancer Incidence in Five Continents Time Trends, Volumes I to X and Volume XI: IARC CancerBase No. 9 [Internet]. Lyon, France: International Agency for Research on Cancer; 2010. Available from: http://ci5.iarc.fr

Norway Cancer Registry 1975 - CI5 as it appears in Ferlay J, Parkin DM, Curado MP, Bray F, Edwards B, Shin HR and Forman D. Cancer Incidence in Five Continents Time Trends, Volumes I to X and Volume XI: IARC CancerBase No. 9 [Internet]. Lyon, France: International Agency for Research on Cancer; 2010. Available from: http://ci5.iarc.fr

Norway Cancer Registry 1976 - CI5 as it appears in Ferlay J, Parkin DM, Curado MP, Bray F, Edwards B, Shin HR and Forman D. Cancer Incidence in Five Continents Time Trends, Volumes I to X and Volume XI: IARC CancerBase No. 9 [Internet]. Lyon, France: International Agency for Research on Cancer; 2010. Available from: http://ci5.iarc.fr

Norway Cancer Registry 1977 - CI5 as it appears in Ferlay J, Parkin DM, Curado MP, Bray F, Edwards B, Shin HR and Forman D. Cancer Incidence in Five Continents Time Trends, Volumes I to X and Volume XI: IARC CancerBase No. 9 [Internet]. Lyon, France: International Agency for Research on Cancer; 2010. Available from: http://ci5.iarc.fr

Norway Cancer Registry 1978 - CI5 as it appears in Ferlay J, Parkin DM, Curado MP, Bray F, Edwards B, Shin HR and Forman D. Cancer Incidence in Five Continents Time Trends, Volumes I to X and Volume XI: IARC CancerBase No. 9 [Internet]. Lyon, France: International Agency for Research on Cancer; 2010. Available from: http://ci5.iarc.fr

Norway Cancer Registry 1979 - CI5 as it appears in Ferlay J, Parkin DM, Curado MP, Bray F, Edwards B, Shin HR and Forman D. Cancer Incidence in Five Continents Time Trends, Volumes I to X and Volume XI: IARC CancerBase No. 9 [Internet]. Lyon, France: International Agency for Research on Cancer; 2010. Available from: http://ci5.iarc.fr

Norway Cancer Registry 1983-1987 - CI5 as it appears in Parkin DM, International Agency for Research on Cancer, International Association of Cancer Registries. Cancer Incidence in Five Continents. Vol. I to VIII. Lyon, France, IARCPress, 2005

Norway Cancer Registry 1988-1992 - CI5 as it appears in Parkin DM, International Agency for Research on Cancer, International Association of Cancer Registries. Cancer Incidence in Five Continents. Vol. I to VIII. Lyon, France, IARCPress, 2005

Norway Cancer Registry 2003-2007 - CI5 as it appears in Forman D, Bray F, Brewster DH, Gombe Mbalawa C, Kohler B, Piñeros M, Steliarova-Foucher E, Swaminathan R and Ferlay J, eds (2013). Cancer Incidence in Five Continents, Vol. X Summary Database (electronic version). Lyon, IARC. http://ci5.iarc.fr

Norway Cancer Registry 2008-2012 - CI5 as it appears in Bray F, Colombet M, Mery L, Piñeros M, Znaor A, Zanetti R and Ferlay J, editors (2017). Cancer Incidence in Five Continents, Vol. XI (electronic version). Lyon: International Agency for Research on Cancer. Available from: http://ci5.iarc.fr

Norway EUROCAT Prevalence Tables as it appears in European Surveillance of Congenital Anomalies (EUROCAT), University of Ulster. EUROCAT Prevalence Tables. Ispra, Italy: European Surveillance of Congenital Anomalies (EUROCAT)

Norway Medical Birth Registry 2006 - ICBDSR as it appears in International Clearinghouse for Birth Defects Surveillance and Research. International Clearinghouse for Birth Defects Surveillance and Research Annual Report 2008. Rome, Italy: International Clearinghouse for Birth Defects Surveillance and Research

Norway Medical Birth Registry 2011 - ICBDSR as it appears in International Clearinghouse for Birth Defects Surveillance and Research. International Clearinghouse for Birth Defects Surveillance and Research Annual Report 2013. Rome, Italy: International Clearinghouse for Birth Defects Surveillance and Research, 2015

Norway Medical Birth Registry Congenital Anomaly Data 1974 - ICBDMS as it appears in International Clearinghouse for Birth Defects Monitoring Systems. Congenital Malformations Worldwide: A Report from the International Clearinghouse for Birth Defects Monitoring Systems. Amsterdam, Netherlands: Elsevier, 1991

Norway Medical Birth Registry Congenital Anomaly Data 1975 - ICBDMS as it appears in International Clearinghouse for Birth Defects Monitoring Systems. Congenital Malformations Worldwide: A Report from the International Clearinghouse for Birth Defects Monitoring Systems. Amsterdam, Netherlands: Elsevier, 1991

Norway Medical Birth Registry Congenital Anomaly Data 1976 - ICBDMS as it appears in International Clearinghouse for Birth Defects Monitoring Systems. Congenital Malformations Worldwide: A Report from the International Clearinghouse for Birth Defects Monitoring Systems. Amsterdam, Netherlands: Elsevier, 1991

Norway Medical Birth Registry Congenital Anomaly Data 1977 - ICBDMS as it appears in International Clearinghouse for Birth Defects Monitoring Systems. Congenital Malformations Worldwide: A Report from the International Clearinghouse for Birth Defects Monitoring Systems. Amsterdam, Netherlands: Elsevier, 1991

Norway Medical Birth Registry Congenital Anomaly Data 1978 - ICBDMS as it appears in International Clearinghouse for Birth Defects Monitoring Systems. Congenital Malformations Worldwide: A Report from the International Clearinghouse for Birth Defects Monitoring Systems. Amsterdam, Netherlands: Elsevier, 1991

Norway Medical Birth Registry Congenital Anomaly Data 1979 - ICBDMS as it appears in International Clearinghouse for Birth Defects Monitoring Systems. Congenital Malformations Worldwide: A Report from the International Clearinghouse for Birth Defects Monitoring Systems. Amsterdam, Netherlands: Elsevier, 1991

Norway Medical Birth Registry Congenital Anomaly Data 1980 - ICBDMS as it appears in International Clearinghouse for Birth Defects Monitoring Systems. Congenital Malformations Worldwide: A Report from the International Clearinghouse for Birth Defects Monitoring Systems. Amsterdam, Netherlands: Elsevier, 1991

Norway Medical Birth Registry Congenital Anomaly Data 1981 - ICBDMS as it appears in International Clearinghouse for Birth Defects Monitoring Systems. Congenital Malformations Worldwide: A Report from the International Clearinghouse for Birth Defects Monitoring Systems. Amsterdam, Netherlands: Elsevier, 1991

Norway Medical Birth Registry Congenital Anomaly Data 1982 - ICBDMS as it appears in International Clearinghouse for Birth Defects Monitoring Systems. Congenital Malformations Worldwide: A Report from the International Clearinghouse for Birth Defects Monitoring Systems. Amsterdam, Netherlands: Elsevier, 1991

Norway Medical Birth Registry Congenital Anomaly Data 1983 - ICBDMS as it appears in International Clearinghouse for Birth Defects Monitoring Systems. Congenital Malformations Worldwide: A Report from the International Clearinghouse for Birth Defects Monitoring Systems. Amsterdam, Netherlands: Elsevier, 1991

Norway Medical Birth Registry Congenital Anomaly Data 1984 - ICBDMS as it appears in International Clearinghouse for Birth Defects Monitoring Systems. Congenital Malformations Worldwide: A Report from the International Clearinghouse for Birth Defects Monitoring Systems. Amsterdam, Netherlands: Elsevier, 1991

Norway Medical Birth Registry Congenital Anomaly Data 1985 - ICBDMS as it appears in International Clearinghouse for Birth Defects Monitoring Systems. Congenital Malformations Worldwide: A Report from the International Clearinghouse for Birth Defects Monitoring Systems. Amsterdam, Netherlands: Elsevier, 1991

Norway Medical Birth Registry Congenital Anomaly Data 1986 - ICBDMS as it appears in International Clearinghouse for Birth Defects Monitoring Systems. Congenital Malformations Worldwide: A Report from the International Clearinghouse for Birth Defects Monitoring Systems. Amsterdam, Netherlands: Elsevier, 1991

Norway Medical Birth Registry Congenital Anomaly Data 1987 - ICBDMS as it appears in International Clearinghouse for Birth Defects Monitoring Systems. Congenital Malformations Worldwide: A Report from the International Clearinghouse for Birth Defects Monitoring Systems. Amsterdam, Netherlands: Elsevier, 1991

Norway Medical Birth Registry Congenital Anomaly Data 1988 - ICBDMS as it appears in International Clearinghouse for Birth Defects Monitoring Systems. Congenital Malformations Worldwide: A Report from the International Clearinghouse for Birth Defects Monitoring Systems. Amsterdam, Netherlands: Elsevier, 1991

Norway Medical Birth Registry Congenital Anomaly Data 1993-1998 - WHO as it appears in European Surveillance of Congenital Anomalies (EUROCAT), International Centre on Birth Defects, World Health Organization (WHO). World Atlas of Birth Defects. 2nd ed. Geneva, Switzerland: World Health Organization (WHO), 2003

Norway Medical Birth Registry Congenital Anomaly Data 2001 - ICBDMS as it appears in International Clearinghouse for Birth Defects Monitoring Systems. International Clearinghouse for Birth Defects Monitoring Systems Annual Report 2003. Rome, Italy: International Centre on Birth Defects

Norway Medical Birth Registry Congenital Anomaly Data 2002 - ICBDMS as it appears in International Clearinghouse for Birth Defects Monitoring Systems. International Clearinghouse for Birth Defects Monitoring Systems Annual Report 2004. Rome, Italy: International Centre on Birth Defects, 2006

Norway Medical Birth Registry Congenital Anomaly Data 2003 - ICBDSR as it appears in International Clearinghouse for Birth Defects Surveillance and Research. International Clearinghouse for Birth Defects Surveillance and Research Annual Report 2005. Rome, Italy: International Clearinghouse for Birth Defects Surveillance and Research, 2007

Norway Medical Birth Registry Congenital Anomaly Data 2004 - ICBDSR as it appears in International Clearinghouse for Birth Defects Surveillance and Research. International Clearinghouse for Birth Defects Surveillance and Research Annual Report 2006. Rome, Italy: International Clearinghouse for Birth Defects Surveillance and Research, 2007

Norway Medical Birth Registry Congenital Anomaly Data 2005 - ICBDSR as it appears in International Clearinghouse for Birth Defects Surveillance and Research. International Clearinghouse for Birth Defects Surveillance and Research Annual Report 2007. Rome, Italy: International Clearinghouse for Birth Defects Surveillance and Research, 2008

Norway Medical Birth Registry Congenital Anomaly Data 2007 - ICBDSR as it appears in International Clearinghouse for Birth Defects Surveillance and Research. International Clearinghouse for Birth Defects Surveillance and Research Annual Report 2009. Rome, Italy: International Clearinghouse for Birth Defects Surveillance and Research

Norway Medical Birth Registry Congenital Anomaly Data 2008 - ICBDSR as it appears in International Clearinghouse for Birth Defects Surveillance and Research. International Clearinghouse for Birth Defects Surveillance and Research Annual Report 2010. Rome, Italy: International Clearinghouse for Birth Defects Surveillance and Research, 2011

Norway Medical Birth Registry Congenital Anomaly Data 2009 - ICBDSR as it appears in International Clearinghouse for Birth Defects Surveillance and Research. International Clearinghouse for Birth Defects Surveillance and Research Annual Report 2011. Rome, Italy: International Clearinghouse for Birth Defects Surveillance and Research, 2012

Norway Medical Birth Registry Congenital Anomaly Data 2010 - ICBDSR as it appears in International Clearinghouse for Birth Defects Surveillance and Research. International Clearinghouse for Birth Defects Surveillance and Research Annual Report 2012. Rome, Italy: International Clearinghouse for Birth Defects Surveillance and Research, 2013

Norway Population and Housing Census 2011 as it appears in United Nations Statistics Division (UNSD). United Nations Demographic Yearbook. New York City, United States: United Nations Statistics Division (UNSD)

Norway World Poll 2008 as it appears in Gallup. Gallup Analytics - World Poll Negative Experience Index. Washington, D.C., United States: Gallup, 2019

Norway World Poll 2012 as it appears in Gallup. Gallup Analytics - World Poll Negative Experience Index. Washington, D.C., United States: Gallup, 2019

Norway World Poll 2014 as it appears in Gallup. Gallup Analytics - World Poll Negative Experience Index. Washington, D.C., United States: Gallup, 2019

Norway World Poll 2015 as it appears in Gallup. Gallup Analytics - World Poll Negative Experience Index. Washington, D.C., United States: Gallup, 2019

Norway World Poll 2016 as it appears in Gallup. Gallup Analytics - World Poll Negative Experience Index. Washington, D.C., United States: Gallup, 2019

Norway World Poll 2017 as it appears in Gallup. Gallup Analytics - World Poll Negative Experience Index. Washington, D.C., United States: Gallup, 2019

Norwegian Centre for Violence and Traumatic Stress Studies (NKVTS). Norway Safety, Violence and Quality of Life Survey 2013

Norwegian Directorate of Health. Norway Patient Register 1990

Norwegian Directorate of Health. Norway Patient Register 1991

Norwegian Directorate of Health. Norway Patient Register 1992

Norwegian Directorate of Health. Norway Patient Register 1993

Norwegian Directorate of Health. Norway Patient Register 1994

Norwegian Directorate of Health. Norway Patient Register 1995

Norwegian Directorate of Health. Norway Patient Register 1996

Norwegian Directorate of Health. Norway Patient Register 1997

Norwegian Directorate of Health. Norway Patient Register 1998

Norwegian Directorate of Health. Norway Patient Register 1999

Norwegian Directorate of Health. Norway Patient Register 2000

Norwegian Directorate of Health. Norway Patient Register 2001

Norwegian Directorate of Health. Norway Patient Register 2002

Norwegian Directorate of Health. Norway Patient Register 2003

Norwegian Directorate of Health. Norway Patient Register 2008-2012

Norwegian Directorate of Health. Norway Patient Register 2009

Norwegian Directorate of Health. Norway Patient Register 2010

Norwegian Directorate of Health. Norway Patient Register 2011

Norwegian Directorate of Health. Norway Patient Register 2012

Norwegian Directorate of Health. Norway Patient Register 2013

Norwegian Directorate of Health. Norway Patient Register 2014

Norwegian Institute of Public Health, Tromsø Municipal Council (Norway), University Hospital of North Norway, University of Tromsø - The Arctic University of Norway. Norway Tromso Study 1994-2016

Norwegian Institute of Public Health. Norway Medical Birth Registry 1990. Oslo, Norway: Norwegian Institute of Public Health

Norwegian Institute of Public Health. Norway Medical Birth Registry 1991. Oslo, Norway: Norwegian Institute of Public Health

Norwegian Institute of Public Health. Norway Medical Birth Registry 1992. Oslo, Norway: Norwegian Institute of Public Health

Norwegian Institute of Public Health. Norway Medical Birth Registry 1993. Oslo, Norway: Norwegian Institute of Public Health

Norwegian Institute of Public Health. Norway Medical Birth Registry 1994. Oslo, Norway: Norwegian Institute of Public Health

Norwegian Institute of Public Health. Norway Medical Birth Registry 1995. Oslo, Norway: Norwegian Institute of Public Health

Norwegian Institute of Public Health. Norway Medical Birth Registry 1996. Oslo, Norway: Norwegian Institute of Public Health

Norwegian Institute of Public Health. Norway Medical Birth Registry 1997. Oslo, Norway: Norwegian Institute of Public Health

Norwegian Institute of Public Health. Norway Medical Birth Registry 1998. Oslo, Norway: Norwegian Institute of Public Health

Norwegian Institute of Public Health. Norway Medical Birth Registry 1999. Oslo, Norway: Norwegian Institute of Public Health

Norwegian Institute of Public Health. Norway Medical Birth Registry 2000. Oslo, Norway: Norwegian Institute of Public Health

Norwegian Institute of Public Health. Norway Medical Birth Registry 2001. Oslo, Norway: Norwegian Institute of Public Health

Norwegian Institute of Public Health. Norway Medical Birth Registry 2002. Oslo, Norway: Norwegian Institute of Public Health

Norwegian Institute of Public Health. Norway Medical Birth Registry 2003. Oslo, Norway: Norwegian Institute of Public Health

Norwegian Institute of Public Health. Norway Medical Birth Registry 2004. Oslo, Norway: Norwegian Institute of Public Health

Norwegian Institute of Public Health. Norway Medical Birth Registry 2005. Oslo, Norway: Norwegian Institute of Public Health

Norwegian Institute of Public Health. Norway Medical Birth Registry 2006. Oslo, Norway: Norwegian Institute of Public Health

Norwegian Institute of Public Health. Norway Medical Birth Registry 2007. Oslo, Norway: Norwegian Institute of Public Health

Norwegian Institute of Public Health. Norway Medical Birth Registry 2008. Oslo, Norway: Norwegian Institute of Public Health

Norwegian Institute of Public Health. Norway Medical Birth Registry 2009. Oslo, Norway: Norwegian Institute of Public Health

Norwegian Institute of Public Health. Norway Medical Birth Registry 2010. Oslo, Norway: Norwegian Institute of Public Health

Norwegian Institute of Public Health. Norway Medical Birth Registry 2011. Oslo, Norway: Norwegian Institute of Public Health

Norwegian Institute of Public Health. Norway Medical Birth Registry 2012. Oslo, Norway: Norwegian Institute of Public Health

Norwegian Institute of Public Health. Norway Medical Birth Registry 2013. Oslo, Norway: Norwegian Institute of Public Health

Norwegian Institute of Public Health. Norway Medical Birth Registry 2014. Oslo, Norway: Norwegian Institute of Public Health

Norwegian Institute of Public Health. Norway Norhealth Vaccination Coverage Statistics. Oslo, Norway: Norwegian Institute of Public Health

Norwegian Institute of Public Health. Norway Surveillance System for Communicable Diseases 1977. Oslo, Norway: Norwegian Institute of Public Health

Norwegian Institute of Public Health. Norway Surveillance System for Communicable Diseases 1978. Oslo, Norway: Norwegian Institute of Public Health

Norwegian Institute of Public Health. Norway Surveillance System for Communicable Diseases 1979. Oslo, Norway: Norwegian Institute of Public Health

Norwegian Institute of Public Health. Norway Surveillance System for Communicable Diseases 1980. Oslo, Norway: Norwegian Institute of Public Health

Norwegian Institute of Public Health. Norway Surveillance System for Communicable Diseases 1981. Oslo, Norway: Norwegian Institute of Public Health

Norwegian Institute of Public Health. Norway Surveillance System for Communicable Diseases 1982. Oslo, Norway: Norwegian Institute of Public Health

Norwegian Institute of Public Health. Norway Surveillance System for Communicable Diseases 1983. Oslo, Norway: Norwegian Institute of Public Health

Norwegian Institute of Public Health. Norway Surveillance System for Communicable Diseases 1984. Oslo, Norway: Norwegian Institute of Public Health

Norwegian Institute of Public Health. Norway Surveillance System for Communicable Diseases 1985. Oslo, Norway: Norwegian Institute of Public Health

Norwegian Institute of Public Health. Norway Surveillance System for Communicable Diseases 1986. Oslo, Norway: Norwegian Institute of Public Health

Norwegian Institute of Public Health. Norway Surveillance System for Communicable Diseases 1987. Oslo, Norway: Norwegian Institute of Public Health

Norwegian Institute of Public Health. Norway Surveillance System for Communicable Diseases 1988. Oslo, Norway: Norwegian Institute of Public Health

Norwegian Institute of Public Health. Norway Surveillance System for Communicable Diseases 1989. Oslo, Norway: Norwegian Institute of Public Health

Norwegian Institute of Public Health. Norway Surveillance System for Communicable Diseases 1990. Oslo, Norway: Norwegian Institute of Public Health

Norwegian Institute of Public Health. Norway Surveillance System for Communicable Diseases 1991. Oslo, Norway: Norwegian Institute of Public Health

Norwegian Institute of Public Health. Norway Surveillance System for Communicable Diseases 1992. Oslo, Norway: Norwegian Institute of Public Health

Norwegian Institute of Public Health. Norway Surveillance System for Communicable Diseases 1993. Oslo, Norway: Norwegian Institute of Public Health

Norwegian Institute of Public Health. Norway Surveillance System for Communicable Diseases 1994. Oslo, Norway: Norwegian Institute of Public Health

Norwegian Institute of Public Health. Norway Surveillance System for Communicable Diseases 1995. Oslo, Norway: Norwegian Institute of Public Health

Norwegian Institute of Public Health. Norway Surveillance System for Communicable Diseases 1996. Oslo, Norway: Norwegian Institute of Public Health

Norwegian Institute of Public Health. Norway Surveillance System for Communicable Diseases 1997. Oslo, Norway: Norwegian Institute of Public Health

Norwegian Institute of Public Health. Norway Surveillance System for Communicable Diseases 1998. Oslo, Norway: Norwegian Institute of Public Health

Norwegian Institute of Public Health. Norway Surveillance System for Communicable Diseases 1999. Oslo, Norway: Norwegian Institute of Public Health

Norwegian Institute of Public Health. Norway Surveillance System for Communicable Diseases 2000. Oslo, Norway: Norwegian Institute of Public Health

Norwegian Institute of Public Health. Norway Surveillance System for Communicable Diseases 2001. Oslo, Norway: Norwegian Institute of Public Health

Norwegian Institute of Public Health. Norway Surveillance System for Communicable Diseases 2002. Oslo, Norway: Norwegian Institute of Public Health

Norwegian Institute of Public Health. Norway Surveillance System for Communicable Diseases 2003. Oslo, Norway: Norwegian Institute of Public Health

Norwegian Institute of Public Health. Norway Surveillance System for Communicable Diseases 2004. Oslo, Norway: Norwegian Institute of Public Health

Norwegian Institute of Public Health. Norway Surveillance System for Communicable Diseases 2005. Oslo, Norway: Norwegian Institute of Public Health

Norwegian Institute of Public Health. Norway Surveillance System for Communicable Diseases 2006. Oslo, Norway: Norwegian Institute of Public Health

Norwegian Institute of Public Health. Norway Surveillance System for Communicable Diseases 2007. Oslo, Norway: Norwegian Institute of Public Health

Norwegian Institute of Public Health. Norway Surveillance System for Communicable Diseases 2008. Oslo, Norway: Norwegian Institute of Public Health

Norwegian Institute of Public Health. Norway Surveillance System for Communicable Diseases 2009. Oslo, Norway: Norwegian Institute of Public Health

Norwegian Institute of Public Health. Norway Surveillance System for Communicable Diseases 2010. Oslo, Norway: Norwegian Institute of Public Health

Norwegian Institute of Public Health. Norway Surveillance System for Communicable Diseases 2011. Oslo, Norway: Norwegian Institute of Public Health

Norwegian Institute of Public Health. Norway Surveillance System for Communicable Diseases 2012. Oslo, Norway: Norwegian Institute of Public Health

Norwegian Institute of Public Health. Norway Surveillance System for Communicable Diseases 2013. Oslo, Norway: Norwegian Institute of Public Health

Norwegian Institute of Public Health. Norway Surveillance System for Communicable Diseases 2014. Oslo, Norway: Norwegian Institute of Public Health

Norwegian Institute of Public Health. Norway Surveillance System for Communicable Diseases 2015. Oslo, Norway: Norwegian Institute of Public Health

Norwegian Institute of Public Health. Norway Surveillance System for Communicable Diseases 2016. Oslo, Norway: Norwegian Institute of Public Health

Norwegian Renal Registry Renal Replacement Therapy Data 1998 - ERA-EDTA as it appears in European Renal Association-European Dialysis and Transplant Association (ERA-EDTA). ERA-EDTA Registry Annual Report 1998. Amsterdam, Netherlands: Department of Medical Informatics, Academic Medical Center (The Netherlands), 2003

Norwegian Renal Registry Renal Replacement Therapy Data 1999 - ERA-EDTA as it appears in European Renal Association-European Dialysis and Transplant Association (ERA-EDTA). ERA-EDTA Registry Annual Report 1999. Amsterdam, Netherlands: Department of Medical Informatics, Academic Medical Center (The Netherlands), 2003

Norwegian Renal Registry Renal Replacement Therapy Data 2000 - ERA-EDTA as it appears in European Renal Association-European Dialysis and Transplant Association (ERA-EDTA). ERA-EDTA Registry Annual Report 2000. Amsterdam, Netherlands: Department of Medical Informatics, Academic Medical Center (The Netherlands), 2003

Norwegian Renal Registry Renal Replacement Therapy Data 2001 - ERA-EDTA as it appears in European Renal Association-European Dialysis and Transplant Association (ERA-EDTA). ERA-EDTA Registry Annual Report 2001. Amsterdam, Netherlands: Department of Medical Informatics, Academic Medical Center (The Netherlands), 2003

Norwegian Renal Registry Renal Replacement Therapy Data 2002 - ERA-EDTA as it appears in European Renal Association-European Dialysis and Transplant Association (ERA-EDTA). ERA-EDTA Registry Annual Report 2002. Amsterdam, Netherlands: Department of Medical Informatics, Academic Medical Center (The Netherlands), 2004

Norwegian Renal Registry Renal Replacement Therapy Data 2003 - ERA-EDTA as it appears in European Renal Association-European Dialysis and Transplant Association (ERA-EDTA). ERA-EDTA Registry Annual Report 2003. Amsterdam, Netherlands: Department of Medical Informatics, Academic Medical Center (The Netherlands), 2005

Norwegian Renal Registry Renal Replacement Therapy Data 2004 - ERA-EDTA as it appears in European Renal Association-European Dialysis and Transplant Association (ERA-EDTA). ERA-EDTA Registry Annual Report 2004. Amsterdam, Netherlands: Department of Medical Informatics, Academic Medical Center (The Netherlands), 2006

Norwegian Renal Registry Renal Replacement Therapy Data 2005 - ERA-EDTA as it appears in European Renal Association-European Dialysis and Transplant Association (ERA-EDTA). ERA-EDTA Registry Annual Report 2005. Amsterdam, Netherlands: Department of Medical Informatics, Academic Medical Center (The Netherlands), 2007

Norwegian Renal Registry Renal Replacement Therapy Data 2006 - ERA-EDTA as it appears in European Renal Association-European Dialysis and Transplant Association (ERA-EDTA). ERA-EDTA Registry Annual Report 2006. Amsterdam, Netherlands: Department of Medical Informatics, Academic Medical Center (The Netherlands), 2008

Norwegian Renal Registry Renal Replacement Therapy Data 2007 - ERA-EDTA as it appears in European Renal Association-European Dialysis and Transplant Association (ERA-EDTA). ERA-EDTA Registry Annual Report 2007. Amsterdam, Netherlands: Department of Medical Informatics, Academic Medical Center (The Netherlands), 2009

Norwegian Renal Registry Renal Replacement Therapy Data 2008 - ERA-EDTA as it appears in European Renal Association-European Dialysis and Transplant Association (ERA-EDTA). ERA-EDTA Registry Annual Report 2008. Amsterdam, Netherlands: Department of Medical Informatics, Academic Medical Center (The Netherlands), 2010

Norwegian Renal Registry Renal Replacement Therapy Data 2009 - ERA-EDTA as it appears in European Renal Association-European Dialysis and Transplant Association (ERA-EDTA). ERA-EDTA Registry Annual Report 2009. Amsterdam, Netherlands: Department of Medical Informatics, Academic Medical Center (The Netherlands), 2011

Norwegian Renal Registry Renal Replacement Therapy Data 2010 - ERA-EDTA as it appears in European Renal Association-European Dialysis and Transplant Association (ERA-EDTA). ERA-EDTA Registry Annual Report 2010. Amsterdam, Netherlands: Department of Medical Informatics, Academic Medical Center (The Netherlands), 2012

Norwegian Renal Registry Renal Replacement Therapy Data 2011 - ERA-EDTA as it appears in European Renal Association-European Dialysis and Transplant Association (ERA-EDTA). ERA-EDTA Registry Annual Report 2011. Amsterdam, Netherlands: Department of Medical Informatics, Academic Medical Center (The Netherlands), 2013

Norwegian Renal Registry Renal Replacement Therapy Data 2012 - ERA-EDTA as it appears in European Renal Association-European Dialysis and Transplant Association (ERA-EDTA). ERA-EDTA Registry Annual Report 2012. Amsterdam, Netherlands: Department of Medical Informatics, Academic Medical Center (The Netherlands), 2014

Norwegian Renal Registry Renal Replacement Therapy Data 2013 - ERA-EDTA as it appears in European Renal Association-European Dialysis and Transplant Association (ERA-EDTA). ERA-EDTA Registry Annual Report 2013. Amsterdam, Netherlands: Department of Medical Informatics, Academic Medical Center (The Netherlands), 2015

Norwegian Renal Registry Renal Replacement Therapy Data 2014 - ERA-EDTA as it appears in European Renal Association-European Dialysis and Transplant Association (ERA-EDTA). ERA-EDTA Registry Annual Report 2014. Amsterdam, Netherlands: Department of Medical Informatics, Academic Medical Center (The Netherlands), 2016

Nystad W, Meyer HE, Nafstad P, Tverdal A, Engeland A. Body mass index in relation to adult asthma among 135,000 Norwegian men and women. Am J Epidemiol. 2004; 160(10): 969–76

Oellingrath IM, Svendsen MV, Brantsaeter AL. Eating patterns and overweight in 9- to 10-year-old children in Telemark County, Norway: a cross-sectional study. Eur J Clin Nutr. 2010; 64(11): 1272-9

Olsen AO, Grjibovski A, Magnus P, Tambs K, Harris JR. Psoriasis in Norway as observed in a population-based Norwegian twin panel. Br J Dermatol. 2005; 153(2): 346-51

Olsen C, Pedersen I, Bergland A, Enders-Slegers MJ, Jøranson N, Calogiuri G, Ihlebæk C. Differences in quality of life in home-dwelling persons and nursing home residents with dementia - a cross-sectional study. BMC Geriatr. 2016; 16: 137

Omdal T, Dale J, Lie SA, Iversen KB, Flaatten H, Ovrebo K. Time trends in incidence, etiology, and case fatality rate of the first attack of acute pancreatitis. Scand J Gastroenterol. 2011; 46(11): 1389-98

Opdahl S, Alsaker MDK, Janszky I, Romundstad PR, Vatten LJ. Joint effects of nulliparity and other breast cancer risk factors. Br J Cancer. 2011; 105(5): 731-36

Organization for Economic Co-operation and Development (OECD). Norway Survey of Adult Skills Round I 2011-2012. Paris, France: Organization for Economic Co-operation and Development (OECD)

Organization for Economic Co-operation and Development (OECD). Organization for Economic Co-operation and Development Data - Hospital Beds. Paris, France: Organization for Economic Co-operation and Development (OECD)

P.N. Lee Statistics and Computing Ltd. International Mortality and Smoking Statistics Version 4.04. Sutton, United Kingdom: P.N. Lee Statistics and Computing Ltd, 2009

Park SL, Goodman MT, Zhang Z-F, Kolonel LN, Henderson BE, Setiawan VW. Body size, adult BMI gain and endometrial cancer risk: the multiethnic cohort. Int J Cancer. 2010; 126(2): 490-99

Parkin DM, International Agency for Research on Cancer, International Association of Cancer Registries. Cancer Incidence in Five Continents. Vol. I to VIII. Lyon, France, IARCPress, 2005

Parr CL, Hjartåker A, Lund E, Veierød MB. Meat intake, cooking methods and risk of proximal colon, distal colon and rectal cancer: the Norwegian Women and Cancer (NOWAC) cohort study. Int J Cancer. 2013; 133(5): 1153-63

Pedersen W, Skrondal A. Alcohol and sexual victimization: a longitudinal study of Norwegian girls. Addiction. 1996; 91(4): 565-81

Perminow G, Brackmann S, Lyckander LG, Franke A, Borthne A, Rydning A, Aamodt G, Schreiber S, Vatn MH, IBSEN-II Group. A characterization in childhood inflammatory bowel disease, a new population-based inception cohort from South-Eastern Norway, 2005-07, showing increased incidence in Crohn's disease. Scand J Gastroenterol. 2009; 44(4): 446–56

Pew Research Center. The Future of the Global Muslim Population. Washington, DC, United States: Pew Research Center, 2011

Popova S, Lange S, Probst C, Gmel G, Rehm J, Centre for Addiction and Mental Health (Canada). Estimation of national, regional and global prevalence of alcohol use during pregnancy and fetal alcohol syndrome: a systematic review and meta-analysis. Lancet Glob Health. [Forthcoming]

Queen Sofía Center for the Study of Violence. Partner Violence Against Women: Statistics and Legislation, 3rd International Report 2010. Valencia, Spain: Queen Sofía Center for the Study of Violence, 2010

Qureshi SA, Lund AC, Veierød MB, Carlsen MH, Blomhoff R, Andersen LF, Ursin G. Food items contributing most to variation in antioxidant intake; a cross-sectional study among Norwegian women. BMC Public Health. 2014; 14: 45

Rinaldi S, Lise M, Clavel-Chapelon F, Boutron-Ruault M-C, Guillas G, Overvad K, Tjønneland A, Halkjær J, Lukanova A, Kaaks R, Bergmann MM, Boeing H, Trichopoulou A, Zylis D, Valanou E, Palli D, Agnoli C, Tumino R, Polidoro S, Mattiello A, Bas Bueno-de-Mesquita H, Peeters PH, Weiderpass E, Lund E, Skeie G, Rodríguez L, Travier N, Sánchez M-J, Amiano P, Huerta J-M, Ardanaz E, Rasmuson T, Hallmans G, Almquist M, Manjer J, Tsilidis KK, Allen NE, Khaw K-T, Wareham N, Byrnes G, Romieu I, Riboli E, Franceschi S. Body size and risk of differentiated thyroid carcinomas: Findings from the EPIC study. Int J Cancer. 2012; 131(6): E1004–14

Ro AD, Simpson MR, Storro O, Johnsen R, Videm V, Oien T. The predictive value of allergen skin prick tests and IgE tests at pre-school age: the PACT study. Pediatr Allergy Immunol. 2014; 25(7): 691-8

Roberts CL, Ford JB, Algert CS, Antonsen S, Chalmers J, Cnattingius S, Gokhale M, Kotelchuck M, Melve KK, Langridge A, Morris C, Morris JM, Nassar N, Norman JE, Norrie J, Sørensen HT, Walker R, Weir CJ. Population-

based trends in pregnancy hypertension and pre-eclampsia: an international comparative study. BMJ Open. 2011; 1(1): e000101

Roland E. Bullying, depressive symptoms and suicidal thoughts. Educ Res. 2002; 44(1): 55–67

Rossow I, Hawton K, Ystgaard M. Cannabis use and deliberate self-harm in adolescence: a comparative analysis of associations in England and Norway. Arch Suicide Res. 2009; 13(4): 340–8

Rossow I. Suicide among drug addicts in Norway. Addiction. 1994; 89(12): 1667-73

Sandanger I, Nygård JF, Ingebrigtsen G, Sørensen T, Dalgard OS. Prevalence, incidence and age at onset of psychiatric disorders in Norway. Soc Psychiatry Psychiatr Epidemiol. 1999; 34(11): 570-9

Sanmartin J, Molina A, Garcia Y, Queen Sofía Center for the Study of Violence. Partner Violence against Women: Statistics and Legislation, International Report 2003. Valencia, Spain: Queen Sofía Center for the Study of Violence, 2003

Sanmartin J, Queen Sofía Center for the Study of Violence. Partner Violence against Women: Statistics and Legislation, 2nd International Report 2007. Valencia, Spain: Queen Sofía Center for the Study of Violence, 2007

Schei B, Muus KM, Bendixen M. [Occurrence of sexual abuse among students in Trondheim]. Tidsskr Nor Laegeforen. 1994; 114(21): 2491-4

Schei B. Prevalence of Sexual Abuse History in a Random Sample of Norwegian Women. Scand J Soc Med. 1990; 18(1): 63-8

Schlesinger S, Aleksandrova K, Pischon T, Fedirko V, Jenab M, Trepo E, Boffetta P, Dahm CC, Overvad K, Tjønneland A, Halkjær J, Fagherazzi G, Boutron-Ruault MC, Carbonnel F, Kaaks R, Lukanova A, Boeing H, Trichopoulou A, Bamia C, Lagiou P, Palli D, Grioni S, Panico S, Tumino R, Vineis P, Bueno-de-Mesquita HB, van den Berg S, Peeters PH, Braaten T, Weiderpass E, Quirós JR, Travier N, Sánchez MJ, Navarro C, Barricarte A, Dorronsoro M, Lindkvist B, Regner S, Werner M, Sund M, Khaw KT, Wareham N, Travis RC, Norat T, Wark PA, Riboli E, Nöthlings U. Abdominal obesity, weight gain during adulthood and risk of liver and biliary tract cancer in a European cohort. Int J Cancer. 2013; 132(3): 645-57

Schmitt, H., Scholz, E., Leim, I., Moschner, M. (2008): The Mannheim Eurobarometer Trend File 1970-2002 (ed. 2.00). European Commission [Principal investigator]. GESIS Data Archive, Cologne. ZA3521 Data file Version 2.0.1, doi:10.4232/1.10074

Schuller AA, Holst D. Changes in the oral health of adults from Trøndelag, Norway, 1973-1983-1994. Community Dent Oral Epidemiol. 1998; 26(3): 201-8

Seland JH, Vingerling JR, Augood CA, Bentham G, Chakravarthy U, deJong PTVM, Rahu M, Soubrane G, Tomazzoli L, Topouzis F, Fletcher AE. Visual impairment and quality of life in the older European population, the EUREYE study. Acta Ophthalmol. 2011; 89(7): 608-13

Selnes A, Nystad W, Bolle R, Lund E. Diverging prevalence trends of atopic disorders in Norwegian children. Results from three cross-sectional studies. Allergy. 2005; 60(7): 894–9

Shi Y, Lenzi M, An R. Cannabis Liberalization and Adolescent Cannabis Use: A Cross-National Study in 38 Countries. PLoS One. 2015; 10(11): e0143562

Siebke JC, Degré M, Ritland S, Enger SC. Prevalence of hepatitis A antibodies in a normal population and some selected groups of patients in Norway. Am J Epidemiol. 1982; 115(2): 185-91

Sigurdson JF, Undheim AM, Wallander JL, Lydersen S, Sund AM. The Longitudinal Association of Being Bullied and Gender with Suicide Ideations, Self-Harm, and Suicide Attempts from Adolescence to Young Adulthood: A Cohort Study. Suicide Life Threat Behav. 2017; 1-4

Skjeldestad FE, Jerve F. Chlamydia trachomatis and Neisseria gonorrhoeae among women seeking abortion in Norway. Results from a nationwide study. Tidsskr Nor Laegeforen. 1992; 112(16): 2082-4

Skjeldestad FE. Choice of contraceptive modality by women in Norway. Acta Obstet Gynecol Scand. 1994; 73(1): 48-52

Skjeldestad FE. Prevensjonsbruken i Norge i 2005. Tidsskr Nor Laegeforen. 2007; 21(127): 2803-2805

Skrivarhaug T, Stene LC, Drivvoll AK, Str?m H, Joner G. Incidence of type 1 diabetes in Norway among children aged 0-14 years between 1989 and 2012: has the incidence stopped rising? Results from the Norwegian Childhood Diabetes Registry. Diabetologia. 2014; 57(1): 57-62

Skudutyte-Rysstad R, Eriksen HM, Hansen BF. Trends in periodontal health among 35-year-olds in Oslo, 1973-2003. J Clin Periodontol. 2007; 34(10): 867-72

Smidesang I, Saunes M, Storrø O, Øien T, Holmen TL, Johnsen R, Henriksen AH. Atopic dermatitis among 2-year olds; high prevalence, but predominantly mild disease--the PACT study, Norway. Pediatr Dermatol. 2008; 25(1): 13-8

Solberg ME, Olweus D, Endresen IM. Bullies and Victims at School: Are They the Same Pupils?. Br J Educ Psychol. 2007; 77(2): 441–64

Solbraa AK, Holme IM, Graff-Iversen S, Resaland GK, Aadland E, Anderssen SA. Physical activity and cardiovascular risk factors in a 40- to 42-year-old rural Norwegian population from 1975-2010: repeated cross-sectional surveys. BMC Public Health. 2014; 14: 569

Sole KB, Staff AC, Laine K. The association of maternal country of birth and education with hypertensive disorders of pregnancy: A population-based study of 960 516 deliveries in Norway. Acta Obstet Gynecol Scand. 2018; 97(10): 1237-1247

Sorensen M, Wickman M, Sollid JU, Furberg AS, Klingenberg C. Allergic disease and Staphylococcus aureus carriage in adolescents in the Arctic region of Norway. Pediatr Allergy Immunol. 2016; nan

Spain Renal Replacement Therapy Data 2011 - ERA-EDTA as it appears in European Renal Association-European Dialysis and Transplant Association (ERA-EDTA). ERA-EDTA Registry Annual Report 2011. Amsterdam, Netherlands: Department of Medical Informatics, Academic Medical Center (The Netherlands), 2013

Statistics Norway. Norway Census 1980

Statistics Norway. Norway Census 1990

Statistics Norway. Norway Census 2001

Statistics Norway. Norway Deaths by Sex and Age. Oslo, Norway: Statistics Norway

Statistics Norway. Norway Lifestyle Habits by Gender and Age. Oslo, Norway: Statistics Norway

Statistics Norway. Norway Long-term Illness: Disease, Injury or Impairment by Sex and Age. Oslo, Norway: Statistics Norway

Statistics Norway. Norway Statbank - Births, by Sex. Oslo, Norway: Statistics Norway

Statistics Norway. Norway Statbank - Live Births, by Region, Age of Mother at Child's Birth. Oslo, Norway: Statistics Norway

Statistics Norway. Norway Statbank - Population by Sex and One Year Age Groups. Oslo, Norway: Statistics Norway

Statistics Norway. Norway Survey of Living Conditions 2002. Oslo, Norway: Statistics Norway

Statistics Norway. Norway Survey of Living Conditions 2005-2006. Oslo, Norway: Statistics Norway

Statistics Norway. Norway Survey of Living Conditions 2008-2009. Oslo, Norway: Statistics Norway

Statistics Norway. Norway Survey of Living Conditions 2012-2013

Statistics Norway. Norway Survey of Living Conditions on Health 2015

Steins Bisschop CN, van Gils CH, Emaus MJ, Bueno-de-Mesquita HB, Monninkhof EM, Boeing H, Aleksandrova K, Jenab M, Norat T, Riboli E, Boutron-Rualt MC, Fagherazzi G, Racine A, Palli D, Krogh V, Tumino R, Naccarati A, Mattiello A, Argüelles MV, Sanchez MJ, Tormo MJ, Ardanaz E, Dorronsoro M, Bonet C, Khaw KT, Key T, Trichopoulou A, Orfanos P, Naska A, Kaaks RR, Lukanova A, Pischon T, Ljuslinder I, Jirström K, Ohlsson B, Overvad K, Landsvig Berentzen T, Halkjaer J, Tjonneland A, Weiderpass E, Skeie G, Braaten T, Siersema PD, Freisling H, Ferrari P, Peeters PH, May AM. Weight change later in life and colon and rectal cancer risk in participants in the EPIC-PANACEA study. Am J Clin Nutr. 2014; 99(1): 139-47

Strøm H, Selmer R, Birkeland KI, Schirmer H, Berg TJ, Jenum AK, Midthjell K, Berg C, Stene LC. No increase in new users of blood glucose-lowering drugs in Norway 2006-2011: a nationwide prescription database study. BMC Public Health. 2014; 14(520)

Strømme P, Valvatne K. Mental retardation in Norway: prevalence and sub-classification in a cohort of 30037 children born between 1980 and 1985. Acta Paediatr. 1998; 87(3): 291-6

Sulo E, Vollset SE, Nygård O, Sulo G, Igland J, Egeland GM, Ebbing M, Tell GS. Trends in 28-day and 1-year mortality rates in patients hospitalized for a first acute myocardial infarction in Norway during 2001-2009: a "Cardiovascular disease in Norway" (CVDNOR) project. J Intern Med. 2015; 277(3): 353–61

Sund AM, Larsson B, Wichstrøm L. Prevalence and characteristics of depressive disorders in early adolescents in central Norway. Child Adolesc Psychiatry Ment Health. 2011; 5: 28

Surén P, Bakken IJ, Aase H, Chin R, Gunnes N, Lie KK, Magnus P, Reichborn-Kjennerud T, Schjølberg S, Øyen A-S, Stoltenberg C. Autism spectrum disorder, ADHD, epilepsy, and cerebral palsy in Norwegian children. Pediatrics. 2012; 130(1): e152-158

Suren P, Schjolberg S, Oyen A, Lie K, Hornig M, Bresnahan M, Bakke T, Roth C, Alsaker E, Schreuder P, Stenberg N, Reichborn-Kjennerud T, Hirtz D, Susser E, Magnus P, Lipkin W, Stoltenberg C. The Autism Birth Cohort (ABC):a study of autism spectrum disorders in MoBa. Nor Epidemiol. 2014; 24(1-2): 39-50

Sönju Clasen AB, von der Fehr FR, Kant van Daal JM. Caries prevalence of kindergarten children in Salzgitter and Oslo. Caries Res. 1992; 26(3): 201-4

Tambs K, Hoffman HJ, Borchgrevink HM, Holmen J, Samuelsen SO. Hearing loss induced by noise, ear infections, and head injuries: results from the Nord-Trøndelag Hearing Loss Study. Int J Audiol. 2003; 42(2): 89-105

The International Social Survey Programme (ISSP) represents a series of cross-national collaborations between organizations conducting similar social science research. ISSP researchers focus on developing survey questions that are relevant and comprehensible in all countries and languages, with survey results that are comparable across nations.Topics covered on this round of the ISSP include: working conditions; labor relations and conflict; and employment and unemployment

Thoresen S, Myhre M, Wentzel-Larsen T, Aakvaag HF, Hjemdal OK. Violence against children, later victimisation, and mental health: a cross-sectional study of the general Norwegian population. Eur J Psychotraumatol. 2015; 6(1): 26259

Thune I, Lund E. Physical activity and risk of colorectal cancer in men and women. Br J Cancer. 1996; 73(9): 1134-40

Tveiten A, Ljøstad U, Mygland A, Thomassen L, Pripp AH, Naess H. Intracerebral hemorrhage in southern Norway – a hospital-based incidence study. Eur Neurol. 2012; 67(4): 240-5

Tverdal A, Thelle D, Stensvold I, Leren P, Bjartveit K. Mortality in relation to smoking history: 13 years' follow-up of 68,000 Norwegian men and women 35–49 years. J Clin Epidemiol. 1993; 46(5): 475–87

U.S. Department of Agriculture (USDA). USDA Global Tobacco Database 1960-2005. Washington D.C. , United States: U.S. Department of Agriculture (USDA)

Undheim AM, Sund AM. Prevalence of bullying and aggressive behavior and their relationship to mental health problems among 12- to 15-year-old Norwegian adolescents. Eur Child Adolesc Psychiatry. 2010; 19(11): 803–11

United Nations Children's Fund (UNICEF), World Health Organization (WHO). WHO and UNICEF Reported Disease Incidence Time Series. Geneva, Switzerland: World Health Organization (WHO)

United Nations Children's Fund (UNICEF), World Health Organization (WHO). WHO and UNICEF Reported Official Target Population, Number of Doses Administered and Official Coverage 1966-2018. Geneva, Switzerland: World Health Organization (WHO), 2019

United Nations Children's Fund (UNICEF). UNICEF Maternal and Newborn Health Coverage Database as of November 2019. New York, United States of America: United Nations Children's Fund (UNICEF), 2019

United Nations Environment Programme. Leaded Petrol Phase-out: Global Status, March 2018. Nairobi, Kenya: United Nations Environment Programme

United Nations Office on Drugs and Crime (UNODC). World Drug Report 2012. Vienna, Austria: United Nations Office on Drugs and Crime (UNODC), 2012

United Nations Population Division. Abortion Policies: A Global Review. New York, United States of America: United Nations (UN), 2002

United Nations Population Division. World Abortion Policies 2007. New York, United States of America: United Nations (UN), 2007

United Nations Population Division. World Abortion Policies 2013. New York, United States of America: United Nations (UN), 2013

United Nations Statistics Division (UNSD). National Accounts Main Aggregates Database. New York City, United States of America: United Nations Statistics Division (UNSD)

United Nations Statistics Division (UNSD). United Nations Demographic Yearbook - Historical Supplement 1997. New York, United States of America: United Nations (UN)

United Nations Statistics Division (UNSD). United Nations Demographic Yearbook 2000. New York, United States: United Nations (UN), 2002

United Nations Statistics Division (UNSD). United Nations Demographic Yearbook 2001. New York, United States: United Nations (UN), 2003

United Nations Statistics Division (UNSD). United Nations Demographic Yearbook 2004. New York, United States: United Nations (UN), 2007

United Nations Statistics Division (UNSD). United Nations Demographic Yearbook 2006. New York, United States: United Nations (UN), 2008

United Nations Statistics Division (UNSD). United Nations Demographic Yearbook 2014. New York, United States: United Nations (UN), 2015

United Nations Statistics Division (UNSD). United Nations Demographic Yearbook 2015. New York, United States of America: United Nations (UN), 2016

United Nations Statistics Division (UNSD). United Nations Demographic Yearbook. New York City, United States: United Nations Statistics Division (UNSD)

Van der Sluis ME, Lien N, Twisk JWR, Steenhuis IHM, Bere E, Klepp K-I, Wind M. Longitudinal associations of energy balance-related behaviours and cross-sectional associations of clusters and body mass index in Norwegian adolescents. Public Health Nutr. 2010; 13(10A): 1716-21

Vangen S, Ellingsen L, Andersgaard AB, Jacobsen AF, Lorentzen B, Nyfløt LT, Rygh AB, Skulstad SM, Tappert C, Øian P. Maternal Deaths in Norway 2005-2009. Tidsskr Nor Laegeforen. 2014; 134(8): 836-9

von Plessen C, Grinde O, Gulsvik A. Incidence and prevalence of cryptogenic fibrosing alveolitis in a Norwegian community. Respir Med. 2003; 97(4): 428-35

von Soest T, Wichstrom L. Secular trends in depressive symptoms among Norwegian adolescents from 1992 to 2010. J Abnorm Child Psychol. 2014; 42(3): 403–15

Waatevik M, Skorge TD, Omenaas E, Bakke PS, Gulsvik A, Johannessen A. Increased prevalence of chronic obstructive pulmonary disease in a general population. Respir Med. 2013; 107(7): 1037-45

Weiderpass E, Braaten T, Magnusson C, Kumle M, Vainio H, Lund E, Adami H-O. A prospective study of body size in different periods of life and risk of premenopausal breast cancer. Cancer Epidemiol Biomarkers Prev. 2004; 13(7): 1121–7

WHO Collaborating Center for Food and Nutrition Policies, Department of Hygiene, Epidemiology, and Medical Statistics, University of Athens. Data Food Networking Project (DAFNE)

Wichstrøm L, Berg-Nielsen TS, Angold A, Egger HL, Solheim E, Sveen TH. Prevalence of psychiatric disorders in preschoolers. J Child Psychol Psychiatry. 2012; 53(6): 695-705

Wijnhoven TMA, van Raaij JMA, Spinelli A, Rito AI, Hovengen R, Kunesova M, Starc G, Rutter H, Sjöberg A, Petrauskiene A, O'Dwyer U, Petrova S, Farrugia Sant'angelo V, Wauters M, Yngve A, Rubana I-M, Breda J. WHO

European Childhood Obesity Surveillance Initiative 2008: weight, height and body mass index in 6-9-year-old children. Pediatr Obes. 2013; 8(2): 79-97

Wijnhoven TMA, van Raaij JMA, Spinelli A, Starc G, Hassapidou M, Spiroski I, Rutter H, Martos É, Rito AI, Hovengen R, Pérez-Farinós N, Petrauskiene A, Eldin N, Braeckevelt L, Pudule I, Kunešová M, Breda J. WHO European Childhood Obesity Surveillance Initiative: body mass index and level of overweight among 6-9-year-old children from school year 2007/2008 to school year 2009/2010. BMC Public Health. 2014; 14: 806

Wildman K, Bouvier-Colle M-H, MOMS Group. Maternal mortality as an indicator of obstetric care in Europe. BJOG. 2004; 111(2): 164-9

Winther A, Dennison E, Ahmed LA, Furberg AS, Grimnes G, Jorde R, Gjesdal CG, Emaus N. The Tromsø Study: Fit Futures: a study of Norwegian adolescents' lifestyle and bone health. Arch Osteoporos. 2014; 9: 185

World Bank. World Development Indicators - Gross Domestic Product (GDP). Washington DC, United States of America: World Bank

World Bank. World Development Indicators - Hospital Beds (per 1,000 People). Washington DC, United States of America: World Bank

World Energy Council. Survey of Energy Resources 2010. London, United Kingdom: World Energy Council, 2010

World Health Organization (WHO). Global Status Report on Road Safety 2009. Geneva, Switzerland: World Health Organization (WHO), 2009

World Health Organization (WHO). Global Status Report on Road Safety 2013. Geneva, Switzerland: World Health Organization (WHO), 2013

World Health Organization (WHO). Global Status Report on Road Safety 2015. Geneva, Switzerland: World Health Organization (WHO), 2015

World Health Organization (WHO). Global Status Report on Road Safety 2018. Geneva, Switzerland: World Health Organization (WHO), 2018

World Health Organization (WHO). Netherlands World Health Survey 2004. Geneva, Switzerland: World Health Organization (WHO), 2005

World Health Organization (WHO). Norway World Health Survey 2003. Geneva, Switzerland: World Health Organization (WHO), 2005

World Health Organization (WHO). WHO Distribution of Measles Cases by Country and by Month 2011-2020

World Health Organization (WHO). WHO Global Health Expenditure Database - National Health Accounts Indicators. Geneva, Switzerland: World Health Organization (WHO)

World Health Organization (WHO). WHO Global Health Observatory - Cholera: Number of Reported Cases by Country. Geneva, Switzerland: World Health Organization (WHO)

World Health Organization (WHO). WHO Global Health Observatory - Hospital Bed Density Data by Country . Geneva, Switzerland: World Health Organization (WHO)

World Health Organization (WHO). WHO Global Health Observatory - Recorded Alcohol Per Capita Consumption 1960-1979. Geneva, Switzerland: World Health Organization (WHO)

World Health Organization (WHO). WHO Global Health Observatory - Recorded Alcohol Per Capita Consumption 1980-1999. Geneva, Switzerland: World Health Organization (WHO)

World Health Organization (WHO). WHO Global Health Observatory - Recorded Alcohol Per Capita Consumption 2000-2009 by country. Geneva, Switzerland: World Health Organization (WHO)

World Health Organization (WHO). WHO Global Project on Anti-Tuberculosis Drug Resistance Surveillance Data 1988-2015

World Health Organization (WHO). WHO International Travel and Health, Annex 1, Countries With Risk of Yellow Fever Transmission and Countries Requiring Yellow Fever Vaccination 2017 Update. Geneva, Switzerland: World Health Organization (WHO), 2017

World Health Organization (WHO). WHO Mortality Database Version March 2017. Geneva, Switzerland: World Health Organization (WHO)

World Health Organization (WHO). WHO Report on the Global Tobacco Epidemic 2019. Geneva, Switzerland: World Health Organization (WHO), 2019

World Health Organization (WHO). WHO Tuberculosis Case Notifications. Geneva, Switzerland: World Health Organization (WHO)

World Health Organization (WHO). Global leprosy update, 2017: reducing the disease burden due to leprosy. Wkly Epidemiol Rec. 2018; 93(35): 445-56

World Health Organization Regional Office for Europe (EURO-WHO). Health Behaviour in School-aged Children: WHO Collaborative Cross-National Survey (HBSC) 2013/2014

World Health Organization Regional Office for Europe (EURO-WHO). Health Behaviour in School-aged Children: WHO Collaborative Cross-National Survey (HBSC) 2013/2014

World Health Organization Regional Office for Europe (EURO-WHO). Health Behaviour in School-aged Children: WHO Collaborative Cross-National survey/study (HBSC) 1997

World Health Organization Regional Office for Europe (EURO-WHO). Health Behaviour in School-aged Children: WHO Collaborative Cross-National survey/study (HBSC) 2001

World Health Organization Regional Office for Europe (EURO-WHO). Health Behaviour in School-aged Children: WHO Collaborative Cross-National survey/study (HBSC) 2005-2006

World Health Organization Regional Office for Europe (EURO-WHO). Health Behaviour in School-aged Children: WHO Collaborative Cross-National survey/study (HBSC) 2009-2010

World Health Organization Regional Office for Europe (WHO/Europe). European Hospital Morbidity Database, 2008. Copenhagen, Denmark: World Health Organization Regional Office for Europe (WHO/Europe)

World Health Organization Regional Office for Europe (WHO/Europe). Gender and Health in Adolescence. Copenhagen, Denmark: World Health Organization Regional Office for Europe (WHO/Europe), 1999

World Health Organization Regional Office for Europe (WHO/Europe). Health and Health Behaviour Among Young People: Health Behaviour in School-Aged Children: A WHO Cross National Study (HBSC) International Report. Copenhagen, Denmark: World Health Organization Regional Office for Europe (WHO/Europe), 2000

World Health Organization Regional Office for Europe (WHO/Europe). Inequalities in Young People's Health: HBSC International Report from the 2005-2006 Survey. Copenhagen, Denmark: World Health Organization Regional Office for Europe (WHO/Europe), 2008

World Health Organization Regional Office for Europe (WHO/Europe). Social Determinants of Health and Well-being Among Young People: Health Behaviour in School-aged Children (HBSC) Study: International Report from the 2009/2010 Survey. Copenhagen, Denmark: World Health Organization Regional Office for Europe (WHO/Europe), 2012

World Health Organization Regional Office for Europe (WHO/Europe). The Health of Youth: A Cross-National Survey. Copenhagen, Denmark: World Health Organization Regional Office for Europe (WHO/Europe), 1996

World Health Organization Regional Office for Europe (WHO/Europe). WHO/Europe Centralized Information System for Infectious Disease - Chlamydia. Copenhagen, Denmark: World Health Organization Regional Office for Europe (WHO/Europe)

World Health Organization Regional Office for Europe (WHO/Europe). WHO/Europe Centralized Information System for Infectious Disease - Gonorrhoea. Copenhagen, Denmark: World Health Organization Regional Office for Europe (WHO/Europe)

World Health Organization Regional Office for Europe (WHO/Europe). WHO/Europe Centralized Information System for Infectious Disease - Syphilis, Early. Copenhagen, Denmark: World Health Organization Regional Office for Europe (WHO/Europe)

World Health Organization Regional Office for Europe (WHO/Europe). Young People's Health in Context. Health Behaviour in School-aged Children (HBSC) Study: International Report from the 2001-2002 Survey. Copenhagen, Denmark: World Health Organization Regional Office for Europe (WHO/Europe), 2004

Ødegård E, Amundsen EJ, Kielland KB. Fatal overdoses and deaths by other causes in a cohort of Norwegian drug abusers – a competing risk approach. Drug Alcohol Depend. 2007; 89(2-3): 176-82

Øverby NC, Lillegaard ITL, Johansson L, Andersen LF. High intake of added sugar among Norwegian children and adolescents. Public Health Nutr. 2004; 7(2): 285–93
